# Design, Synthesis, and Anticancer and Antibacterial Activities of Quinoline-5-Sulfonamides

**DOI:** 10.3390/molecules29174044

**Published:** 2024-08-26

**Authors:** Andrzej Zieba, Dominika Pindjakova, Malgorzata Latocha, Justyna Plonka-Czerw, Dariusz Kusmierz, Alois Cizek, Josef Jampilek

**Affiliations:** 1 Department of Organic Chemistry, Faculty of Pharmaceutical Sciences in Sosnowiec, Medical University of Silesia, Jagiellonska 4, 41-200 Sosnowiec, Poland; 2 Department of Infectious Diseases and Microbiology, Faculty of Veterinary Medicine, University of Veterinary Sciences Brno, Palackeho 1946/1, 612 42 Brno, Czech Republic; pindjakova.dominika@gmail.com (D.P.); cizeka@vfu.cz (A.C.); 3 Department of Cell Biology, Faculty of Pharmaceutical Sciences in Sosnowiec, Medical University of Silesia, Jednosci 9, 41-200 Sosnowiec, Poland; mlatocha@sum.edu.pl (M.L.); jplonka@sum.edu.pl (J.P.-C.); dkusmierz@sum.edu.pl (D.K.); 4 Institute of Chemistry, University of Silesia, Szkolna 9, 40-007 Katowice, Poland

**Keywords:** 8-hydroxyquinoline, acetylene derivatives, 1,2,3-triazole, synthesis, anticancer activity, antibacterial activity, cytotoxicity

## Abstract

A series of new unique acetylene derivatives of 8-hydroxy- and 8-methoxyquinoline- 5-sulfonamide **3a**–**f** and **6a**–**f** were prepared by reactions of 8-hydroxy- and 8-methoxyquinoline- 5-sulfonyl chlorides with acetylene derivatives of amine. A series of new hybrid systems containing quinoline and 1,2,3-triazole systems **7a**–**h** were obtained by reactions of acetylene derivatives of quinoline-5-sulfonamide **6a**–**d** with organic azides. The structures of the obtained compounds were confirmed by ^1^H and ^13^C NMR spectroscopy and HR-MS spectrometry. The obtained quinoline derivatives **3a**–**f** and **6a**–**f** and 1,2,3-triazole derivatives **7a**–**h** were tested for their anticancer and antimicrobial activity. Human amelanotic melanoma cells (C-32), human breast adenocarcinoma cells (MDA-MB-231), and human lung adenocarcinoma cells (A549) were selected as tested cancer lines, while cytotoxicity was investigated on normal human dermal fibroblasts (HFF-1). All the compounds were also tested against reference strains *Staphylococcus aureus* ATCC 29213 and *Enterococcus faecalis* ATCC 29212 and representatives of multidrug-resistant clinical isolates of methicillin-resistant *S. aureus* (MRSA) and vancomycin-resistant *E. faecalis*. Only the acetylene derivatives of 8-hydroxyquinoline-5-sulfonamide **3a**–**f** were shown to be biologically active, and 8-hydroxy-*N*-methyl-*N*-(prop-2-yn-1-yl)quinoline-5-sulfonamide (**3c**) showed the highest activity against all three cancer lines and MRSA isolates. Its efficacies were comparable to those of cisplatin/doxorubicin and oxacillin/ciprofloxacin. In the non-cancer HFF-1 line, the compound showed no toxicity up to an IC_50_ of 100 µM. In additional tests, compound **3c** decreased the expression of H3, increased the transcriptional activity of cell cycle regulators (P53 and P21 proteins), and altered the expression of BCL-2 and BAX genes in all cancer lines. The unsubstituted phenolic group at position 8 of the quinoline is the key structural fragment necessary for biological activity.

## 1. Introduction

Quinoline-based compounds, especially 8-hydroxyquinolines (8-HQs), have a wide range of biological properties; they can be considered as privileged structures of multi-target agents [1,2,3,4]. A simple 8-HQ scaffold has unique physicochemical properties and affords the possibility of a large number of modifications (via target or diversity-directed synthesis) [4,5,6,7,8]. On the other hand, all these compounds show a mechanism of action which is complex and difficult to determine e.g., [4,6,7,8,9,10] based on the ability to chelate different metals [11,12,13,14,15,16] and affect different enzymatic systems [7,17,18,19,20,21,22,23,24,25]. Despite this, quinoline-based compounds form important scaffolds in medicinal chemistry [2,4,6,10], mainly used for the design of anti-infective [7,10,18,21,22,23,24,25,26,27,28,29,30,31,32,33,34,35] and anticancer [13,16,17,20,36,37,38,39] drugs. In this context, the discovery of camptothecin [40], a quinoline alkaloid of plant origin, whose anticancer properties became an incentive for many researchers to search for anticancer drugs in the group of quinoline derivatives, was of great importance. Halogenated derivatives of 8-HQs are used as generally available drugs [41,42,43,44,45].

In addition to 8-HQs, 1,2,3-triazole also forms an important pharmacophore [46,47,48,49,50,51,52] due to its unique chemical and structural properties. This heterocycle has a high dipole moment and is able to form hydrogen bonds, which play an important role in modulating the bioavailability and solubility of biologically active compounds and can be beneficial in binding to molecular targets. Thus, this 1,2,3-triazole scaffold is effectively used in designing many types of agents with different biological activities [46,47,48,49,50,51,52], but especially with antifungal [53,54,55], antibacterial [56,57,58,59], antiviral [60,61], and anticancer [62,63,64,65] effects. Furthermore, the propargyl (acetylene) tail / propargyl sulfonamide fragment, for which significant biological properties have also been described, is an important fragment in the molecules discussed in this contribution [66,67,68,69,70,71,72,73].

Based on previous experience with ring-substituted 8-HQ derivatives [12,22,28,29,30,32,44,45] and propargyl-substituted heterocycles [67,68,69], new acetylene sulfonamides and 1,2,3-triazole hybrids of 8-HQ were designed and synthesized, and their antiproliferative and antibacterial activities were investigated. Despite the abundance of sulfonamide derivatives of 8-HQ described in the literature e.g., [18,19,44] and many scientific centers around the world engaged in the synthesis of new 8-HQ derivatives and evaluation of their biological properties, there are no reports of the synthesis of acetylene derivatives of 8-hydroxyquinoline-5-sulfonamides. In addition, the main directions of structural modifications of the 8-HQ scaffold consisted in the introduction of different pharmacophoric systems into positions 2, 5, and 7 of the quinoline core and into the oxygen atom of the hydroxyl group.

Thus, the investigation of new substituted hybrid systems combining 8-HQ, sulfonamide, acetylene, and 1,2,3-triazole expands the library of 8-HQ-based derivatives and the knowledge of their biological activities in relation to selected cancer cell lines and Gram-positive bacteria.

## 2. Results and Discussion

### 2.1. Chemistry

**Scheme 1:**
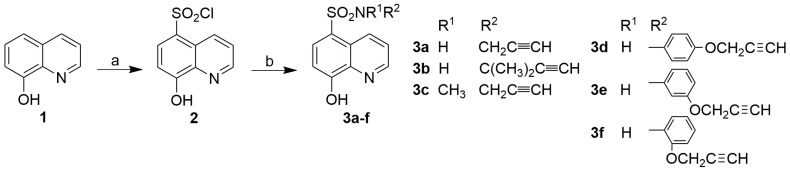
Preparation of 8-hydroxyquinoline-5-sulfonamides (**3**). Reagents and conditions: (a) ClSO_3_H, room temp.; (b) NHR^1^R^2^, CH_3_CN, room temp.

The reaction of 8-HQ (**1**) with chlorosulfonic acid gave 8-hydroxyquinoline-5-sulfonyl chloride (**2**). The reaction of this chloride **2** with appropriate amines containing an acetylene moiety led to the corresponding 8-hydroxyquinoline-5-sulfonamides (**3a**–**f**) (Scheme 1). The reactions were carried out in anhydrous acetonitrile at room temperature using four moles of amine per one mole of sulfonic chloride. The structure of compounds of series **3** was modified by using different amines containing an acetylene moiety: propargylamine (**a**), 2-methylbut-3-yn-2-amine (**b**), *N*-methylpropargylamine (**c**), and three isomeric 2-/3-/4-(propargyloxy)anilines (**d**–**f**).

Papers focused on the reactions of acetylenic sulfonamide derivatives with organic azides have been published, and the 1,2,3-triazole derivatives obtained in this way showed interesting biological properties [51,52]. It seemed that the designed acetylenic quinoline-5-sulfonamide derivatives **3a**–**f** would be suitable substrates for obtaining hybrid systems containing a quinoline-5-sulfonamide system and a 1,2,3-triazole scaffold. We carried out a series of reactions of compounds **3a**–**f** with organic azides. The reactions were performed in DMF/H_2_O in the presence of a copper catalyst (CuSO_4_ · 5H_2_O, sodium ascorbate) at room temperature. However, the analysis of the obtained post-reaction mixtures using mass spectrometry did not reveal the presence or even trace amounts of the expected triazole derivatives of quinoline-5-sulfonamide. The 8-hydroxyquinoline system exhibits chelating properties, as mentioned above, which may cause interactions with the catalyst system used, resulting in its ineffectiveness. Thus, in the next stage of this work, we decided to protect the phenolic group by the methylation reaction and verify whether/how such a structural modification will affect the course of the cyclization reaction.

**Scheme 2:**
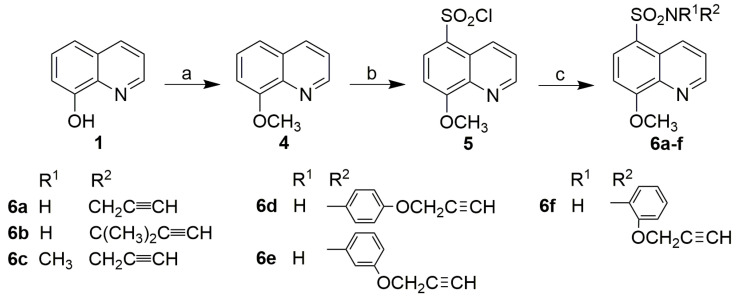
Preparation of 8-methoxyquinoline-5-sulfonamides (**6**). Reagents and conditions: (a) NaH, CH_3_I, DMF, room temp.; (b) ClSO_3_H, room temp.; (c) NHR^1^R^2^, CH_3_CN, (C_2_H_5_)_3_N, room temp.

The *O*-methylation reaction of 8-hydroxyquinoline was carried out under anhydrous conditions in DMF solution in the presence of a stoichiometric amount of sodium hydride. Then, a sulfonation reaction (analogous to sulfonyl chloride **2**) of prepared 8-methoxyquinoline (**4**) led to 8-methoxyquinoline-5-sulfonyl chloride (**5**), which, with acetylenamine derivatives, gave corresponding 8-methoxyquinoline-5-sulfonamides **6a**–**f** in high yields (Scheme 2). The reaction was performed in anhydrous acetonitrile using a two-fold molar excess of amine and triethylamine as the hydrogen chloride acceptor.

**Scheme 3:**
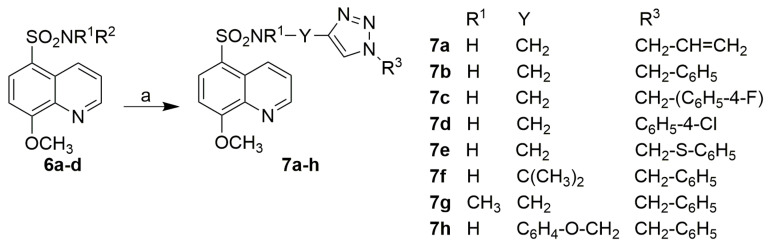
Synthesis of 1,2,3-triazole 8-methoxyquinoline derivatives (**7**). Reagents and conditions: (a) N_3_–R^3^, DMF/H_2_O, CuSO_4_ · 5H_2_O, sodium ascorbate, room temp.

The next stage of the work involved coupling reactions of acetylenic sulfonamide derivatives **6a**–**d** with organic azides. The reactions were carried out in DMF/H_2_O in the presence of a copper catalyst (CuSO_4_ · 5H_2_O, sodium ascorbate); see Scheme 3. Commercial azides were generally used. For the synthesis of allyl derivative **7a**, allyl azide was prepared by reacting allyl bromide with sodium azide immediately prior to coupling with propargyl derivative **6a**. The synthesis was performed according to a one-pot procedure without isolating the azide from the reaction mixture. The reactions proceeded readily in high yields at room temperature. An exception is the reaction of propargyl derivative **6a** with an aromatic azide (1-azido-4-chlorobenzene), which provided a very low yield at room temperature. The reaction carried out in a microwave reactor at 100 °C led to corresponding 1,2,3-triazole derivative **5d** in 57% yield. The structures of the obtained compounds were confirmed by ^1^H and ^13^C-NMR spectroscopy and HR-MS spectrometry (see Supplementary Materials, Figures S1–S69). In the HR-MS spectrum of compound **2**, there is an additional signal at *m*/*z* = 226.0165. This is the effect of hydrolysis of 8-hydroxyquinoline-5-sulfonyl chloride occurring during the ionization of the sample using the ESI method. The signal corresponds to the molecular mass of 8-hydroxyquinoline-5-sulfonic acid [M+H]^+^.

### 2.2. Biological Screening

#### 2.2.1. In Vitro Cell Viability

The synthesized compounds were tested using three cancer cell lines—human amelanotic melanoma (C-32), human breast adenocarcinoma (MDA-MB-231), and human lung adenocarcinoma (A549)—and, for comparison, normal human dermal fibroblasts (HFF-1). The WST-1 (water-soluble tetrazole salt) assay was used to evaluate the effect of the synthesized compounds on the viability of cultured cells. IC_50_ values for cell cultures exposed to test compounds for 72 h are shown in Table 1 and Table 2.

As can be seen from the IC_50_ values in Table 1 and Table 2, derivatives **3a**–**f**, i.e., with a free 8-phenolic group, were the most effective against the tested cancer lines. Methylation of the phenolic moiety (series **6**) generally resulted in a significant loss of activity, which is most evident in the effect on breast (MDA-MB-231) and lung (A549) adenocarcinoma cell lines. Compounds **3a**–**f** showed insignificantly different activity against both C-32 and A549 lines. MDA-MB-231 cells were the least sensitive to the discussed derivatives, but compound **3c** achieved excellent efficacy, comparable to that of the clinically used drugs cisplatin and doxorubicin.

Overall, it can be concluded that the inhibition of the proliferation of the non-cancerous HFF-1 line by the active compounds of series **3** was significantly worse than the growth inhibition of all three used cancer lines. For compounds **3a**–**c** (substitution with propargyl), the disubstitution of the alpha carbon of propargyl (compound **3b**) led to a significant decrease in activity, while, on the contrary, the disubstituted nitrogen of the sulfonamide group (compound **3c**) resulted in the most effective derivative of the investigated compounds. Of the trio of propargyloxyanilides **3d**–**f**, the *meta*-isomer was the most effective. Shifting the propargyloxy chain to the *para* position caused a decrease in activity, while the *ortho*-isomer **3f** demonstrated increased general cytotoxicity, which was also observed against the non-cancer HFF-1 line. It should be noted that a similar trend, when cytotoxicity increased even for non-cancer cells in the case of spatially close substitution of the propargyloxy chain to the nitrogen atom, was reported by Kisiel-Nawrot et al. [68].

As mentioned above, methylation of the 8-HQ phenolic group resulted in a significant loss of activity, with the C-32 line again being the most sensitive to these methoxy derivatives. The most active derivatives were **6d** and **6e**. Unfortunately, the introduction of 1,2,3-triazole into the molecule (series **7**) led to a loss of activity, contrary to our expectation, when the introduction of 1,2,3-triazole onto the quinobenzothiazine scaffold resulted in a significant increase in anticancer potency [68,69].

#### 2.2.2. Additional Antiproliferative Tests

Since compound **3c** showed the best activity against all three cancer lines, its effects on cultured cell lines were investigated in more detail. The effect of the compound on cell viability was determined using, in addition to the WST-1 assay, the crystal violet assay and the LDH (lactate dehydrogenase) assay. Furthermore, a wound healing test (indicator of cell proliferation and migration capacity) and assessment of gene expression (in the transcription phase) of BCL-2, BAX, TP53, P21, and H3 were performed.

##### Violet-WST-LDH

**Figure 1:**
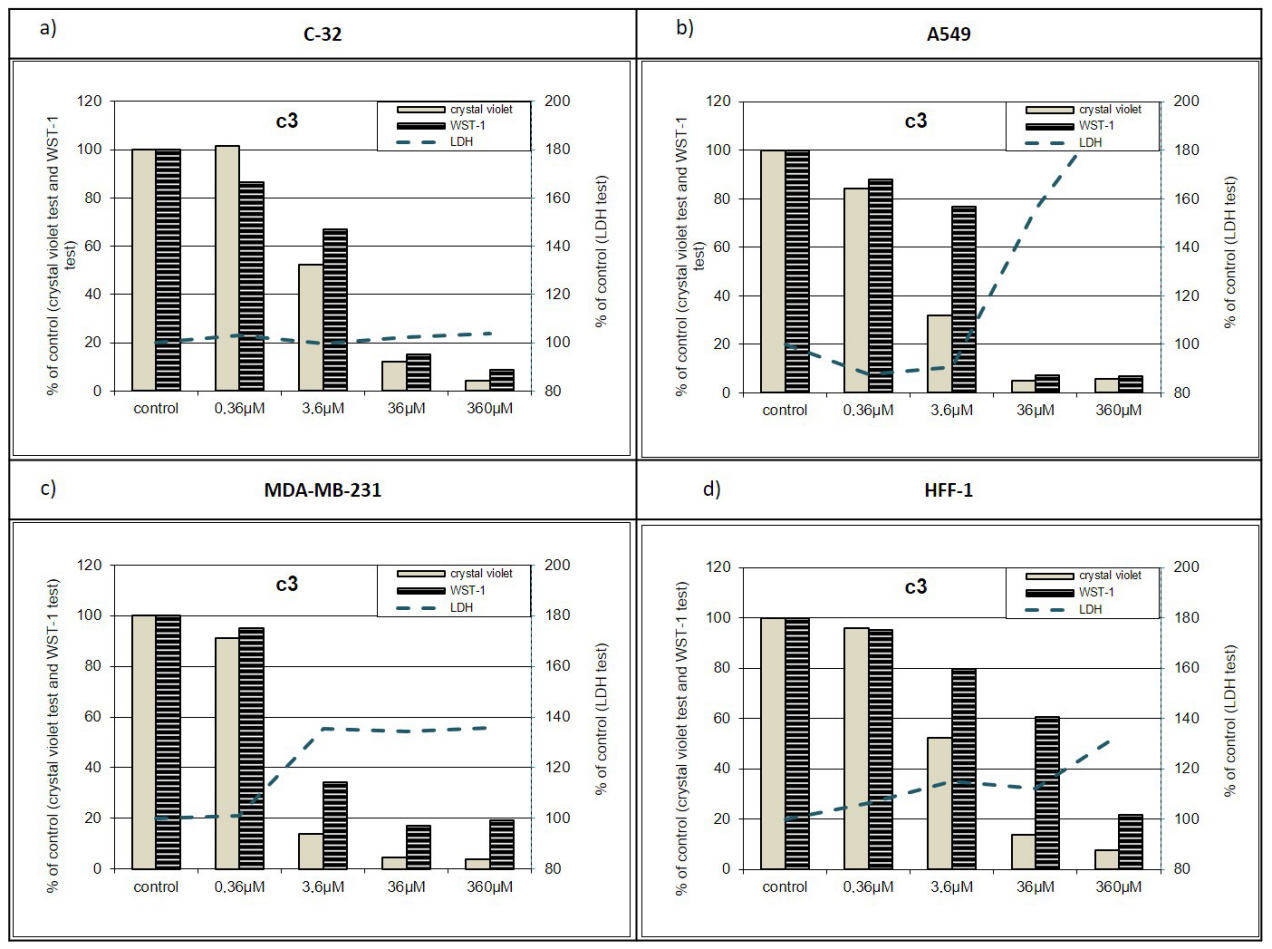
Effect of derivative **3c** on cancer cell lines C-32, A549, and MDA-MB-231 and normal HFF-1 cells—cell number (crystal violet assay), metabolically active cells (WST-1 assay), and number of dead cells in culture (LDH test).

Cell viability and cytotoxicity assays of compound **3c** showed a significant decrease in cell number in cultures exposed to **3c** in medium above 0.3 µM, particularly in A549 and MDA-MB-231 cells (Figure 1b,c). The differences in the results of the crystal violet and WST-1 tests indicate that some of the cells that still transform the WST-1 dye into colored products lose their adhesive abilities and are already separated from the culture vessel (they are washed away in the violet test—hence, the lower absorbance values than in WST-1), leading to their death in a short time. An increase in the number of dead cells is shown by the absorbance line in the LDH test.

##### Wound Healing Assay

**Figure 2:**
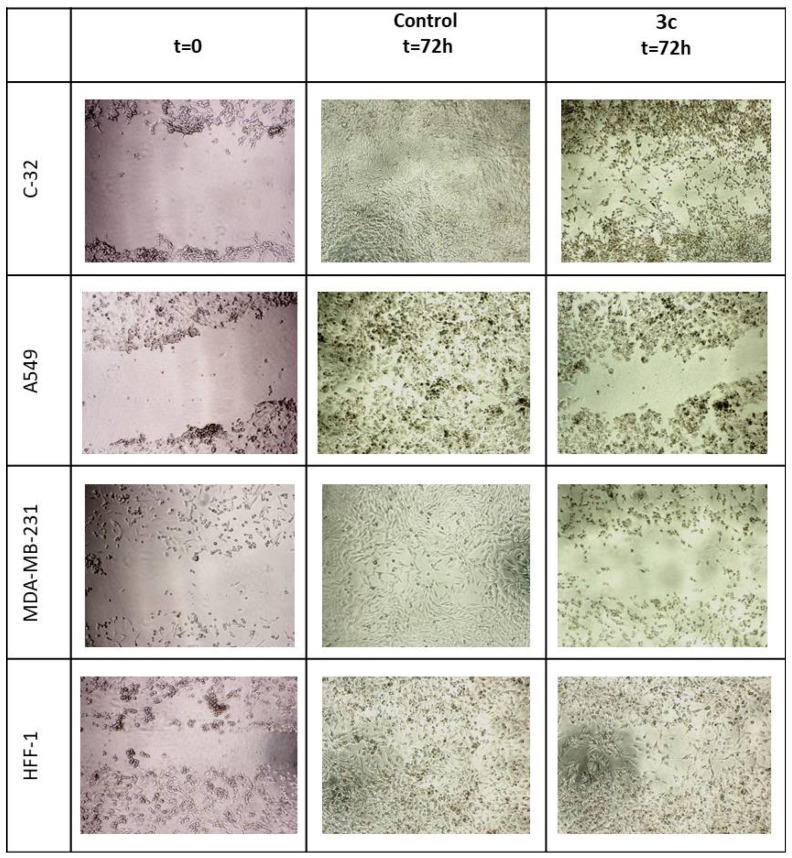
Wound healing assay. Cultures of C-32, A549, MDA-MB-231, and HFF-1 cells at times: t = 0 (moment of making a scratch and replacing the medium with fresh—control versus addition of **3c**), t = 72 (72 h after making the scratch—control versus addition of **3c**).

Photographs of the cell culture (control and culture exposed to compound **3c** at a concentration of 3 µM) were taken after scraping the bottom of the vessel with a pipette tip and then after 72 h of incubation (Figure 2). Image analysis of cell culture 72 h after scratching in cell culture indicates a reduction in proliferative activity and migration capacity of all cells exposed to compound **3c**. In control cultures, the cell-free surface of the vessel (scraped with a pipette tip) becomes overgrown with cells, while in cultures supplemented with medium containing 3 µM of compound **3c**, empty spots are visible. In the case of A549 and MDA-MB-231 cells, entire cell-free regions are clearly visible.

##### Transcriptional Activity

**Figure 3:**
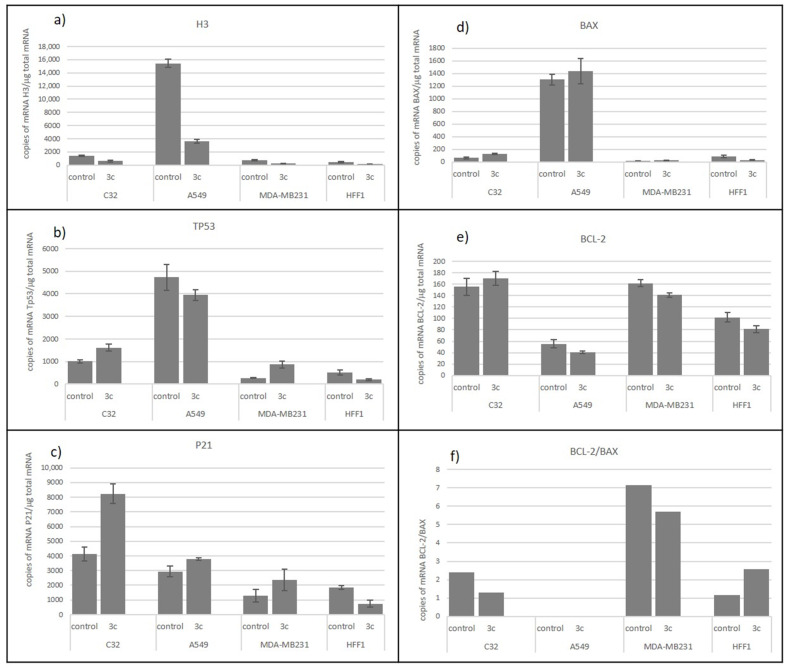
Effect of compound **3c** on transcriptional activity: H3 (**a**); TP53 (**b**); P21 (**c**), BAX (**d**,**f**) and BCL-2 (**e**,**f**) in C-32, A549, MDA-MB-231, and HFF-1 cells.

The study investigated the effect of compound **3c** on the transcriptional activity of genes encoding a proliferation marker (histone H3), cell cycle regulators (P53 and P21), and genes encoding proteins involved in the mitochondrial apoptosis pathway (BCL-2 and BAX) in C-32, A549, MDA-MB-231, and HFF-1 cells. Cultures were exposed to **3c** at a concentration of 36 µM in medium for 24 h. Figure 3 shows the pooled mRNA copy number results for individual genes of control compound **3c** in C32, A549, MDA-MB-231, and HFF-1 cell cultures. A beneficial effect of **3c** was observed in all the lines in the sense of finding an effective agent to destroy cancer cells: a) reduction in H3 expression (proliferation marker) in all the cultures with **3c**, b) and c) an increase in the transcriptional activity of cell cycle regulators (P53 and P21 proteins), d) and e) changes in the expression of BCL-2 and BAX genes, leading to f) a shift in the BCL-2/BAX ratio (the ratio of the number of BCL-2 and BAX gene copies) in favor of BAX, which indicates the possibility of cells entering the path of apoptosis.

#### 2.2.3. In Vitro Antimicrobial Activity

Since there is a general decrease in immunity during the treatment of oncological patients, i.e., patients are more susceptible to infectious diseases, it is advantageous if anticancer agents show antimicrobial properties at the same time [74,75]. Therefore, the effects of the compounds were also investigated against Gram-positive bacteria. For this purpose, the susceptible collection strains of aerobic *Staphylococcus aureus* ATCC 29213 and facultatively aerobic *Enterococcus faecalis* ATCC 29212 and resistant clinical isolates of methicillin-resistant *S. aureus* (MRSA) SA 3202, SA 630 carrying the *mecA* gene [76,77], and vancomycin-resistant isolates of *E. faecalis* (VRE) 342B, 368, and 725B carrying the *vanA* gene [78] were selected. Thus, the selection of the studied bacterial strains was adopted following the CLSI (National Committee for Clinical Laboratory Standards) international reference methodologies [79].

The activities of the individual derivatives were expressed as minimum inhibitory concentrations (MICs) and minimum bactericidal concentrations (MBCs) and are shown in Table 3 together with the values of the antibiotics used as standards for comparison. Compounds exhibiting bactericidal activity against a particular tested strain meet the condition of MIC/MBC ≤ 4 [80]. MBC values of bactericidal compounds are indicated in bold in Table 3.

As in the case of anticancer activity, only series **3** was active, i.e., after blocking the phenolic group at position 8 of quinoline with methyl (series **6**), there was a total loss of antibacterial activity (MIC > 256 µg/mL). Even triazine substitution (series **7**) did not improve activity (MICs > 256 µg/mL). Therefore, the MIC values of series **6** and **7** are not reported as completely unimpressive. It is important to add that all the derivatives had only antistaphylococcal activity. On the other hand, all were bactericidal (see bolded MBC values in Table 3). Derivative **3c** demonstrated the highest antistaphylococcal potency (although it can be rated as moderately active) as well as anticancer activity. Compound **3c** had equal activity against both susceptible strains and MRSA isolates (it was more potent than clinically used antibiotics), suggesting that the presence of the *mecA* gene (which encodes an alternative transpeptidase and causes resistance to methicillin [76,77]) in MRSA does not affect the activity of this compound. Therefore, the specific activity against *Staphylococcus* sp. is a matter for speculation. The isomeric propargyloxyanilides **3d**–**3e** showed identical activity.

The compounds were more active against aerobic staphylococci than facultatively aerobic enterococci, so an MTT assay was performed to verify whether the molecules caused inhibition of respiration. The MTT assay can be used to assess cell growth by measuring respiration. Bacterial cell respiratory activity (which is ultimately reflected in their viability) of less than 70% after exposure to the MIC values for each test compound is considered as a positive result for this test. This low level of cellular oxidative metabolism indicates inhibition of cell growth by inhibition of respiration [81,82]. The lowest multiples of the MIC values by which inhibition of *S. aureus* ATCC 29213 viability [%] greater than 70% was achieved are shown in Table 4. The compounds showed inhibition of respiration at 1× or 2× MIC ranging from 73 to 95%, indicating that they are able to affect the respiratory chain (e.g., compared to ciprofloxacin 95% inhibition at 32× MIC), but this is not their main mechanism of action. Therefore, the antibacterial activity of the discussed compounds cannot be explained solely on the basis of respiration inhibition, so it is very difficult to draw a conclusion regarding the actual mechanism of action, since 8-HQs are multi-target agents, i.e., they affect several different targets at once (as discussed in the section dedicated to anticancer activity), which subsequently leads to the death of the treated resistant microorganisms.

As with anticancer activity, it is difficult to speculate on the structure-activity relationship for this small series of these moderately active compounds. However, it is certain that methylation of the phenolic group of 8-HQ caused a loss of antimicrobial activity that was not restored even by triazine substitution.

## 3. Materials and Methods

### 3.1. Chemistry

Melting points are uncorrected. NMR spectra were recorded using a Bruker Ascend 600 spectrometer (Bruker, Billerica, MA, USA). To assign the structures, the following 2D experiments were employed: ^1^H/^13^C gradient-selected HSQC and HMBC sequences. Standard experimental conditions and standard Bruker programs were used. The ^1^H NMR and ^13^C NMR spectral data are provided relative to the TMS signal at 0.0 ppm. HR mass spectra were recorded with a Bruker Impact II (Bruker, Billerica, MA, USA).

### 3.2. Synthesis

#### 3.2.1. Synthesis of 8-Hydroxyquinoline-5-Sulfonyl Chloride (**2**)

A flask containing 1.450 g (10 mmol) of 8-hydroxyquinoline (**1**) was placed in an ice bath, and 5.850 g (50 mmol) of chlorosulfonic acid was added dropwise. The resulting reaction mixture was stirred at room temperature for 24 h. The reaction mixture was poured onto 150 g of ice and extracted with ethylene chloride (3 × 150 mL). The combined extracts were dried over anhydrous sodium sulfate. After evaporating the solvent in vacuo, the obtained product was stored under argon at a temperature of 4–8 °C.

*8-Hydroxyquinoline-5-sulfonyl chloride* (**2**): yield: 67%; ^1^H NMR (DMSO, 600 MHz), δ (ppm): 7.44–7.47 (d, *J* = 8.4 Hz, 1H, H_arom_), 8.08–8.11 (d, *J* = 8.4 Hz, 1H, H_arom_), 8.16–8.21 (dd, *J* = 8.4 Hz, *J* = 5.4 Hz, 1H, H_arom_), 9.11–9.14 (d, *J* = 5.4 Hz, 1H, H_arom_), 9.80–9.84 (d, *J* = 8.4 Hz, 1H, H_arom_), 12.50 (s, 1H OH); ^13^C NMR (DMSO, 150.9 MHz), δ (ppm): 114.33, 122.99, 126.64, 128.93, 129.42, 135.82, 144.37, 146.31, 149.33; ESI-HRMS Calcd. for C_9_H_5_NO_3_S^35^Cl ([M + H]^+^) = 243.9834 found: 243.9834, Calcd. for C_9_H_5_NO_3_S^37^Cl ([M + H]^+^) = 245.9805 found: 245.9795.

#### 3.2.2. Synthesis of 8-Hydroxyquinoline-5-Sulfonamides (**3**)

An amount of 20 mmol of the appropriate amine was added to a suspension of 1.220 g (5 mmol) of sulfonyl chloride **2** in 15 mL of anhydrous acetonitrile. The resulting reaction mixture was stirred at room temperature for 24 h. The mixture was poured into 100 mL of water and extracted with chloroform (3 × 20 mL). The combined extracts were dried over anhydrous sodium sulfate. After evaporation of the solvent in vacuo, the crude product was purified by recrystallization from methanol.

*8-Hydroxy-N-(prop-2-yn-1-yl)quinoline-5-sulfonamide* (**3a**): yield: 78%; m.p. = 143–145 °C; ^1^H NMR (DMSO, 600 MHz), δ (ppm): 2.82-2.85 (t, *J* = 2.4 Hz, 1H, CH), 3.66–3.68 (d, *J* = 2.4 Hz, 2H, CH_2_), 7.13–7.16 (d, *J* = 8.4 Hz, 1H H_arom_), 7.75–7.78 (dd, *J* = 8.4 Hz, *J* = 4.2 Hz, 1H, H_arom_), 8.07–8.10 (d, *J* = 8.4 Hz, 1H, H_arom_), 8.28–8.32 (t, *J* = 6 Hz, 1H, NH), 8.93–8.98 (m, 2H, H_arom_); ^13^C NMR (DMSO, 150.9 MHz), δ (ppm): 31.98 (CH_2_), 74.60 (CH), 79.90 (CCH), 109.92, 123.58, 125.07, 125.27, 132.18, 133.89, 138, 90, 149.17, 158.55; ESI-HRMS Calcd. for C_12_H_11_N_2_O_3_S ([M + H]^+^) = 263.0490, found: 263.0514.

*8-Hydroxy-N-(1,1-dimethylprop-2-yn-1-yl)quinoline-5-sulfonamide* (**3b**): yield: 74%; m.p. = 82–85 °C; ^1^H NMR (DMSO, 600 MHz), δ (ppm): 1.38 (s, 6H, CH_3_), 2.74 (s, 1H, CH), 7.12–7.14 (d, *J* = 8.4 Hz, 1H, H_arom_), 7.72–7.75 (m, 1H, H_arom_), 8.08–8.13 (m, 2H, NH, H_arom_), 8.94–8.97 (m, 1H, H_arom_), 8.97–9.01 (m, 1H, H_arom_); ^13^C NMR (DMSO, 150.9 MHz), δ (ppm): 30.91 (CH_3_), 49.05 (C(CH_3_)_2_), 73.17 (CH), 79.64 (CCH), 109.73, 123.36, 125.48, 126.98, 132.28, 133.97, 138.72, 148.99, 158.32; ESI-HRMS Calcd. for C_14_H_15_N_2_O_3_S ([M + H]^+^) = 291.0803, found: 291.0804.

*8-Hydroxy-N-methyl-N-(prop-2-yn-1-yl)quinoline-5-sulfonamide* (**3c**): yield: m.p. = 145–147 °C; 65%; ^1^H NMR (CD_3_OD, 600 MHz), δ (ppm): 2.18–2.20 (t, *J* = 3 Hz, 1H, CH), 3.23 (s, 3H, CH_3_), 3.73–3.75 (d, *J* = 3 Hz, 2H, CH_2_), 7.15–7.8 (d, *J* = 8.4 Hz, 1H, H_arom_), 7.68–7.72 (m, 1H, H_arom_), 8.20–8.22 (d*, J* = 8.4 Hz, 1H, H_arom_), 8.92–8.94 (m, 1H, H_arom_), 9.03–9.07 (m, 1H, H_arom_); ^13^C NMR (CDCl_3_, 150.9 MHz), δ (ppm): 33.11 (CH_2_), 38.11 (CH_3_), 71.07 (CH), 72.91 (CCH), 107.19, 121.75, 122.51, 124.36, 132.47, 133.40, 137.01, 147.45, 156.06; ESI-HRMS Calcd. for C_13_H_13_N_2_O_3_S [M + H]^+^) = 277.0646, found: 277.0606.

*8-Hydroxy-N-[4-(prop-2-yn-1-yloxy)phenyl]quinoline-5-sulfonamide* (**3d**): yield: 68%; m.p. = 119–121 °C; ^1^H NMR (CDCl_3_, 600 MHz), δ (ppm): 2.50–2.52 (t, *J* = 2.4 Hz, 1H, CH), 4.61–4.62 (d, *J* = 2.4 Hz, 2H, CH_2_), 6.48 (m, 1H, NH), 6.75–6.79 (d, *J* = 9 Hz, 2H, H_arom_), 6.83–6.87 (d, *J* = 9 Hz, 2H, H_arom_), 7.12–7.15 (m, 1H, H_arom_), 7.55–7.59 (dd, *J* = 8.4 Hz, *J* = 4.2 Hz, 1H, H_arom_), 8.13–8.16 (m, 1H, H_arom_), 8.65–8.90 (m, 2H, H_arom_); ^13^C NMR (CDCl_3_, 150.9 MHz), δ (ppm): 55.99 (CH_2_), 75.79 (CH), 78.10 (CCH), 108.33, 115.38, 115.53, 121.74, 123.56, 125.99, 129.18, 133.26, 133.88, 137,76, 148.38, 156.18, 156.93; ESI-HRMS Calcd. for C_18_H_15_N_2_O_4_S ([M + H]^+^) = 355.0752, found: 355.0735.

*8-Hydroxy-N-[3-(prop-2-yn-1-yloxy)phenyl]quinoline-5-sulfonamide* (**3e**): yield: 61%; m.p. = 135–137 °C; ^1^H NMR (DMSO, 600 MHz), δ (ppm): 3.53–3.55 (t, *J* = 2.4 Hz, 1H, CH), 4.63–4.66 (m, 2H, CH_2_), 6.55–6.61 (m, 2H, H_arom_), 6.63–6.66 (m, 1H, H_arom_), 7.07–7.08 (m, 1H, H_arom_), 7.2–7.16 (d, *J* = 8.4 Hz, 1H, H_arom_), 7.75–7.79 (dd, *J* = 9 Hz, *J* = 4.2 Hz, 1H, H_arom_), 8.16–8.20 (d, *J* = 8.4 Hz, 1H, H_arom_), 8.96–8.98 (dd, *J* = 4.2 Hz, *J* = 1.8 Hz, 1H, H_arom_), 9.01–9.06 (dd, *J* = 8.4 Hz, *J* = 1.8 Hz, 1H, H_arom_), 10.59 (s, 1H, OH), 11.02 (s, 1H, NH); ^13^C NMR (DMSO, 150.9 MHz), δ (ppm): 55.80 (CH_2_), 78.77 (CH), 79.44 (CCH), 106.11, 109.91, 110.06, 112.119, 1213.72, 123.90, 125.03, 130.39, 133.24, 133.37, 138.71, 139.26, 149.41, 158.10, 158.97; ESI-HRMS Calcd. for C_18_H_15_N_2_O_4_S ([M + H]^+^) = 355.0752, found: 355.0748.

*8-Hydroxy-N-[2-(prop-2-yn-1-yloxy)phenyl]quinoline-5-sulfonamide* (**3f**): yield: 55%; m.p. = 153–155 °C; ^1^H NMR (DMSO, 600 MHz), δ (ppm): 3.32–3.34 (t, *J* = 2.4 Hz, 1H, CH), 4.21–4.24 (d, *J* = 2.4 Hz, 2H, CH_2_), 6.70–6.98 (m, 2H, H_arom_), 7.01–7.08 (m, 2H, H_arom_), 7.17–7.22 (dd, *J* = 7.8 Hz, *J* = 2.4 Hz, 1H, H_arom_), 7.68–7.74 (dd, *J* = 9 Hz, *J* = 4.2 Hz, 1H, H_arom_), 7.90–7.94 (d, *J* = 8.4 Hz, 1H, H_arom_), 8.93–8.97 (dd, *J* = 4.2 Hz, *J* = 1.2 Hz, 1H, H_arom_), 9.06–9.09 (dd, *J* = 8.4 Hz, *J* = 1.2 Hz, 1H, H_arom_), 9.70 (s, 1H, OH), 10.83 (s, 1H, NH); ^13^C NMR (DMSO, 150.9 MHz), δ (ppm): 55.75 (CH_2_), 78.60 (CH), 78.77 (CCH),109.63, 113.41, 121.55, 123.43, 125.19, 125.35, 125.97, 126.02, 126.86, 132.13, 134.09, 138.74, 149.15, 150.85, 158.55; ESI-HRMS Calcd. for C_18_H_15_N_2_O_4_S ([M + H]^+^) = 355.0752, found: 355.0749.

#### 3.2.3. Synthesis of 8-Methoxyquinoline (**4**)

A solution of 1.450 g (10 mmol) of 8-hydroxyquinoline was added dropwise to a suspension of 0.264 g (11 mmol) of sodium hydride (60% suspension in mineral oil) in 20 mL of anhydrous DMF with stirring. After 1 h, 1.562 g (11 mmol) of methyl iodide was added dropwise to the reaction mixture. The mixture was stirred at room temperature for 5 h. The mixture was poured into 150 mL of water and extracted with chloroform (3 × 15 mL). The combined extracts were dried over anhydrous sodium sulfate. After evaporation of the solvent in vacuo, the crude products were purified by silica column, using a chloroform/ethanol (*v*/*v*) 10:1 mixture as the eluent.

*8-Methoxyquinoline* (**4**): yield: 87%; oil; ^1^H NMR (DMSO, 600 MHz), δ (ppm): 3.96 (s, 3H, CH_3_), 7.14–7.18 (dd, *J* = 6.6 Hz, *J* = 2.4 Hz, 1H, H_arom_), 7.46–7.9 (m, 2H, H_arom_), 7.49–7.54 (dd, *J* = 8.4 Hz, *J* = 4.2 Hz, 1H, H_arom_), 8.27–8.30 (dd, *J* = 8.4 Hz, *J* = 1.8 Hz, 1H, H_arom_), 8.83–8.86 (dd, *J* = 4.2 Hz, *J* = 1.8 Hz, 1H, H_arom_); ^13^C NMR (DMSO, 150.9 MHz), δ (ppm): 56.04 (CH_3_), 110.74, 119.92, 122.30, 127.27, 129.43, 136.18, 140.12, 149.37, 155.65; ESI-HRMS Calcd. for C_10_H_9_NO_2_SCl ([M + H]^+^) = 160.0762, found: 160.0765.

#### 3.2.4. Synthesis of 8-Methoxyquinoline-5-Sulfochloride (**5**)

A flask containing 1.590 g (10 mmol) of 8-methoxyquinoline (**4**) was placed in an ice bath and 5.850 g (50 mmol) of chlorosulfonic acid was added dropwise. The resulting reaction mixture was stirred at room temperature for 24 h. The reaction mixture was poured onto 150 g of ice and extracted with ethylene chloride (3 × 150 mL). The combined extracts were dried over anhydrous sodium sulfate. After evaporating the solvent in vacuo, the obtained product was stored under argon at a temperature of 4–8 °C.

*8-Methoxyquinoline-5-sulfonyl chloride* (**5**): yield: 81%; ^1^H NMR (DMSO, 600 MHz), δ (ppm): 4.16 (s, 3H, CH_3_), 7.58–7.61 (d, *J* = 8.1 Hz, 1H, H_arom_), 8.16–8.19 (d, *J* = 8.1 Hz, 1H, H_arom_), 8.21–5.25 (dd, *J* = 9 Hz, *J* = 5.4 Hz, 1H, H_arom_), 9.16–9.18 (dd, *J* = 5.4 Hz, *J* = 1.8 Hz, 1H, H_arom_), 9.83–9.87 (dd, *J* = 9 Hz, *J* = 1.8 Hz, 1H, H_arom_); ^13^C NMR (DMSO, 150.9 MHz), δ (ppm): 57 (CH_3_), 111.66, 123.42, 126.37, 128.40, 129.50, 137.16, 144.75, 146.51, 150.26; ESI-HRMS Calcd. for C_10_H_9_NO_3_S^35^Cl ([M + H]^+^) = 257.9991 found: 257.9996, Calcd. for C_10_H_9_NO_3_S^37^Cl ([M + H]^+^) = 259.9962 found: 259.9967.

#### 3.2.5. Synthesis of 8-Methoxyquinoline-5-Sulfonamides (**6**)

To a suspension of 1.290 g (5 mmol) of sulfonyl chloride (**5**) in 15 mL of anhydrous acetonitrile, 10 mmol of the appropriate amine and 0.3 mL of triethylamine were added. The resulting reaction mixture was stirred at room temperature for 5 h. The mixture was poured into 100 mL of water and extracted with chloroform (3 × 20 mL). The combined extracts were dried over anhydrous sodium sulfate. After evaporation of the solvent in vacuo, the crude products were purified by silica column, using a chloroform/ethanol (*v*/*v*) 10:1 mixture as the eluent.

*8-Methoxy-N-(prop-2-yn-1-yl)quinoline-5-sulfonamide* (**6a**): yield: 76%; m.p. = 161–163 °C; ^1^H NMR (DMSO, 600 MHz), δ (ppm): 2.81–2.84 (t, *J* = 2.4 Hz, 1H, CH), 3.68–3.70 (d, *J* = 2.4 Hz, 2H, CH_2_), 4.05 (s, 3H, CH_3_), 7.28–7.31 (d, *J* = 9 Hz, 1H, H_arom_), 7.72–7.76 (dd, *J* = 9 Hz, *J* = 4.2 Hz, 1H, H_arom_), 8.15–8.18 (d, *J* = 9 Hz, 1H, H_arom_), 8.40 (s, m, 1H, NH), 8.92–8.95 (dd, *J* = 9 Hz, *J* = 1.5 Hz, 1H, H_arom_), 8.95–8.97 (dd, *J* = 4.2 Hz, *J* = 1.5 Hz, 1H, H_arom_); ^13^C NMR (DMSO, 150.9 MHz), δ (ppm): 31.99 (CH_2_), 56.68 (CH_3_), 74, 64 (CH), 79.84 (CCH),106.73, 123.43, 125.13, 126.87, 131.51, 133.45, 140.06, 14393, 159.55; ESI-HRMS Calcd. for C_13_H_13_N_2_O_2_S: ([M = H]^+^) = 277.0646, found: 277.0640.

*8-Methoxy-N-(1,1-dimethyloprop-2-yn-1-yl)quinoline-5-sulfonamide (***6b**): yield: 65%; m.p. = 217–219 °C; ^1^H NMR (DMSO, 600 MHz), δ (ppm): 1.38 (s, 6H, CH_3_), 2.73 (s, 1H, CH), 4.04 (s, 3H, OCH_3_), 7.25–7.29 (d, *J* = 8.4 Hz, 1H, H_arom_), 7.68–7.74 (dd, *J* = 8.4 Hz, *J* = 3.6 Hz, 1H, H_arom_), 8.14–8.22 (m, 2H, H_arom_), 8.93–8.96 (m, 1H, H_arom_), 8.96–9.02 (m, 1H, H_arom_); ^13^C NMR (DMSO, 150.9 MHz), δ (ppm): 30.87 (CCH_3_), 49.09 (CCH_3_), 56.60 (OCH_3_), 73.29 (CCH), 86.35 (CCH), 106.58, 123.22, 125.38. 128.75, 131.62, 133.54, 139.88, 149.76, 159.35; ESIHRMS Calcd. for C_15_H_17_N_2_O_2_S, ([M + H]^+^) = 305.0959, found: 305.0956.

*8-Methoxy-N-methyl-N-(prop-2-yn-1-yl)quinoline-5-sulfonamide* (**6c**): yield: m.p. = 112–114 °C; 74%; ^1^H NMR (CDCl_3_, 600 MHz), δ (ppm): 1.97–2.02 (t, *J* = 2.4 Hz, 1H, CH), 2.79 (s, 3H, NCH_3_), 3.98–4.02 (d, *J* = 2.4 Hz, 2H, CH_2_), 4.10 (s, 3H, OCH_3_), 7.01–7.06 (d, *J* = 8.4 Hz, 1H, H_arom_), 7.51–7.57 (dd, *J* = 8.4 Hz, *J* = 4.2 Hz, 1H, H_arom_), 8.17–8.22 (d, *J* = 8.4 Hz, 1H, H_arom_), 8.90–8.99 (m, 2H, H_arom_); ^13^C NMR (CDCl_3_, 150.9 MHz), δ (ppm): 34.07 (NCH_3_), 39.01 (CH_2_), 56.60 (OCH_3_), 73.93 (CH_2_C), 76.61 (CCH), 105.61, 123.10, 124.22, 125.89, 132.59, 133.74, 139.95, 149.71, 159.68; ESI-HRMS Calcd. for C_14_H_15_N_2_O_2_S, ([M + H]^+^) = 291.0803 found: 291.0804.

*8-Methoxy-N-[4-(prop-2-yn-1-yloxy)phenyl]quinoline-5-sulfonamide* (**6d**): yield: 70%; m.p. = 217–219 °C; ^1^H NMR (DMSO, 600 MHz), δ (ppm): 3.50–3.53 (t, *J* = 2.4 Hz, 1H, CH), 4.01 (s, 3H, CH_3_), 4.64–4.66 (d, *J* = 2.4 Hz, 2H, CH_2_), 6.75–6.78 (d, *J* = 9 Hz, 2H, H_arom_), 6.85–6.89 (d, *J* = 9 Hz, 2H, H_arom_), 7.21–7.24 (d, *J* = 8.4 Hz, 1H, H_arom_), 7.71–7.75 (dd, *J* = 9 Hz, *J* = 4.2 Hz, 1H, Harom), 8.08–8.12 (d, *J* = 8.4 Hz, 2H, H_arom_), 8.94–8.97 (dd, *J* = 4.2 Hz, *J* = 1.8 Hz, 1H, H_arom_), 8.98–9.01 (dd, *J* = 9 Hz, *J* = 1.8 Hz, 1H, H_arom_), 10.24 (s, 1H, NH); ^13^C NMR (DMSO, 150.9 MHz), δ (ppm): 55.95 (CH_2_), 56.87 (OCH_3_), 78.65 (CH_2_C), 79.59 (CCH), 106.78, 115.39, 115.79, 120.86, 123.02, 123.59, 124.93, 125.82, 131.02, 132.30, 133.04, 139.81, 150.11, 154.68, 159.62; ESI-HRMS Calcd. for C_19_H_17_N_2_O_4_S, ([M + H]^+^) = 369.0909, found: 369.0906.

*8-Methoxy-N-[3-(prop-2-yn-1-yloxy)phenyl]quinoline-5-sulfonamide (***6e**): yield: 67%; m.p. = 190–192 °C; ^1^H NMR (DMSO, 600 MHz), δ (ppm): 3.53–3.57 (t, *J* = 2.4 Hz, 1H, CH), 4.02 (s, 3H, CH_3_), 4.65–4.67 (d, *J* = 2.4 Hz, 1H, CH_2_), 6.55–6.62 (m, 2H, H_arom_), 6.66–6.68 (m, 1H, H_arom_), 7.26–7.29 (d, *J* = 8.4 Hz, H, H^arom^), 7.74–779 (dd, *J* = 8.4 Hz, *J* = 4.2 Hz, 1H, H_arom_), 8.25–8.28 (d, *J* = 8.4 Hz, H, H_arom_), 8.93–8.97 (dd, *J* = 4.2 Hz, *J* = 1.8 Hz, 1H, H_arom_), 9.01–9.05 (dd, *J* = 9 Hz, *J* = 1.8 Hz, 1H, H_arom_); ^13^C NMR (DMSO, 150.9 MHz), δ (ppm): 55.80 (CH_2_), 565.73 (OCH_3_), 78.81 (CH_2_C), 79.44 (CCH), 106.13, 106.81, 110.00, 112.21, 123.74, 124.83, 125.52, 130.44, 132.68, 123.84, 139.15, 139.85, 150.18, 158.11, 159.83; ESI-HRMS Calcd. for C_19_H_17_N_2_O_4_S, ([M + H]^+^) = 369.0909, found: 369.0882.

*8-Methoxy-N-[2-(prop-2-yn-1-yloxy)phenyl]quinoline-5-sulfonamide* (**6f**): yield: 60%; m.p. = 187–189 °C; ^1^H NMR (DMSO, 600 MHz), δ (ppm): 3.31–3.34 (t, *J* = 2.4 Hz, 1H, CH), 4.00 (s, 3H, CH_3_), 4.17–4.19 (d, *J* = 2.4 Hz, 1H, CH_2_), 6.82–6.91 (m, 2H, H_arom_), 7.00–7.09 (m, 1H, H_arom_), 7.14–7.17 (d, *J* = 8.4 Hz, H, H_arom_), 7.15–7.24 (dd, *J* = 7.8 Hz, *J* = 1.8 Hz, 1H, H_arom_), 7.67–7.71 (dd, *J* = 8.4 Hz, *J* = 4.2 Hz, 1H, H_arom_), 7.99–8.00 (d, *J* = 8.4 Hz, H, H_arom_), 8.92–8.95 (dd, *J* = 3.6 Hz, *J* = 1.2 Hz, 1H, H_arom_), 9.04–9.09 (dd, *J* = 8.4 Hz, *J* = 1.8 Hz, 1H, H_arom_), 9.79 (s, 1H, NH); ^13^C NMR (DMSO, 150.9 MHz), δ (ppm): 55.67 (CH_2_), 56.59 (OCH_3_), 78.62 (CH_2_C), 78.64 (CCH), 106.44, 113.39, 121.55, 123.22, 125.22, 125.79, 126.35, 126.97, 127.05, 131.43, 133.68, 138.93, 149.88, 150.98, 159.56; ESI-HRMS Calcd. for C_19_H_17_N_2_O_4_S, ([M + H]^+^) = 369.0909, found: 369.0933.

#### 3.2.6. Synthesis of 1,2,3-Triazole Derivatives of 8-Methoxyquinoline-5-Sulfonamide (7)

##### Procedure A: Preparation of Derivative **7a**

To 5 mL of anhydrous DMF, 0.182 g (1.5 mmol) of allyl bromide and 0.098 g (1.5 mmol) of sodium azide were added, and the resulting suspension was stirred at room temperature for 24 h. Then, 0.276 g (1 mmol) of the 8-methoxyquinoline-5-sulfonamide (**6a**) was added. A solution of 40 mg of sodium ascorbate in 1 mL of distilled water and a solution of 25 mg of copper sulfate pentahydrate in 1 mL of distilled water were prepared. The resulting aqueous solutions were mixed and added to the reaction mixture. The whole mixture was stirred at room temperature for 24 h. The reaction mixture was then poured into 50 mL of water and extracted with chloroform (4 × 20 mL). The combined extracts were dried over anhydrous sodium sulfate. After evaporation of the solvent in vacuo, the crude products were purified by silica column, using a chloroform/ethanol (*v*/*v*) 10:1 mixture as the eluent.

##### Procedure B: Preparation of Derivatives **7b**, **c**, and **7e**–**h**

To 10 mL of anhydrous DMF, 1.5 mmol of the appropriate azide: benzyl azide (3 mL 0.5 M solution in dichloromethane) or (phenylthio)methyl azide 0.248 g (1.5 mmol) and 1 mmol of the appropriate 8-methoxyquinoline-5-sulfonamide (**6a**–**f**) were added. A solution of 40 mg of sodium ascorbate in 1 mL of distilled water and a solution of 25 mg of copper sulfate pentahydrate in 1 mL of distilled water were prepared. The resulting aqueous solutions were mixed and added to the reaction mixture. The whole mixture was stirred at room temperature for 24 h. The reaction mixture was then poured into 50 mL of water and extracted with chloroform (4 × 20 mL). The combined extracts were dried over anhydrous sodium sulfate. After evaporation of the solvent in a vacuum evaporator, the crude products obtained were purified by silica column chromatography, using a chloroform/ethanol (*v*/*v*) 10:1 mixture as the eluent.

##### Procedure C: Preparation of Derivative **7d**

An amount of 10 mL of anhydrous DMF, 1.5 mmol of 4-chlorophenyl azide (3 mL of a 0.5 M solution in *tert-*butyl methyl ether), and 0.276 g (1 mmol) of the 8-methoxyquinoline-5-sulfonamide (**6a**) were placed in a microwave reactor tube. A solution of 40 mg of sodium ascorbate in 1 mL of distilled water and a solution of 25 mg of copper sulfate pentahydrate in 1 mL of distilled water were prepared. The resulting aqueous solutions were mixed and added to the reaction mixture. The reaction was carried out in a microwave reactor at 100 °C for 45 min. The solution was then cooled to room temperature and poured into 50 mL of water. The whole mixture was extracted with chloroform (4 × 20 mL). The combined extracts were dried over anhydrous sodium sulfate. After evaporation of the solvent in vacuo, the crude products were purified by silica column chromatography. Chloroform was used as an eluent, followed by chloroform/ethanol (*v*/*v*) 10:1.

*N-[(1-Allyl-1H-1,2,3-triazol-4-yl)methyl]-8-methoxyquinoline-5-sulfonamide* (**7a**): Procedure A. yield: 65%; m.p. = 159–161 °C; ^1^H NMR (CDCl_3_, 600 MHz), δ (ppm): 4.05 (s, 3H, CH_3_), 4,19 (s, 2H, CH_2_), 4.67–4.71 (d, *J* = 6 Hz, 2H, CH_2_CH=CH_2_), 5.05–5.11 (d, *J* = 16.8 Hz, 1H, CH_2_CH=CH_2_), 517–5.19 (d, *J* = 10.2 Hz, 1H, CH_2_CH=CH_2_), 5.70–7.77 (m, 1H, CH_2_CH=CH_2_), 6.80–6.90 (m, 1H, H_arom_), 6.90–7.03 (m, 1H, H_arom_), 7.46 (s, 1H, CH), 8.16–8.22 (m, 1H, H_arom_), 8.85–9.0 (m, 2H, H_arom_); ^13^C NMR (CDCl_3_, 150.9 MHz), δ (ppm): 30.45 (NHCH_2_), 51.74 (CH_2_), 55.66 (CH_3_), 104.70, 119.37, 122.32, 124.20, 125.25, 129.78, 130.74, 133.00, 138.47, 148.48, 158.13; ESI-HRMS Calcd. for C_16_H_18_N_5_O_3_S, ([M + H]^+^) = 360.1130, found: 360.1121.

*N-[(1-Benzyl-1H-1,2,3-triazol-4-yl)methyl]-8-methoxyquinoline-5-sulfonamide* (**7b**): Procedure B. yield: 68%; m.p. = 170–172 °C; ^1^H NMR (DMSO, 600 MHz), δ (ppm): 4.00–4.11 (m, 5H, NHCH_2_, CH_3_), 5.42 (s, 2H, NCH_2_), 7.15–7.22 (m, 2H, H_arom_), 7.22–7.28 (m, 1H, H_arom_), 7.28–7.40 (m, 3H, H_arom_), 7.65–7.78 (m, 2H, CH, H_arom_), 8.10–8.15 (m, 1H, H_arom_), 7.40–7.44 (m, 1H, H_arom_), 8.89–9.00 (m, 2H, H_arom_); ^13^C NMR (DMSO, 150.9 MHz), δ (ppm): 38.14 (NHCH_2_), 53.05 (CH_2_), 56.69 (CH_3_), 106.67, 123.49, 123.67, 124.86, 126.96, 128.33, 128.56, 129.18, 131.25, 133.36. 136.30, 140.07, 144.10, 149.95, 159.41; ESI-HRMS Calcd. for C_20_H_20_N_5_O_3_S, ([M + H]^+^) = 410.1286, found: 410.1285.

*N-{[1-(4-Fluorobenzyl)-1H-1,2,3-triazol-4-yl]methyl}-8-methoxyquinoline-5-sulfonamide* (**7c**): Procedure B. yield: 64%; m.p. = 194–196 °C; ^1^H NMR (DMSO, 600 MHz), δ (ppm): 4.02–4.04 (d, *J* = 6 Hz, 2H, NHCH_2_,), 4.05 (s, 3H, CH_3_), 5.42 (s, 2H, NCH_2_), 7.16–7.24 (m, 2H, H_arom_), 7.24–7.27 (m, 3H, H_arom_), 7.67–7.71 (m, 1H, H_arom_), 7.73 (s, 1H, CH), 8.11–8.14 (m, 1H, H_arom_), 8.39–8.43 (m, 1H, H_arom_), 8.90–8.93 (m, 1H, H_arom_), 8.93–8.96 (m, 1H, H_arom_); ^13^C NMR (DMSO, 150.9 MHz), δ (ppm): 38.13 (NHCH_2_), 52.23 (CH_2_), 56.67 (CH_3_), 106.69, 116.02 (d, *J_C-F_* = 90 Hz), 123.49 (d, *J_C-F_* = 90 Hz), 124.85, 126.93, 130.63 (d, *J_C-F_* = 36 Hz), 131,23, 152.57 (d, *J_C-F_* = 12 Hz), 133.39, 140.01, 144.14, 149.97, 159.37, 161.49, 163.11; ESI-HRMS Calcd. for C_20_H_19_FN_5_O_3_S, ([M + H]^+^) = 428.1192, found: 428.1184.

*N-{[1-(4-Chlorophenyl)-1H-1,2,3-triazol-4-yl]methyl}-8-methoxyquinoline-5-sulfonamide* (**7d**): Procedure C. yield: 57%; m.p. = 224–226 °C; ^1^H NMR (DMSO, 600 MHz), δ (ppm): 3.93 (s, 3H, CH_3_), 4.12–4.17 (d, *J* = 5.4 Hz, 2H, NHCH_2_), 7.16–7.20 (m, 1H, H_arom_), 7.40–7.45 (m, 2H, H_arom_), 7.65–7.71 (m, 3H, H_arom_), 8.10–8.14 (m, 1H, H_arom_), 8.24 (s, 1H, CH), 8.51–8.57 (m, 1H, H_arom_), 8.87–8.94 (m, 2H, H_arom_); ^13^C NMR (DMSO, 150.9 MHz), δ (ppm): 37.92 (NHCH_2_), 56.50 (CH_3_), 106.51, 117.04, 117.19, 122.09, 122.58, 122.64, 126.97, 131.46, 133.41, 139.86, 144.41, 149.84, 159.34, 161.21, 162.83; ESI-HRMS Calcd. for C_19_H_17_N_5_O_3_S^35^Cl, ([M + H]^+^) = 430.0740, found: 430.0729; Calcd. for C_19_H_17_N_5_O_3_S^37^Cl, ([M + H]^+^) = 432.0735, found: 432.0704.

*N-({1-[(Phenylthio)methyl]-1H-1,2,3-triazol-4-yl}methyl)-8-methoxyquinoline-5-sulfonamide* (**7e**): Procedure B. yield: 65%; m.p. = 205–207 °C; ^1^H NMR (CDCl_3_, 600 MHz), δ (ppm): 4.14 (s, 3H, CH_3_), 4.20 (s, 2H, NHCH_2_), 5.46 (s, 2H, NCH_2_), 7.03 (bs, 1H, NH), 7.07–7.11 (m, 1H, H_arom_), 7.23–7.28 (m, 5H, H_arom_), 7.46 (s, 1H, CH), 7.60–7.68 (m, 1H, H_arom_), 8.26–8.31 (m, 1H, H_arom_), 9.02–9.09 (m, 1H, H_arom_), 9.15–9.27 (m, 1H, H_arom_); ^13^C NMR (CDCl_3_, 150.9 MHz), δ (ppm): 37.39 (NHCH_2_), 52.89 (CH_2_), 55.60 (CH_3_), 104.38, 122.28, 123.06, 124.21, 124.93, 127.69, 128.49, 128.65, 130.65, 130.74, 131.02, 132.22, 139.14, 148.84, 158.62; ESI-HRMS Calcd. for C_20_H_20_N_5_O_3_S_2_, ([M + H]^+^) = 442.1007, found: 442.0991.

*N-[2-(1-Benzyl-1H-1,2,3-triazol-4-yl)propan-2-yl]-8-methoxyquinoline-5-sulfonamide* (**7f**): Procedure B. yield: 60%; m.p. = 153–155 °C; ^1^H NMR (CDCl_3_, 600 MHz), δ (ppm): 1.60 (s, 6H, CH_3_), 4.19 (s, 3H, OCH_3_), 5.28 (s, 2H, CH_2_), 6.96–7.00 (m, 2H, H_arom_), 7.16–7.19 (m, 1H, H_arom_), 7.35–7.38 (m, 1H, H_arom_), 8.14–8.18 (m, 1H, H_arom_), 8.94–8.98 (m, 1H, H_arom_), 9.05–9.09 (m, 1H, H_arom_); ^13^C NMR (DMSO, 150.9 MHz), δ (ppm): 29.12 (CCH_3_), 52.9 (CCH_3_), 53.20 (CH_2_), 56.61 (CH_3_), 106.39, 122.40, 123.11, 124.78, 128.49, 128.57, 129.17, 129.34, 130.52, 133.55, 136.32, 139.79, 149.72, 151.69, 158.97; ESI-HRMS Calcd. for C_22_H_24_N_5_O_3_S, ([M + H]^+^) = 438.1599, found: 438.1596.

*N-Methyl-N-[(1-benzyl-1H-1,2,3-triazol-4-yl)methyl]-8-methoxyquinoline-5-sulfonamide* (**7g**): Procedure B. yield: 68%; m.p. = 214–216 °C; ^1^H NMR (CDCl_3_, 600 MHz), δ (ppm): 2.78 (s, 3H, NCH_3_), 4.19 (s, 3H OCH_3_), 4.49 (s, 2H, CH_3_NCH_2_), 5.49 (s, 2H, NCH_2_), 7.08–7.11 (d, *J* = 9 Hz), 1H, H_arom_), 7.24–7.27 (m, 2H, H_arom_), 7.38–7.41 (m, 3H, H_arom_), 7.45 (s, 1H, CH), 7.55–7.59 (dd, *J* = 8.4 Hz, *J* = 4.2 Hz, 1H, H_arom_), 8.23–8.26 (d, *J* = 8.4 Hz), 1H, H_arom_), 9.03–9.05 (m, 1H, H_arom_); ^13^C NMR (CDCl_3_, 150.9 MHz), δ (ppm): 33.32, 43.66, 53.26, 55.80, 105.29, 121.86, 122.25, 123.96, 124.79, 127.06, 127.86, 128.17, 131.61, 133.33, 134.42, 137.29, 142.32, 147.91, 157.72; ESI-HRMS Calcd. for C_21_H_22_N_5_O_3_S, ([M + H]^+^) = 424.1443, found: 424.1448.

*N-{4-[(1-Benzyl-1H-1,2,3-triazol-4-yl)methoxy]phenyl}-8-methoxyquinoline-5-sulfonamide* (**7h**): Procedure B. yield: 62%; m.p. = 139–141 °C; ^1^H NMR (DMSO, 600 MHz), δ (ppm): 4.01 (s, 3H, CH_3_), 4.98 (s, 2H, NHCH_2_), 5.58 (s, 2H, NCH_2_), 6.78–7.83 (m, 2H, H_arom_), 6.83–6.88 (m, 2H, H_arom_), 7.20–7.28 (m. 1H, H_arom_), 7.28–7.33 (m, 2H, H_arom_), 7.33–7.38 (m, 3H, H_arom_), 7.70–7.78 (m, 1H, H_arom_), 8.07–8.10 (d, *J* = 8.4 Hz, 1H, H_arom_), 8.21 (s, 1H, CH), 8.90–9.03 (m, 2H, NH, H_arom_), 10.18–10.23 (m, 1H, H_arom_); ^13^C NMR (DMSO, 150.9 MHz), δ (ppm): 53.26 (CH_2_), 56.66 (CH_3_), 61.58 (CH_2_), 106.76, 115.60, 123.18, 123.63, 125.10, 125.83, 128.38, 128.61, 128.92, 129.22, 130.64, 132.31, 133.05, 136.45, 139.85, 143.31, 150.06, 155.56, 159.65: ESI-HRMS Calcd. for C_26_H_24_N_5_O_4_S, ([M + H]^+^) = 502.1549, found: 502.1542.

### 3.3. Biological Evaluation

#### 3.3.1. Cell Culture

Compounds were evaluated for their antiproliferative activity using three cultured cell lines: C-32 (human amelanotic melanoma, ATCC, Manassas, VA, USA), A549 (human lung adenocarcinoma, ATCC, Manassas, VA, USA), MDA-MB-231 (human breast adenocarcinoma derived from metastatic site: pleural effusion, ATCC, Manassas, VA, USA), together with HFF-1 (normal human dermal fibroblasts, ATCC, Manassas, VA, USA). The cultured cells were kept at 37 °C and 5% CO_2_.

#### 3.3.2. Effect of Compounds on Number and Viability of Cells

Cells were seeded in 96-well plates at 5000/well in 100 μL of medium. After 24 h of incubation, the medium was replaced with a new one with the addition of the test substance. After 72 h, the following tests were performed: with crystal violet (determination of the number of adherent cells in the culture), the WST-1 test (determination of the relative number of living cells and their metabolic activity), and the LDH test (determination of the relative number of dead cells in cultures—cytotoxicity of the tested substance).

*Crystal violet test:* The crystal violet (Sigma-Aldrich, Darmstadt, Germany) test determines the relative number of cells based on the amount of dye bound to the DNA of cells in the culture. The absorbance measurement was performed at a wavelength of λ = 540 nm (reference wavelength λ = 690 nm).

*WST-1 test:* The WST-1 test (Roche Diagnostics, Mannheim, Germany) allows one to determine the relative number of cells in cultures. Absorbance is directly proportional to the number of cells and their metabolic activity. The absorbance measurement is performed at λ = 450 nm (reference wave 600 nm).

*LDH test:* The LDH test (Roche Diagnostics) involves measuring lactate dehydrogenase released by dead cells into the medium. It is a measure of the cytotoxic effect of an agent on cells in culture. The absorbance measurement was performed at a wavelength of λ = 490 nm (reference wave λ = 600 nm). A UVM340 microplate reader (Asys-Hitech GmbH UVM340 (Eugendorf, Austria)) was used for absorbance measurements.

#### 3.3.3. Wound Healing Assay

The wound healing assay is a standard in vitro technique for examining collective cell migration in two dimensions. The test involves scratching the bottom of a vessel (wells of a culture plate) with a pipette tip and observing over time how quickly cells move to occupy a free surface under specific culture conditions. In the presented work, the observation was carried out after 72 h of culture. At t = 0, a scratch was made, the medium was replaced with fresh one with the addition of the test compound, and then the cultures were incubated for 72 h. The observation was carried out using an OLYMPUS IX 50 microscope (Tokyo, Japan) with a KERN camera (KERN & SOHN GmbH, Balingen, Germany), and an image analysis system with KERN software (BalanceConnection SCD-4.0 Pro) was used to analyze the observed changes.

#### 3.3.4. Transcriptional Activity of H3, BCL-2, BAX, P21, and P53 Genes

The transcriptional activity of the following genes was assessed: H3 (a gene encoding histone H3, a proliferation marker), BCL-2 and BAX (genes encoding BCL-2 and BAX proteins, respectively; both are mitochondrial proteins associated with apoptosis), and P21 and P53 (cell cycle regulators). Activity was assessed by RT-QPCR using the CFX96 Touch Real-Time PCR Detection System (BIO-RAD Hercules, CA, USA) and a SensiFAST^TM^ SYBR^R^ No-ROX One-Step Kit (Meridian Bioscience, Memphis, TN, USA). Cultured cells were exposed (for 24 h) to the tested compounds (0.5 µg/mL). RNA was extracted using Quick-RNA™ MiniPrep kit columns (Zymo Research, Irvine, CA, USA). The extracted RNA was assessed qualitatively and quantitatively. The integrity of total RNA was checked by electrophoresis (1.2% agarose gel, EtBr), and the amount and purity of total RNA in the extracts was determined spectrophotometrically (HP8452A apparatus, Hewlett Packard, Waldbronn, Germany).

#### 3.3.5. In Vitro Antibacterial Evaluation

The in vitro antibacterial activity of the synthesized compounds was evaluated against representatives of multidrug-resistant bacteria, three clinical isolates of methicillin-resistant *S. aureus*: clinical isolates of human origin, MRSA SA 3202, and MRSA SA 630 (National Institute of Public Health, Prague, Czech Republic), carrying the *mecA* gene [76,77]. These two clinical isolates were classified as vancomycin-susceptible (but with a higher MIC of vancomycin equal to 2 μg/mL (VA2-MRSA) [76]. Vancomycin- and methicillin-susceptible *S. aureus* ATCC 29213 and vancomycin-susceptible *Enterococcus faecalis* ATCC 29212, obtained from the American Type Culture Collection, were used as the reference and quality control strains. Three *vanA* gene-carrying vancomycin-resistant isolates of *E. faecalis* (VRE 342B, VRE 368, VRE 725B) (Department of Infectious Diseases and Microbiology, Faculty of Veterinary Medicine, University of Veterinary Sciences Brno, Czech Republic) were provided by Oravcova et al. [77].

The minimum inhibitory concentrations (MICs) were evaluated by the microtitration broth method according to the CLSI, with some modifications [83,84]. The compounds were dissolved in DMSO (Sigma, St. Louis, MO, USA) to obtain a concentration of 10 µg/mL and diluted in a microtitration plate in an appropriate medium, i.e., Cation-Adjusted Mueller–Hinton Broth (CaMH, Oxoid, Basingstoke, UK) for staphylococci, and Brain Heart Infusion Broth (BHI, Oxoid) for enterococci to reach the final concentration of 256–0.125 µg/mL. Microtiter plates were inoculated with test microorganisms so that the final concentration of bacterial cells was 10^5^. Ampicillin, oxacillin, and ciprofloxacin (Sigma) were used as reference drugs. A drug-free control and a sterility control were included. The plates were incubated for 24 h at 37 °C for staphylococci and enterococci. After static incubation in the darkness in an aerobic atmosphere, the MIC was visually evaluated as the lowest concentration of the tested compound, which completely inhibited the growth of the microorganism. The experiments were repeated three times. The results are summarized in Table 3.

#### 3.3.6. Determination of Minimum Bactericidal Concentrations

For the above-mentioned strains/isolates, the agar aliquot subculture method was used as a test for bactericidal agents [85,86]. After the MIC value determination, the inoculum was transferred to CaMH (Oxoid) for staphylococci, and BHI (Oxoid) for enterococci medium using a multipoint inoculator. The plates were incubated in a thermostat at 37 °C for 24 h. The lowest concentration of test compound at which ≤5 colonies were obtained was then evaluated as MBC, corresponding to a 99.9% decrease in CFU relative to the original inoculum.

#### 3.3.7. MTT Assay

Compounds were prepared as previously stated and diluted in CaMH broth for *S. aureus* to achieve the desired final concentrations. *S. aureus* bacterial suspension in sterile distilled water at 0.5 McFarland was diluted 1:3. Inocula were added to each well by multi-inoculator. Diluted mycobacteria in broth free from inhibiting compounds were used as the growth control. All compounds were prepared in duplicate. Plates were incubated at 37 °C for 24 h for *S. aureus*. After the incubation period, 10% well volume of MTT (3-(4,5-dimethylthiazol-2-yl)-2,5-diphenyltetrazolium bromide) reagent (Sigma) was mixed into each well and incubated at 37 °C 1 h for *S. aureus*. Then, 100 µL of 17% sodium dodecyl sulphate in 40% dimethylformamide was added to each well. The plates were read at 570 nm. The absorbance readings from the cells grown in the presence of the tested compounds were compared with uninhibited cell growth to determine the relative percent inhibition. The percent inhibition was determined through MTT assay. The percent viability is calculated through the comparison of a measured value and that of the uninhibited control: % viability = OD_570E_/OD_570P_ × 100, where OD_570E_ is the reading from the compound-exposed cells, while OD_570P_ is the reading from the uninhibited cells (positive control). Cytotoxic potential is determined by a percent viability of <70% [81,82]. The results are summarized in Table 4.

## 4. Conclusions

A series of unique acetylene derivatives of 8-hydroxy and 8-methoxyquinoline-5-sulfonamide **3a**–**f** and **6a**–**f** were prepared by reactions of 8-hydroxy and 8-methoxyquinoline-5-sulfonyl chlorides with acetylene derivatives of amine. A series of new hybrid systems containing quinoline and 1,2,3-triazole systems **7a**–**h** were obtained by the reaction of acetylene derivatives of quinoline-5-sulfonamide **6a**–**d** with organic azides. Biological screening performed on three cancer cell lines C-32, MDA-MB-231, and A549, reference bacterial strains *S. aureus* ATCC 29213 and *E. faecalis* ATCC 29212, and clinical isolates of MRSA and VRE demonstrated the inactivity of 8-methoxy derivatives **6a**–**d** as well as 1,2,3-triazole derivatives **7a**–**h**. In contrast, the acetylenic derivatives of 8-hydroxyquinoline-5-sulfonamide **3a**–**f** were shown to be biologically active. 8-Hydroxy-*N*-methyl-*N*-(prop-2-yn-1-yl)quinoline-5-sulfonamide (**3c**) showed the highest activity against all three cancer lines and MRSA isolates, comparable to that of cisplatin/doxorubicin and oxacillin/ciprofloxacin. In the non-cancer HFF-1 cells, derivative **3c** showed no cytotoxicity up to an IC_50_ of 100 µM. In further tests, compound **3c** decreased the expression of H3, increased the transcriptional activity of cell cycle regulators (P53 and P21 proteins), and altered the expression of BCL-2 and BAX genes in all cancer lines. It can be assumed that both anticancer and antibacterial activities of the discussed 8-HQ derivatives are related to the presence of a free phenolic group in position 8 of the quinoline pharmacophore. Irreversible blockade (formation of methyl ether) results in the loss of biological activity, which was not restored even after the introduction of 1,2,3-triazine onto the 8-methoxyquinoline scaffold. Since 8-HQ are multi-target agents, it is very difficult to draw a conclusion regarding the exact mechanism of action. Thus, this is a challenge for future detailed investigation of the most promising agent **3c**.

## Figures and Tables

**Table 1:** In vitro antiproliferative activity (IC_50_ [µM] ± SD, n = 6) against human cancer cell lines and cell viability of normal human cells of 8-(hydroxyl)quinoline-5-sulfonamides **3a**–**f** and 8-methoxyquinoline-5-sulfonamides **6a**–**f** compared to cisplatin (CPT) and doxorubicin (DOX).

Comp.	IC_50_ [µM]			
	C-32	MDA-MB-231	A549	HFF-1
**3a**	19.8 ±1.1	23.3 ± 1.2	23.1 ± 0.7	˃100
**3b**	22.1 ±1.5	55.8 ± 1.9	17.2 ± 1.5	˃100
**3c**	9.8 ± 0.6	2.2 ± 0.1	13.9 ± 0.6	˃100
**3d**	10.4 ± 0.5	45.3 ± 5.4	12.9 ± 1.1	˃100
**3e**	8.6 ± 0.9	15.2 ± 0.8	11.5 ± 0.7	˃100
**3f**	29.6 ± 2.6	14.7 ± 1.2	19.1 ± 0.8	28.1 ± 2.2
**6a**	˃100	˃100	˃100	–
**6b**	40.6 ± 4.9	˃100	˃100	–
**6c**	70.1 ± 8.4	˃100	˃100	–
**6d**	86.4 ± 3.9	˃100	˃100	–
**6e**	32.2 ± 4.8	˃100	˃100	–
**6f**	27.7 ± 2.2	˃100	˃100	–
**CPT**	10.6 ± 0.5	10.3 ± 0.7	17.3 ± 1.3	26.0 ± 2.0
**DOX**	0.3 ± 0.02	0.6 ± 0.1	1.0 ± 0.1	1.4 ± 0.1

C-32 = human amelanotic melanoma cells; MDA-MB-231 = human breast adenocarcinoma cells; A549 = human lung adenocarcinoma cells; HFF-1 = normal human dermal fibroblasts.

**Table 2:** In vitro antiproliferative activity (IC_50_ [µM] ± SD, n = 6) against human cancer cell lines of 1,2,3-triazole derivatives 8-methoxy-5-sulfonamides **7a**–**h** compared to cisplatin (CPT) and doxorubicin (DOX).

Comp.	IC_50_ [µM]		
	C-32	MDA-MB-231	A549
**7a**	˃100	˃100	˃100
**7b**	˃100	˃100	˃100
**7c**	89.0 ± 3.5	˃100	˃100
**7d**	˃100	˃100	˃100
**7e**	˃100	72.2 ± 3.6	˃100
**7f**	˃100	˃100	˃100
**7g**	42.6 ± 3.4	˃100	˃100
**7h**	˃100	˃100	˃100
**CPT**	10.6 ± 0.5	10.3 ± 0.7	17.3 ± 1.3
**DOX**	0.3 ± 0.02	0.6 ± 0.1	1.0 ± 0.1

C-32 = human amelanotic melanoma cells; MDA-MB-231 = human breast adenocarcinoma cells; A549 = human lung adenocarcinoma cells.

**Table 3:** In vitro antistaphylococcal and anti-enterococcal activities (MIC/MBC [µM]) compared to ampicillin (AMP), oxacillin (OXA), and ciprofloxacin (CPX).

Comp.	MIC/MBC [µM]						
	SA	MRSA1	MRSA2	EF	VRE1	VRE2	VRE3
**3a**	122/**122**	122/**122**	244/**244**	977/>977	977/NT	977/NT	977/NT
**3b**	55.2/**110**	55.2/**110**	110/**110**	883/>883	883/NT	883/NT	883/NT
**3c**	29.0/**29.0**	29.0/**29.0**	29.0/**29.0**	464/>464	464/NT	464/NT	464/NT
**3d**	45.2/**45.2**	45.2/**45.2**	90.4/**90.4**	362/362	362/NT	362/NT	362/NT
**3e**	45.2/**45.2**	45.2/**90.4**	90.4/**90.4**	362/362	362/NT	362/NT	362/NT
**3f**	45.2/**45.2**	45.2/**90.4**	90.4/**90.4**	362/362	362/NT	362/NT	362/NT
**AMP**	5.72/5.72	>45.8/>45.8	>45.8/>45.8	2.81/2.81	11.5/11.5	11.5/11.5	11.5/11.5
**OXA**	1.25/1.25	79.8/79.8	29.7/29.7	–	–	–	–
**CPX**	1.51/3.02	48.3/96.6	193/386	1.51/3.02	1.51/3.02	3.02/3.02	193/386

SA = *Staphylococcus aureus* ATCC 29213; MRSA1, 2 = clinical isolates of methicillin-resistant *S. aureus* SA 3202, SA 630 (National Institute of Public Health, Prague, Czech Republic); EF = *Enterococcus faecalis* ATCC 29213 and vancomycin-resistant enterococci VRE1–3 = VRE 342B, VRE 368, and VRE 725B (Department of Infectious Diseases and Microbiology, Faculty of Veterinary Medicine, University of Veterinary Sciences Brno, Czech Republic); NT = not tested. The real bactericidal values required by the MBC/MIC ≤ 4 rule are in bold.

**Table 4:** Lowest MIC values with at least 70% inhibition of *S. aureus* ATCC 29213 respiratory activity.

Comp.	Conc.	*S. aureus* Respiration Inhibition [%]
**3a**	1× MIC (1× MBC)	92.5
**3b**	2× MIC (2× MBC)	94.4
**3c**	2× MIC (2× MBC)	95.2
**3d**	1× MIC (1× MBC)	73.0
**3e**	1× MIC (1× MBC)	73.7
**3f**	1× MIC (1× MBC)	81.6
**APM**	16× MIC (>16× MBC)	81.9
**CPX**	32× MIC (16× MBC)	95.0

## Data Availability

Data are contained within the article.

## Supplementary Materials

The following supporting information can be downloaded at: https://www.mdpi.com/article/10.3390/molecules29174044/s1, Figure S1: ^1^H NMR spectrum of 8-hydroxyquinoline-5-sulfonyl chloride (**2**) in DMSO; Figure S2: ^13^C NMR spectrum of 8-hydroxyquinoline-5-sulfonyl chloride (**2**) in DMSO; Figure S3: HR-MS spectrum of 8-hydroxyquinoline-5-sulfonyl chloride (**2**); Figure S4: ^1^H NMR spectrum of 8-hydroxy-*N*-(prop-2-yn-1-yl)quinoline-5-sulfonamide (**3a**) in DMSO; Figure S5: ^13^C NMR spectrum of 8-hydroxy-*N*-(prop-2-yn-1-yl)quinoline-5-sulfonamide (**3a**) in DMSO; Figure S6: HR-MS spectrum of 8-hydroxy-*N*-(prop-2-yn-1-yl)quinoline-5-sulfonamide (**3a**); Figure S7: ^1^H NMR spectrum of 8-hydroxy-*N*-(1,1-dimethylprop-2-yn-1-yl)quinoline-5-sulfonamide (**3b**) in DMSO; Figure S8: ^13^C NMR spectrum of 8-hydroxy-*N*-(1,1-dimethylprop-2-yn-1-yl)quinoline- 5-sulfonamide (**3b**) in DMSO; Figure S9: HR-MS spectrum of 8-hydroxy-*N*-(1,1-dimethylprop- 2-yn-1-yl)quinoline-5-sulfonamide (**3b**); Figure S10: ^1^H NMR spectrum of 8-hydroxy-*N*-methyl-*N*-(prop-2-yn-1-yl)quinoline-5-sulfonamide (**3c**) in CD_3_OD; Figure S11: ^13^C NMR spectrum of 8-hydroxy-*N*-methyl-*N*-(prop-2-yn-1-yl)quinoline-5-sulfonamide (**3c**) in CDCl_3_; Figure S12: HR-MS spectrum of 8-hydroxy-*N*-methyl-*N*-(prop-2-yn-1-yl)quinoline-5-sulfonamide (**3c**); Figure S13: ^1^H NMR spectrum of 8-hydroxy-*N*-[4-(prop-2-yn-1-yloxy)phenyl]quinoline- 5-sulfonamide (**3d**) in CDCl_3_; Figure S14: ^13^C NMR spectrum of 8-hydroxy-*N*-[4-(prop-2-yn-1-yloxy)phenyl]quinoline-5-sulfonamide (**3d**) in CDCl_3_; Figure S15: HR-MS spectrum of 8-hydroxy-*N*-[4-(prop-2-yn-1-yloxy)phenyl]quinoline-5-sulfonamide (**3d**); Figure S16: ^1^H NMR spectrum of 8-hydroxy-*N*-[3-(prop-2-yn-1-yloxy)phenyl]quinoline-5-sulfonamide (**3e**) in DMSO; Figure S17: ^13^C NMR spectrum of 8-hydroxy- *N*-[3-(prop-2-yn-1-yloxy)phenyl]quinoline-5-sulfonamide (**3e**) in DMSO; Figure S18: HR-MS spectrum of 8-hydroxy-*N*-[3-(prop-2-yn-1-yloxy)phenyl]quinoline-5-sulfonamide (**3e**); Figure S19: ^1^H NMR spectrum of 8-hydroxy-*N*-[2-(prop-2-yn-1-yloxy)phenyl]quinoline-5-sulfonamide (**3f**) in DMSO; Figure S20: ^13^C NMR spectrum of 8-hydroxy-*N*-[2-(prop-2-yn-1-yloxy)phenyl]quinoline- 5-sulfonamide (**3f**) in DMSO; Figure S21: HR-MS spectrum of 8-hydroxy-*N*-[2-(prop-2-yn-1-yloxy)- phenyl]quinoline-5-sulfonamide (**3f**); Figure S22: ^1^H NMR spectrum of 8-methoxyquinoline (**4**) in DMSO; Figure S23: ^13^C NMR spectrum of 8-methoxyquinoline (**4**) in DMSO; Figure S24: HR-MS spectrum of 8-methoxyquinoline (**4**); Figure S25: ^1^H NMR spectrum of 8-methoxyquinoline-5-sulfochloride (**5**) in DMSO; Figure S26: ^13^C NMR spectrum of 8-methoxyquinoline-5-sulfochloride (**5**) in DMSO; Figure S27: HR-MS spectrum of 8-methoxyquinoline-5-sulfochloride (**5**); Figure S28: ^1^H NMR spectrum of 8-methoxy-*N*-(prop-2-yn-1-yl)quinoline-5-sulfonamide (**6a**) in DMSO; Figure S29: ^13^C NMR spectrum of 8-methoxy-*N*-(prop-2-yn-1-yl)quinoline-5-sulfonamide (**6a**) in DMSO; Figure S30: HR-MS spectrum of 8-methoxy-*N*-(prop-2-yn-1-yl)quinoline-5-sulfonamide (**6a**); Figure S31: ^1^H NMR spectrum of 8-methoxy-*N*-(1,1-dimethyloprop-2-yn-1-yl)quinoline-5-sulfonamide (**6b**) in DMSO; Figure S32: ^13^C NMR spectrum of 8-methoxy-*N*-(1,1-dimethyloprop-2-yn-1-yl)quinoline- 5-sulfonamide (**6b**) in DMSO; Figure S33: HR-MS spectrum of 8-methoxy- *N*-(1,1-dimethyloprop-2-yn-1-yl)quinoline-5-sulfonamide (**6b**); Figure S34: ^1^H NMR spectrum of 8-methoxy-*N*-methyl-*N*-(prop-2-yn-1-yl)quinoline-5-sulfonamide (**6c**) in CDCl_3_; Figure S35: ^13^C NMR spectrum of 8-methoxy-*N*-methyl-*N*-(prop-2-yn-1-yl)quinoline-5-sulfonamide (**6c**) in CDCl_3_; Figure S36: HR-MS spectrum of 8-methoxy-*N*-methyl-*N*-(prop-2-yn-1-yl)quinoline-5-sulfonamide (**6c**); Figure S37: ^1^H NMR spectrum of 8-methoxy-*N*-[4-(prop-2-yn-1-yloxy)phenyl]quinoline- 5-sulfonamide (**6d**) in DMSO; Figure S38: ^13^C NMR spectrum of 8-methoxy-*N*-[4-(prop-2-yn-1-yloxy)phenyl]quinoline-5-sulfonamide (**6d**) in DMSO; Figure S39: HR-MS spectrum of 8-methoxy-*N*-[4-(prop-2-yn-1-yloxy)phenyl]quinoline-5-sulfonamide (**6d**); Figure S40: ^1^H NMR spectrum of 8-methoxy-*N*-[3-(prop-2-yn-1-yloxy)phenyl]quinoline- 5-sulfonamide (**6e**) in DMSO; Figure S41: ^13^C NMR spectrum of 8-methoxy-*N*-[3-(prop-2-yn-1-yloxy)phenyl]quinoline-5-sulfonamide (**6e**) in DMSO; Figure S42: HR-MS spectrum of 8-methoxy-*N*-[3-(prop-2-yn-1-yloxy)phenyl]quinoline-5-sulfonamide (**6e**); Figure S43: ^1^H NMR spectrum of 8-methoxy-*N*-[2-(prop-2-yn-1-yloxy)phenyl]quinoline- 5-sulfonamide (**6f**) in DMSO; Figure S44: ^13^C NMR spectrum of 8-methoxy- *N*-[2-(prop-2-yn-1-yloxy)phenyl]quinoline-5-sulfonamide (**6f**) in DMSO; Figure S45: HR-MS spectrum of 8-methoxy-*N*-[2-(prop-2-yn-1-yloxy)phenyl]quinoline-5-sulfonamide (**6f**); Figure S46: ^1^H NMR spectrum of *N*-[(1-allyl-1*H*-1,2,3-triazol-4-yl)methyl]-8-methoxyquinoline-5-sulfonamide (**7a**) in CDCl_3_; Figure S47: ^13^C NMR spectrum of *N*-[(1-allyl-1*H*-1,2,3-triazol-4-yl)methyl]- 8-methoxyquinoline-5-sulfonamide (**7a**) in CDCl_3_; Figure S48: HR-MS spectrum of *N*-[(1-allyl-1*H*-1,2,3-triazol-4-yl)methyl]-8-methoxyquinoline-5-sulfonamide (**7a**); Figure S49: ^1^H NMR spectrum of *N*-[(1-benzyl-1*H*-1,2,3-triazol-4-yl)methyl]-8-methoxyquinoline-5-sulfonamide (**7b**) in DMSO; Figure S50: ^13^C NMR spectrum of *N*-[(1-benzyl-1*H*-1,2,3-triazol-4-yl)methyl]- 8-methoxyquinoline-5-sulfonamide (**7b**) in DMSO; Figure S51: HR-MS spectrum of *N*-[(1-benzyl-1*H*-1,2,3-triazol-4-yl)methyl]-8-methoxyquinoline-5-sulfonamide (**7b**); Figure S52: ^1^H NMR spectrum of *N*-{[1-(4-fluorobenzyl)-1*H*-1,2,3-triazol-4-yl]methyl}-8-methoxyquinoline- 5-sulfonamide (**7c**) in DMSO; Figure S53: ^13^C NMR spectrum of *N*-{[1-(4-fluorobenzyl)- 1*H*-1,2,3-triazol-4-yl]methyl}-8-methoxyquinoline-5-sulfonamide (**7c**) in DMSO; Figure S54: HR-MS spectrum of *N*-{[1-(4-fluorobenzyl)-1*H*-1,2,3-triazol-4-yl]methyl}-8-methoxyquinoline- 5-sulfonamide (**7c**); Figure S55: ^1^H NMR spectrum of *N*-{[1-(4-chlorophenyl)-1*H*-1,2,3-triazol-4-yl]methyl}-8-methoxyquinoline-5-sulfonamide (**7d**) in DMSO; Figure S56: ^13^C NMR spectrum of *N*-{[1-(4-chlorophenyl)-1*H*-1,2,3-triazol-4-yl]methyl}-8-methoxyquinoline- 5-sulfonamide (**7d**) in DMSO; Figure S57: HR-MS spectrum of *N*-{[1-(4-chlorophenyl)-1*H*-1,2,3-triazol-4-yl]methyl}-8-methoxyquinoline-5-sulfonamide (**7d**); Figure S58: ^1^H NMR spectrum of *N*-({1-[(phenylthio)methyl]-1*H*-1,2,3-triazol-4-yl}methyl)- 8-methoxyquinoline-5-sulfonamide (**7e**) in CDCl_3_; Figure S59: ^13^C NMR spectrum of *N*-({1-[(phenylthio)methyl]-1*H*-1,2,3-triazol-4-yl}methyl)-8-methoxyquinoline-5-sulfonamide (**7e**) in CDCl_3_; Figure S60: HR-MS spectrum of *N*-({1-[(phenylthio)methyl]-1*H*-1,2,3-triazol-4-yl}methyl)- 8-methoxyquinoline-5-sulfonamide (**7e**); Figure S61: ^1^H NMR spectrum of *N*-[2-(1-benzyl-1*H*-1,2,3-triazol-4-yl)propan-2-yl]-8-methoxyquinoline-5-sulfonamide (**7f**) in CDCl_3_; Figure S62: ^13^C NMR spectrum of *N*-[2-(1-benzyl-1*H*-1,2,3-triazol-4-yl)propan-2-yl]- 8-methoxyquinoline-5-sulfonamide (**7f**) in DMSO; Figure S63: HR-MS spectrum of *N*-[2-(1-benzyl-1*H*-1,2,3-triazol-4-yl)propan-2-yl]-8-methoxyquinoline-5-sulfonamide (**7f**); Figure S64: ^1^H NMR spectrum of *N*-methyl-*N*-[(1-benzyl-1*H*-1,2,3-triazol-4-yl)methyl]- 8-methoxyquinoline-5-sulfonamide (**7g**) in CDCl_3_; Figure S65: ^13^C NMR spectrum of *N*-methyl- *N*-[(1-benzyl-1*H*-1,2,3-triazol-4-yl)methyl]-8-methoxyquinoline-5-sulfonamide (**7g**) in CDCl_3_; Figure S66: HR-MS spectrum of *N*-methyl-*N*-[(1-benzyl-1*H*-1,2,3-triazol-4-yl)methyl]- 8-methoxyquinoline-5-sulfonamide (**7g**); Figure S67: ^1^H NMR spectrum of *N*-{4-[(1-benzyl-1*H*-1,2,3-triazol-4-yl)methoxy]phenyl}-8-methoxyquinoline-5-sulfonamide (**7h**) in DMSO; Figure S68: ^13^C NMR spectrum of *N*-{4-[(1-benzyl-1*H*-1,2,3-triazol-4-yl)methoxy]phenyl}- 8-methoxyquinoline-5-sulfonamide (**7h**) in DMSO; Figure S69: HR-MS spectrum of *N*-{4-[(1-benzyl-1*H*-1,2,3-triazol-4-yl)methoxy]phenyl}-8-methoxyquinoline-5-sulfonamide (**7h**).
