# Supplementary material for: Design, Synthesis, and Anticancer and Antibacterial Activities of Quinoline-5-Sulfonamides
Source: Molecules. 2024 Aug 26;29(17):4044. doi: 10.3390/molecules29174044 (PMC11396667; doi:10.3390/molecules29174044)
Supplement: Supplementary file 1 [file molecules-29-04044-s001.zip › molecules-3101338-supplementary.pdf]

Supplementary materials

Design, Synthesis, and Anticancer and Antibacterial Activities of Quinoline-5-sulfonamides

Andrzej Zieba [1,*], Dominika Pindjakova [2], Malgorzata Latocha [3], Justyna Plonka-Czerw [3], Dariusz Kusmierz [3], Alois Cizek [2] and Josef Jampilek [4,*]

[1] Department of Organic Chemistry, Faculty of Pharmaceutical Sciences in Sosnowiec, Medical University of Silesia, Jagiellonska 4, 41-200 Sosnowiec, Poland
[2] Department of Infectious Diseases and Microbiology, Faculty of Veterinary Medicine, University of Veterinary Sciences Brno, Palackeho 1946/1, 612 42 Brno, Czech Republic; pindjakova.dominika@gmail.com (D.P.); cizeka@vfu.cz (A.C.)
[3] Department of Cell Biology, Faculty of Pharmaceutical Sciences in Sosnowiec, Medical University of Silesia, Jednosci 9, 41-200 Sosnowiec, Poland; mlatocha@sum.edu.pl (M.L.); jplonka@sum.edu.pl (J.P.), dkusmierz@sum.edu.pl (D.K.)
[4] Institute of Chemistry, University of Silesia, Szkolna 9, 40-007 Katowice, Poland
***** Correspondence: zieba@sum.edu.pl (A.Z.); josef.jampilek@gmail.com (J.J.)

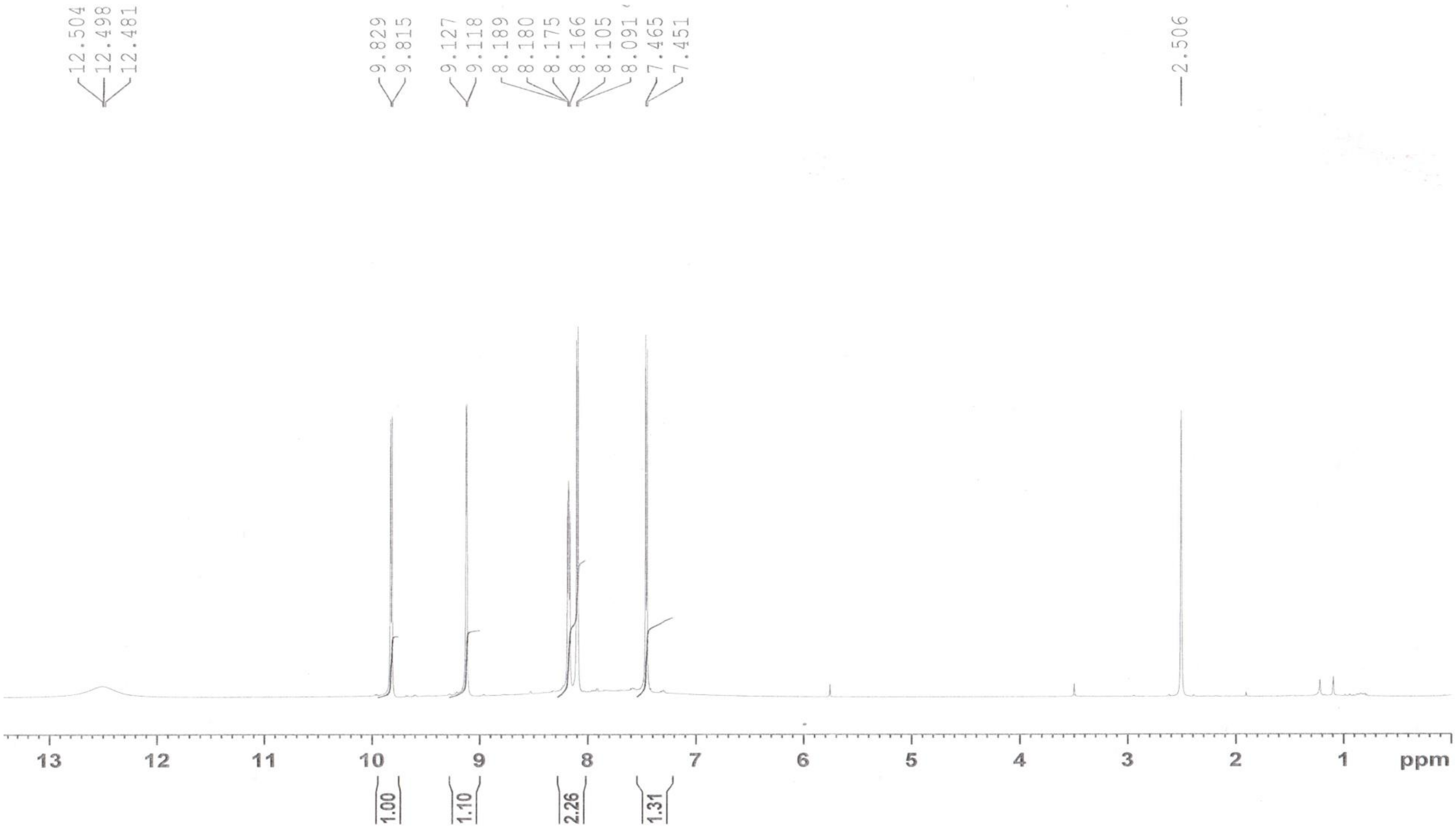

Figure S1. ^{1}H NMR spectrum of 8-hydroxyquinoline-5-sulfonyl chloride (**2**) in DMSO.

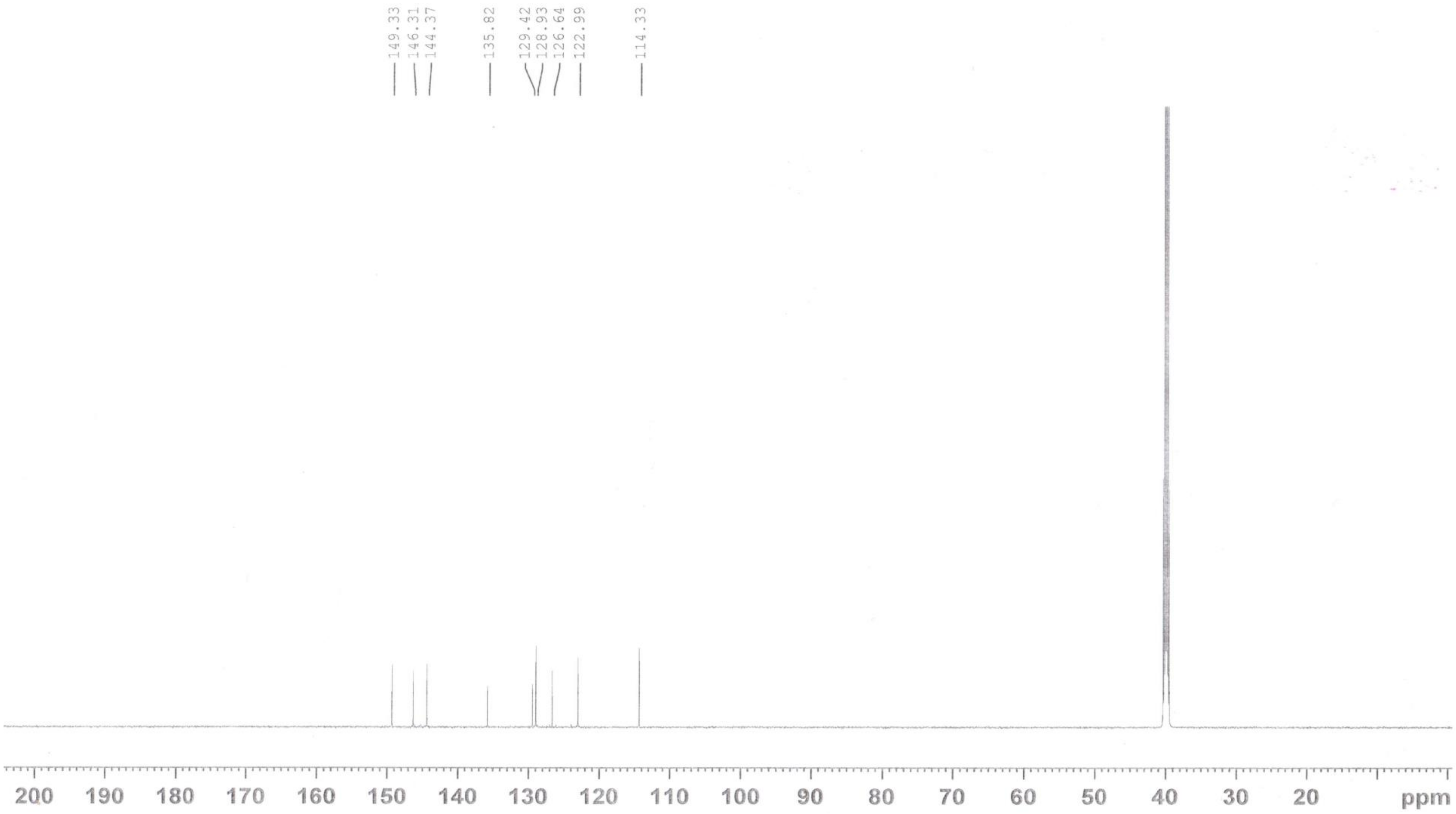

Figure S2. ^{13}C NMR spectrum of 8-hydroxyquinoline-5-sulfonyl chloride (**2**) in DMSO.

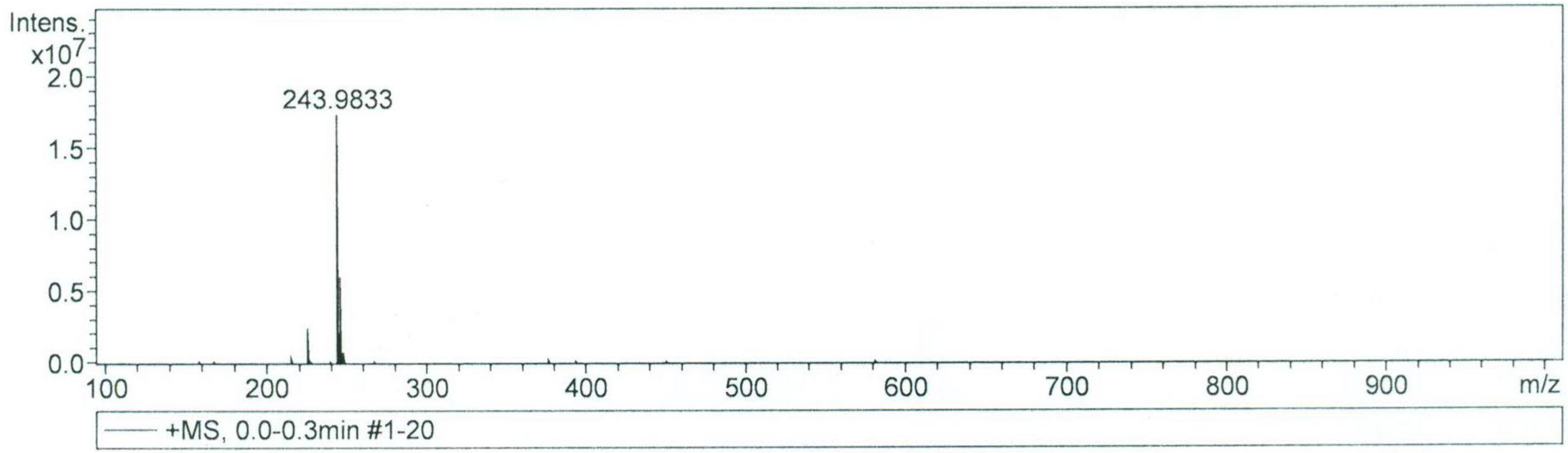

#	m/z	Res.	S/N	I	I %	FWHM
1	243.9833	33041	62928.4	17314926	100.0	0.0074
2	245.9801	34178	22121.5	6101592	35.2	0.0072

Figure S3. HR-MS spectrum of 8-hydroxyquinoline-5-sulfonyl chloride (**2**).

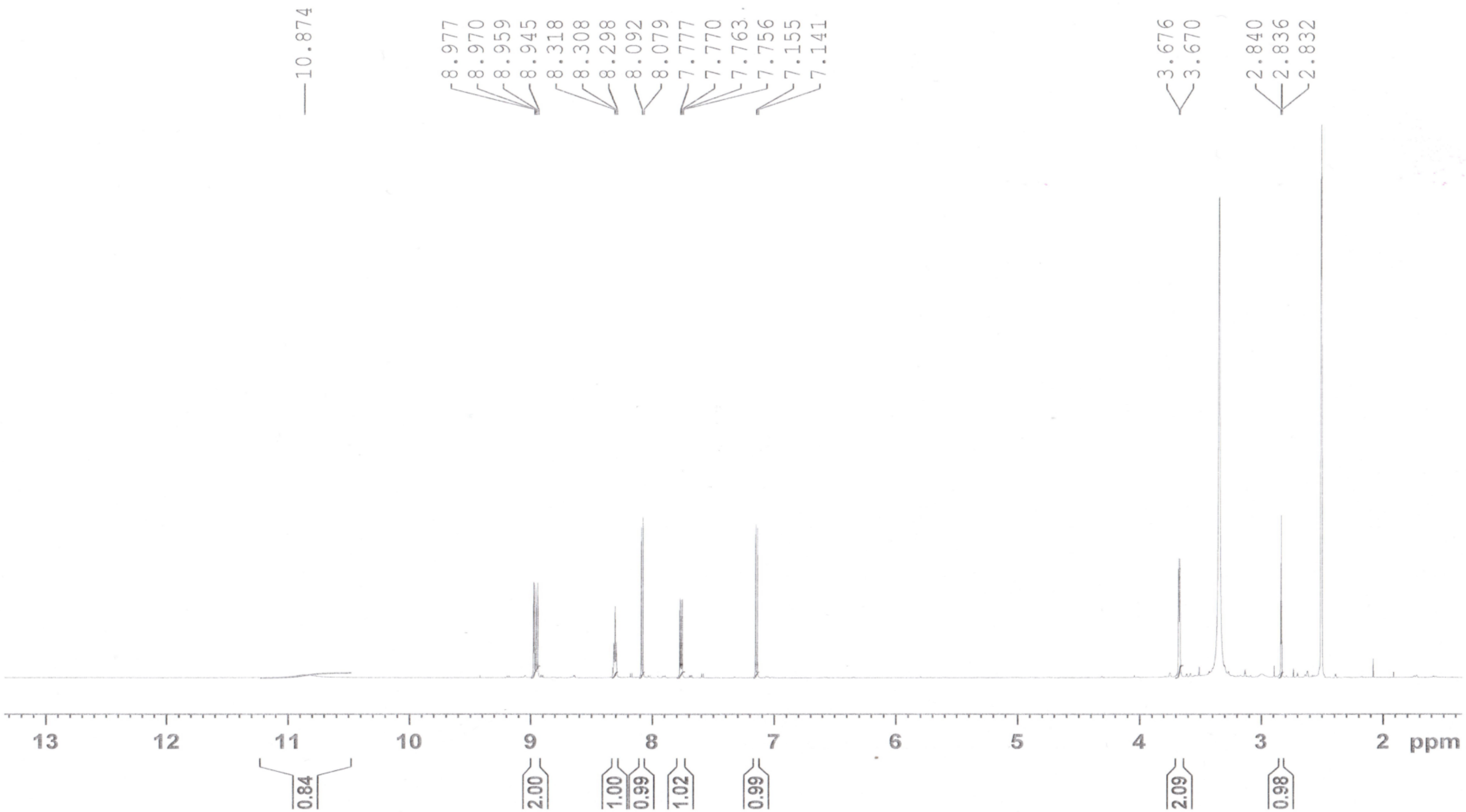

Figure S4. ^{1}H NMR spectrum of 8-hydroxy-*N*-(prop-2-yn-1-yl)quinoline-5-sulfonamide (**3a**) in DMSO.

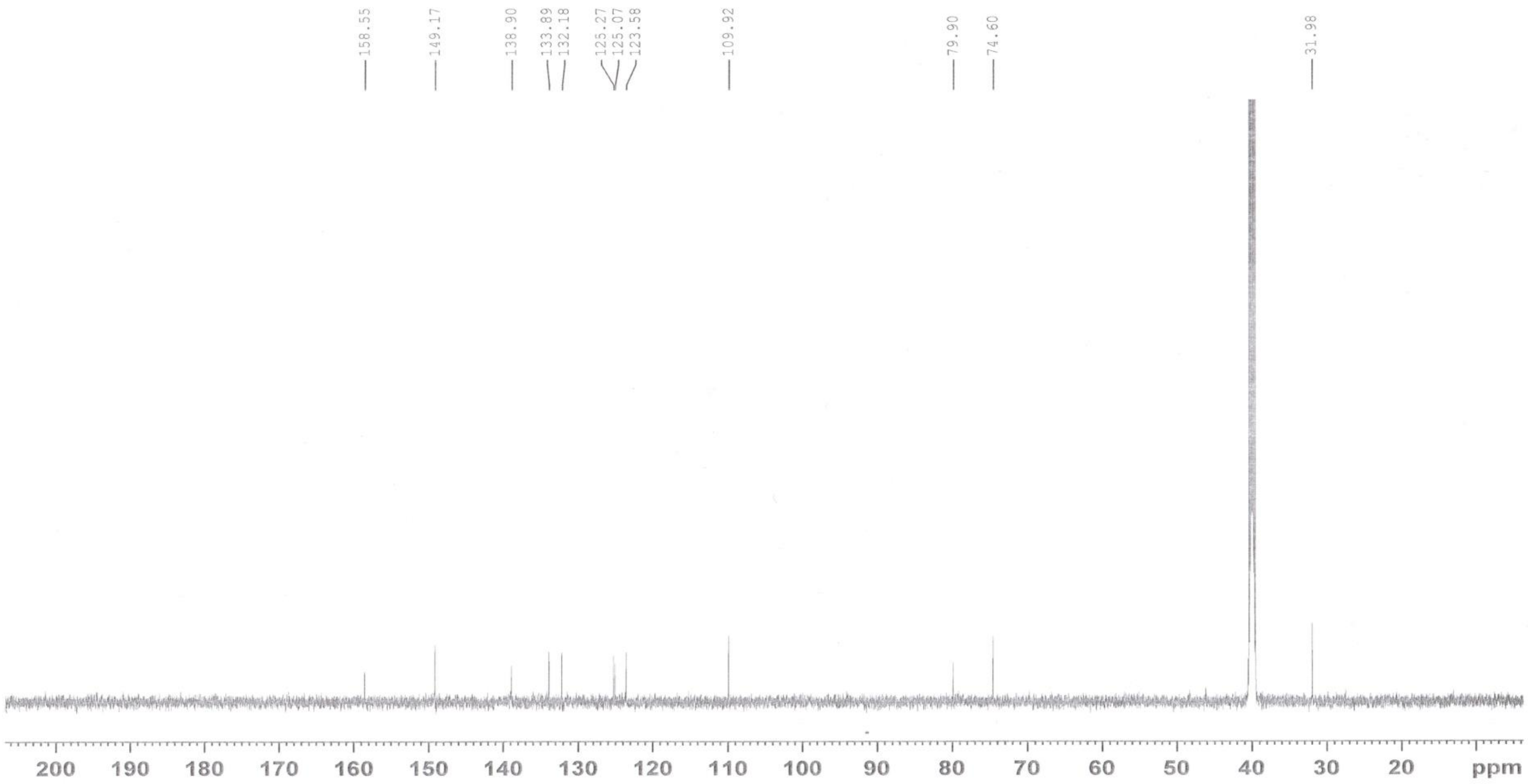

Figure S5. ^{13}C NMR spectrum of 8-hydroxy-*N*-(prop-2-yn-1-yl)quinoline-5-sulfonamide (**3a**) in DMSO.

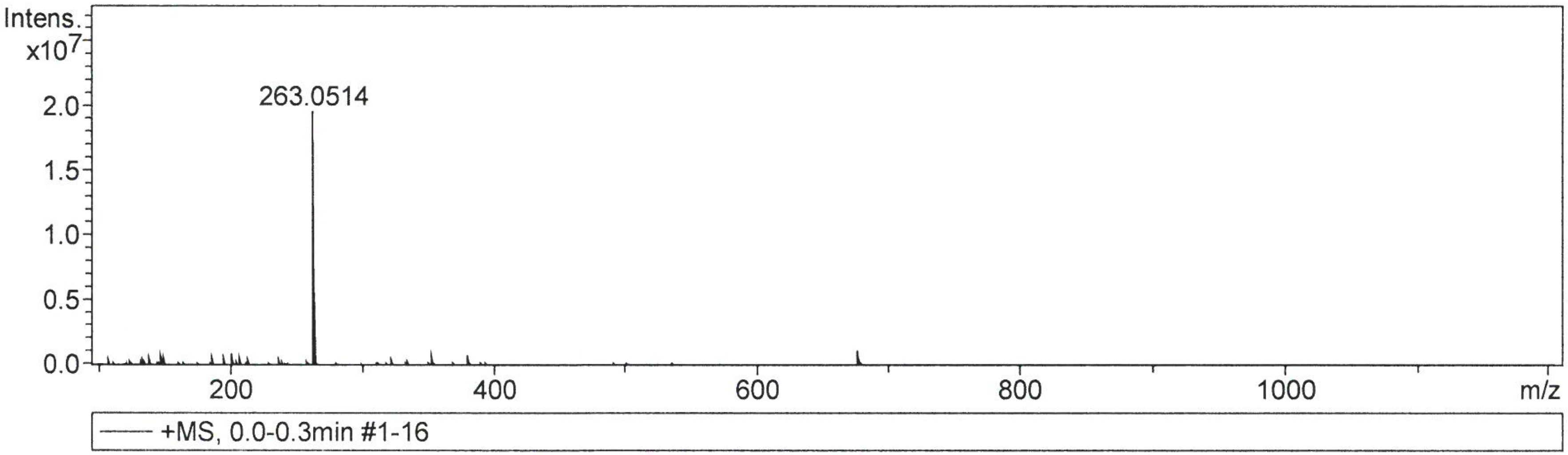

#	m/z	Res.	S/N	I	I %	FWHM
1	263.0514	14368	74712.0	19485056	100.0	0.0183

Figure S6. HR-MS spectrum of 8-hydroxy-*N*-(prop-2-yn-1-yl)quinoline-5-sulfonamide (**3a**).

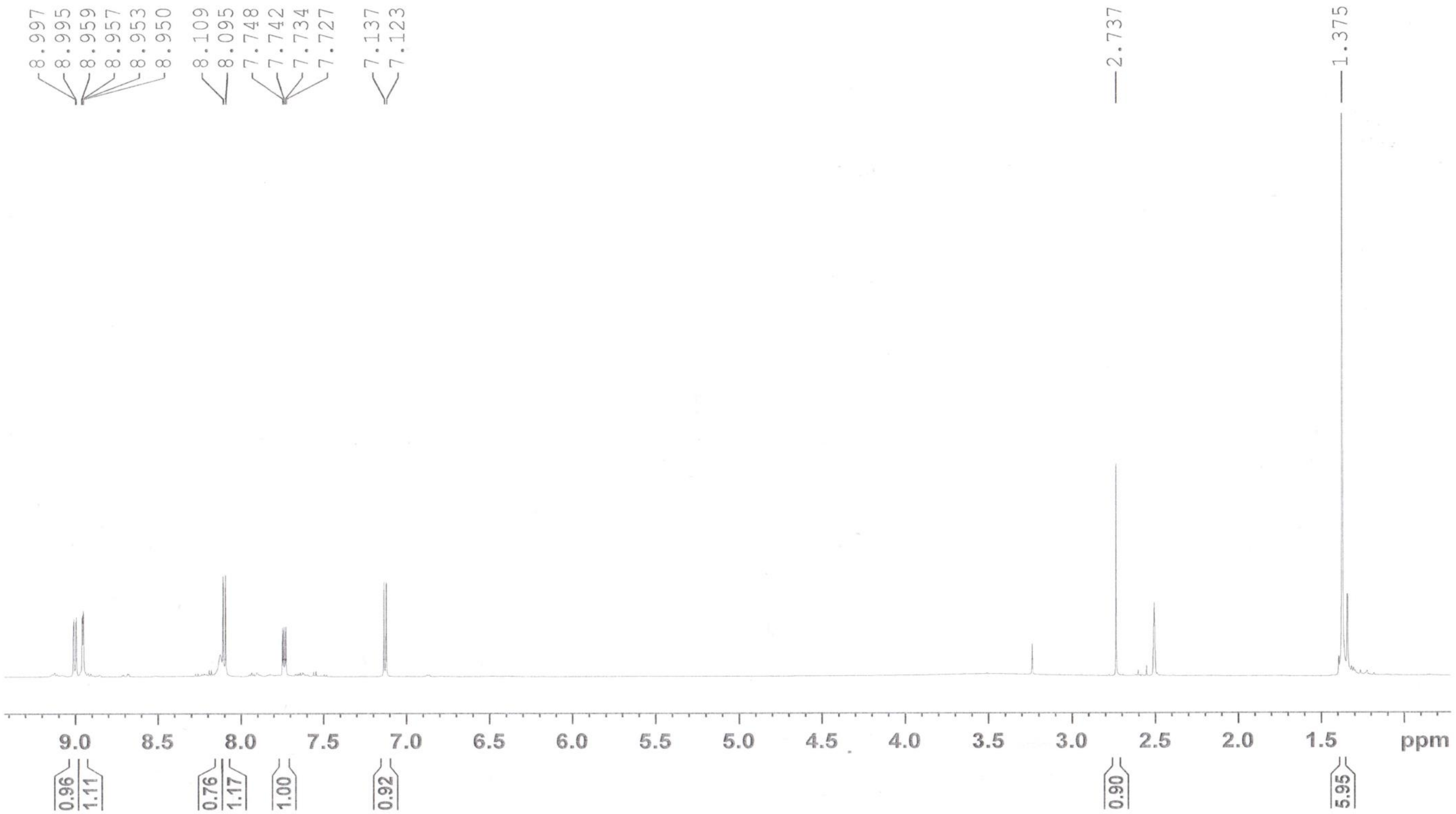

Figure S7. ^{1}H NMR spectrum of 8-hydroxy-*N*-(1,1-dimethylprop-2-yn-1-yl)quinoline-5-sulfonamide (**3b**) in DMSO.

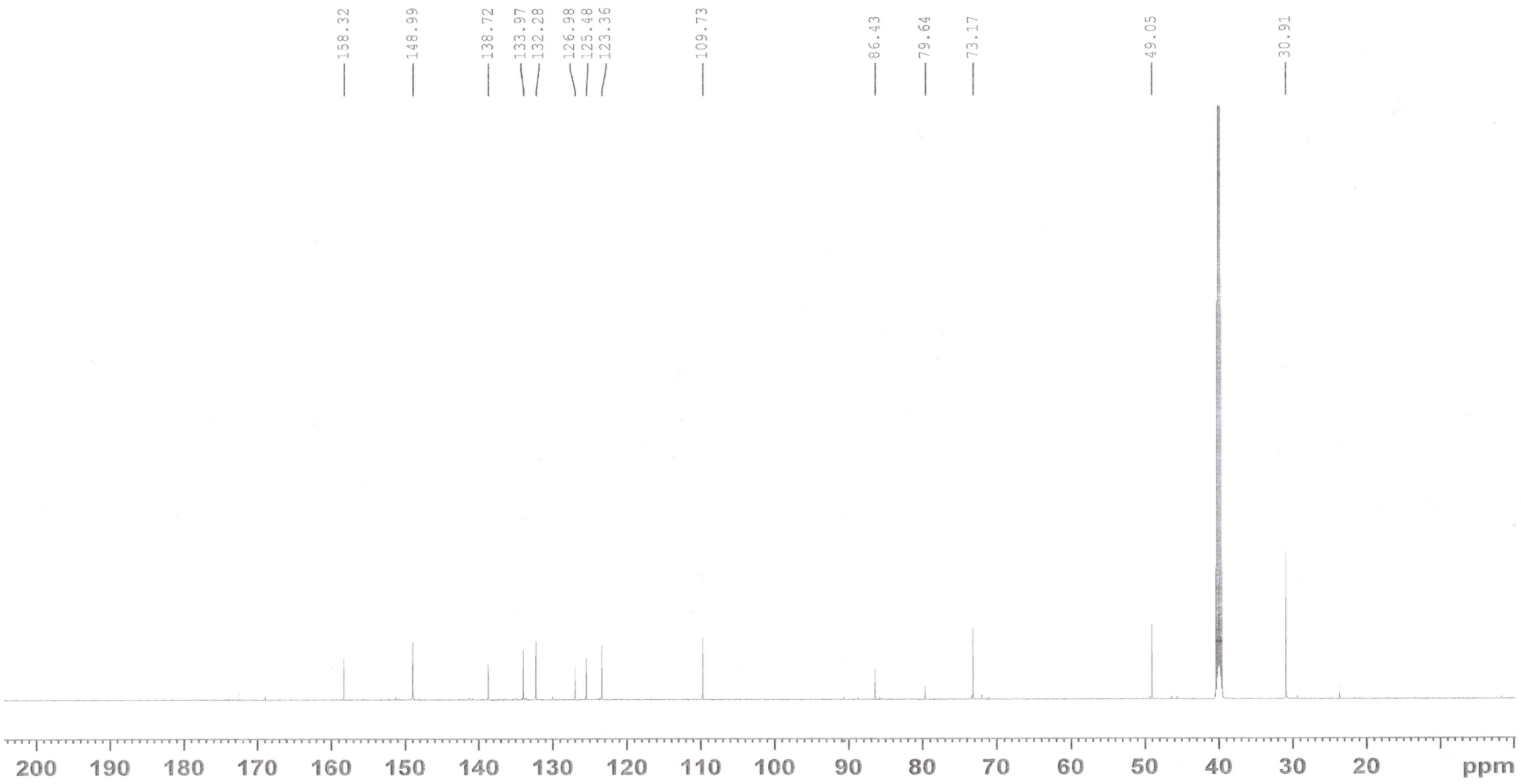

Figure S8. ^{13}C NMR spectrum of 8-hydroxy-*N*-(1,1-dimethylprop-2-yn-1-yl)quinoline-5-sulfonamide (**3b**) in DMSO.

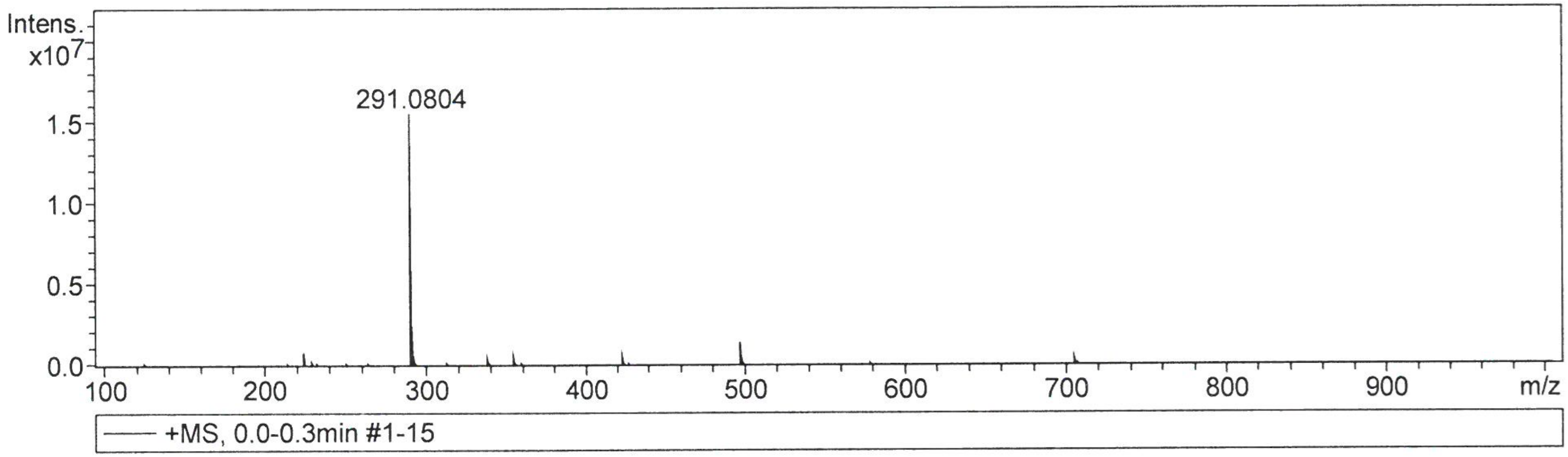

#	m/z	Res.	S/N	I	I %	FWHM
1	291.0804	37480	56653.8	15459219	100.0	0.0078

Figure S9. HR-MS spectrum of 8-hydroxy-*N*-(1,1-dimethylprop-2-yn-1-yl)quinoline-5-sulfonamide (**3b**).

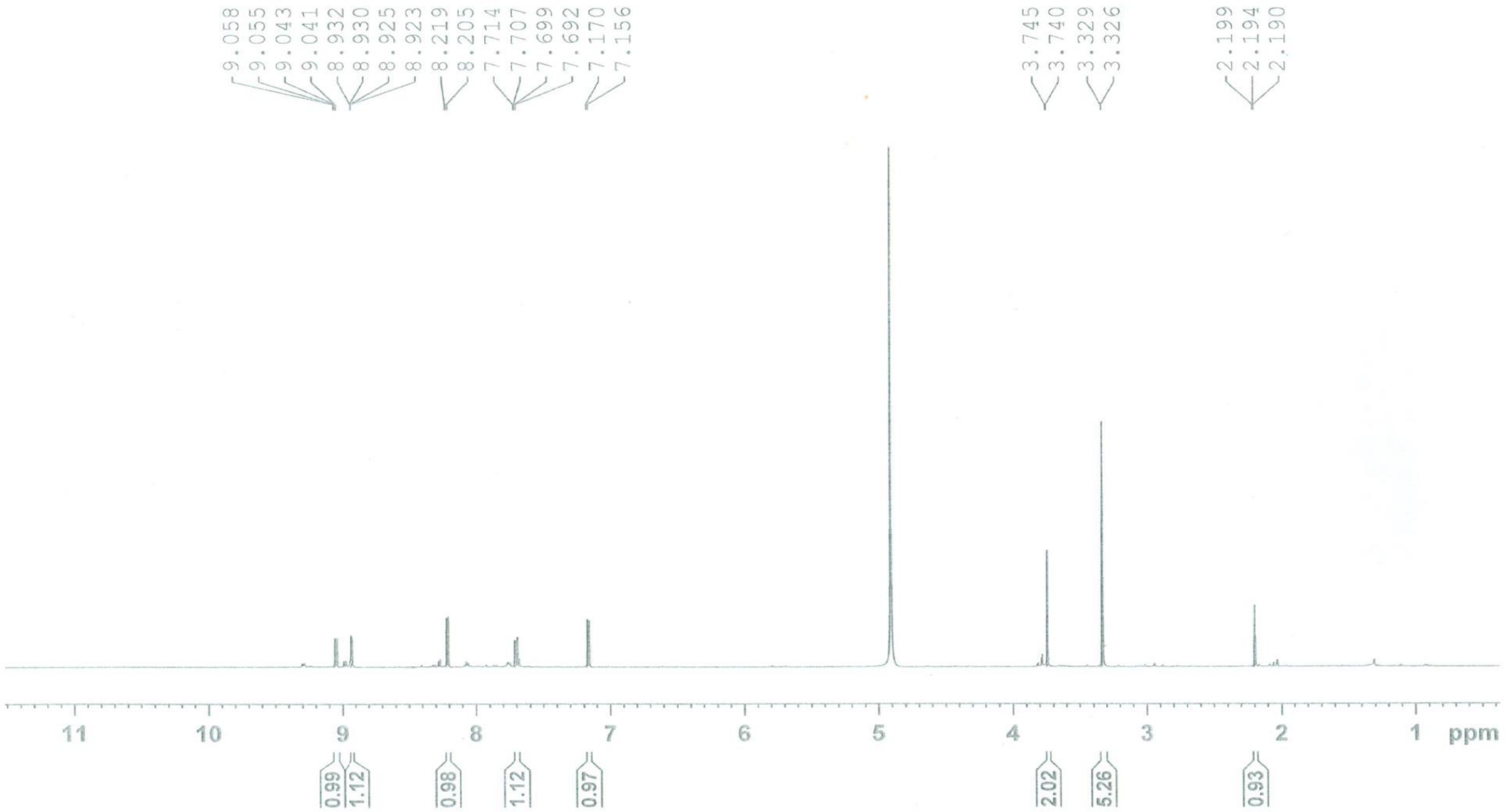

Figure S10. ^{1}H NMR spectrum of 8-hydroxy-*N*-methyl-*N*-(prop-2-yn-1-yl)quinoline-5-sulfonamide (**3c**) in CD_3OD.

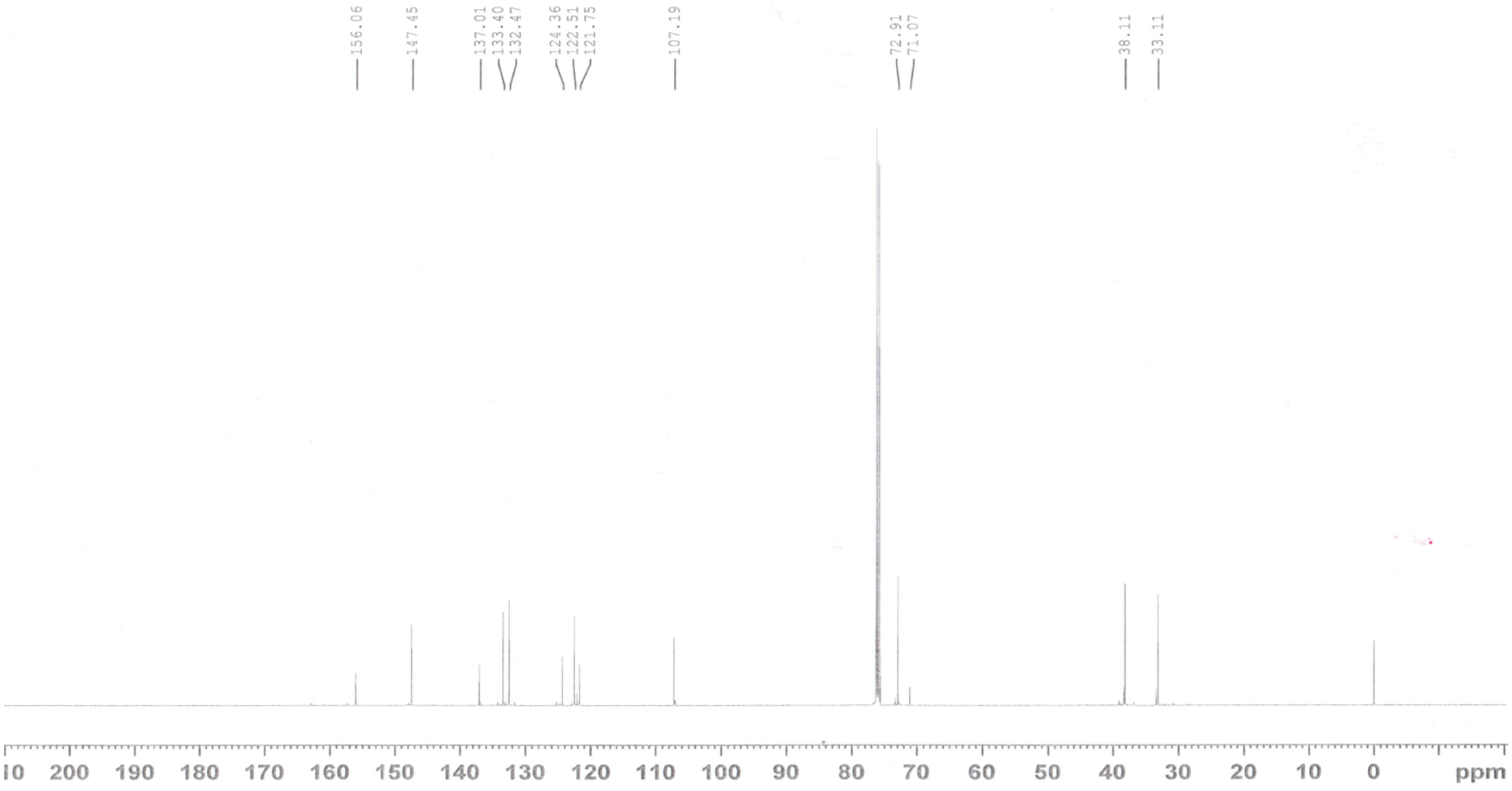

Figure S11. ^{13}C NMR spectrum of 8-hydroxy-*N*-methyl-*N*-(prop-2-yn-1-yl)quinoline-5-sulfonamide (**3c**) in $CDCl_3$.

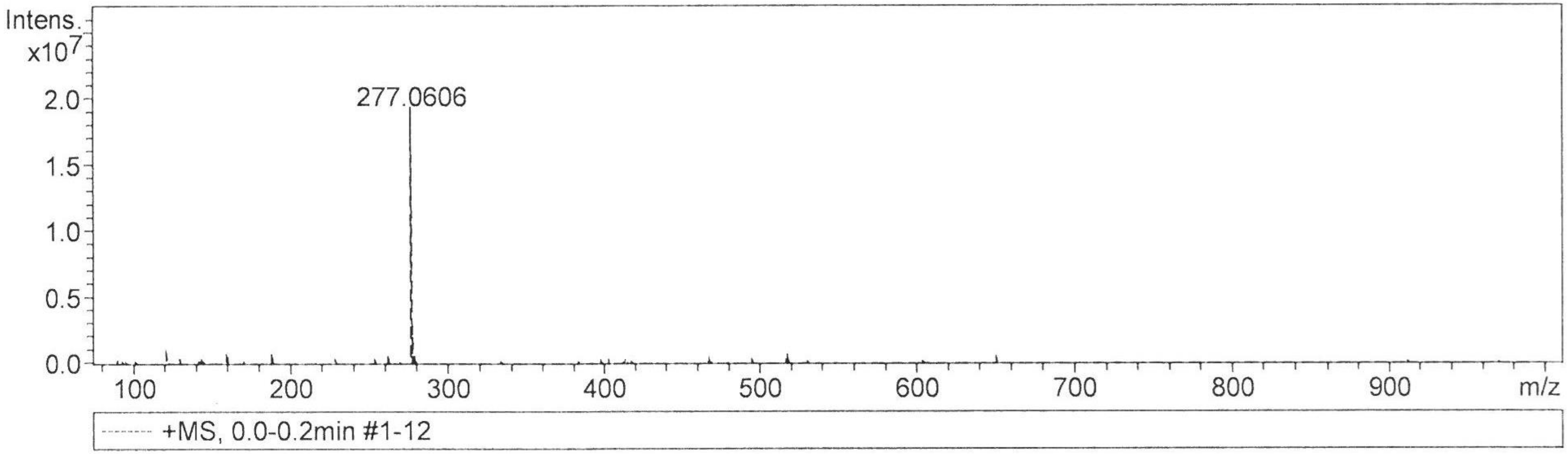

#	m/z	Res.	S/N	I	I %	FWHM
1	277.0606	32644	116387.5	18950882	100.0	0.0085

Figure S12. HR-MS spectrum of 8-hydroxy-*N*-methyl-*N*-(prop-2-yn-1-yl)quinoline-5-sulfonamide (**3c**).

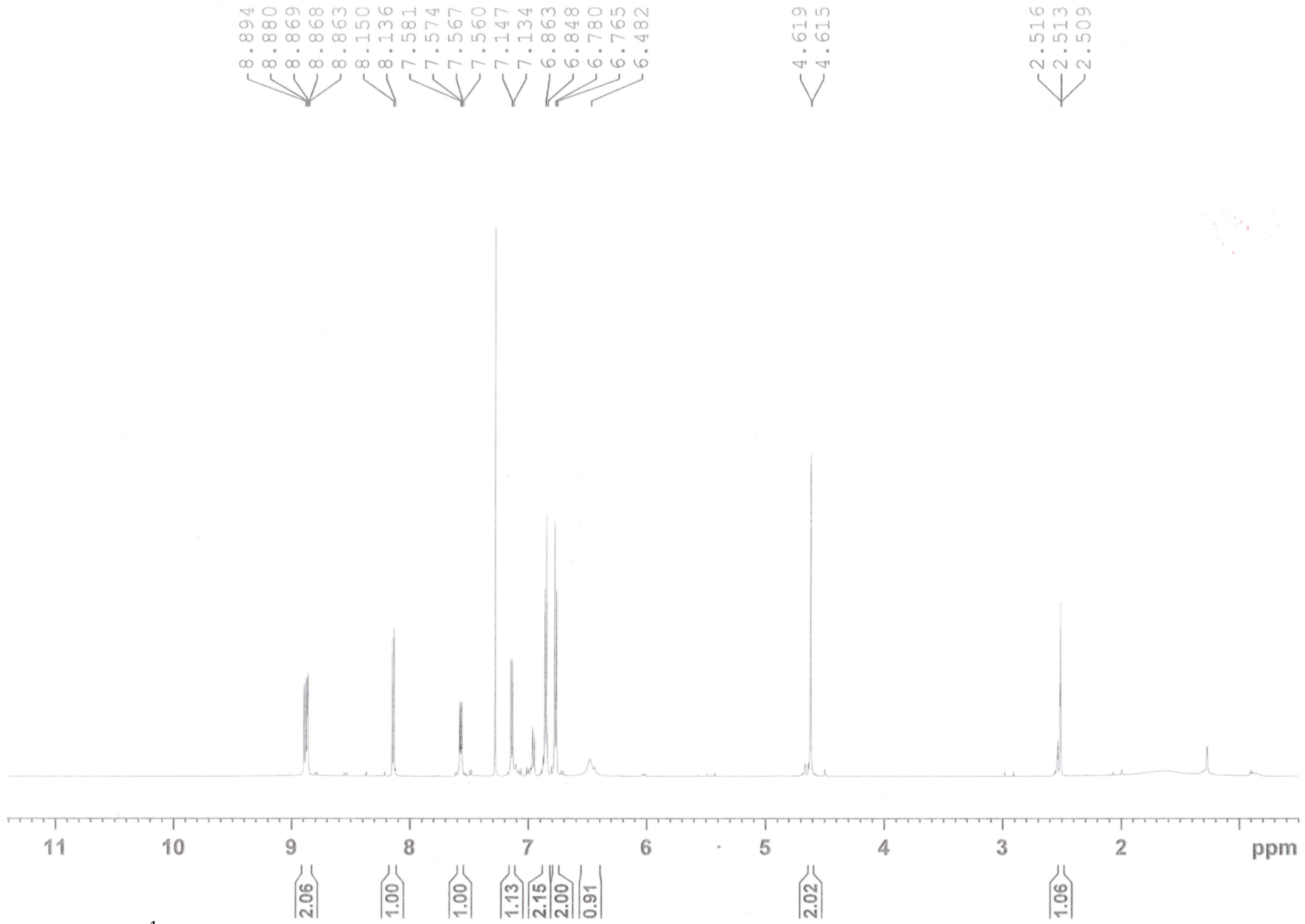

Figure S13. ^{1}H NMR spectrum of 8-hydroxy-*N*-[4-(prop-2-yn-1-yloxy)phenyl]quinoline-5-sulfonamide (**3d**) in $CDCl_3$.

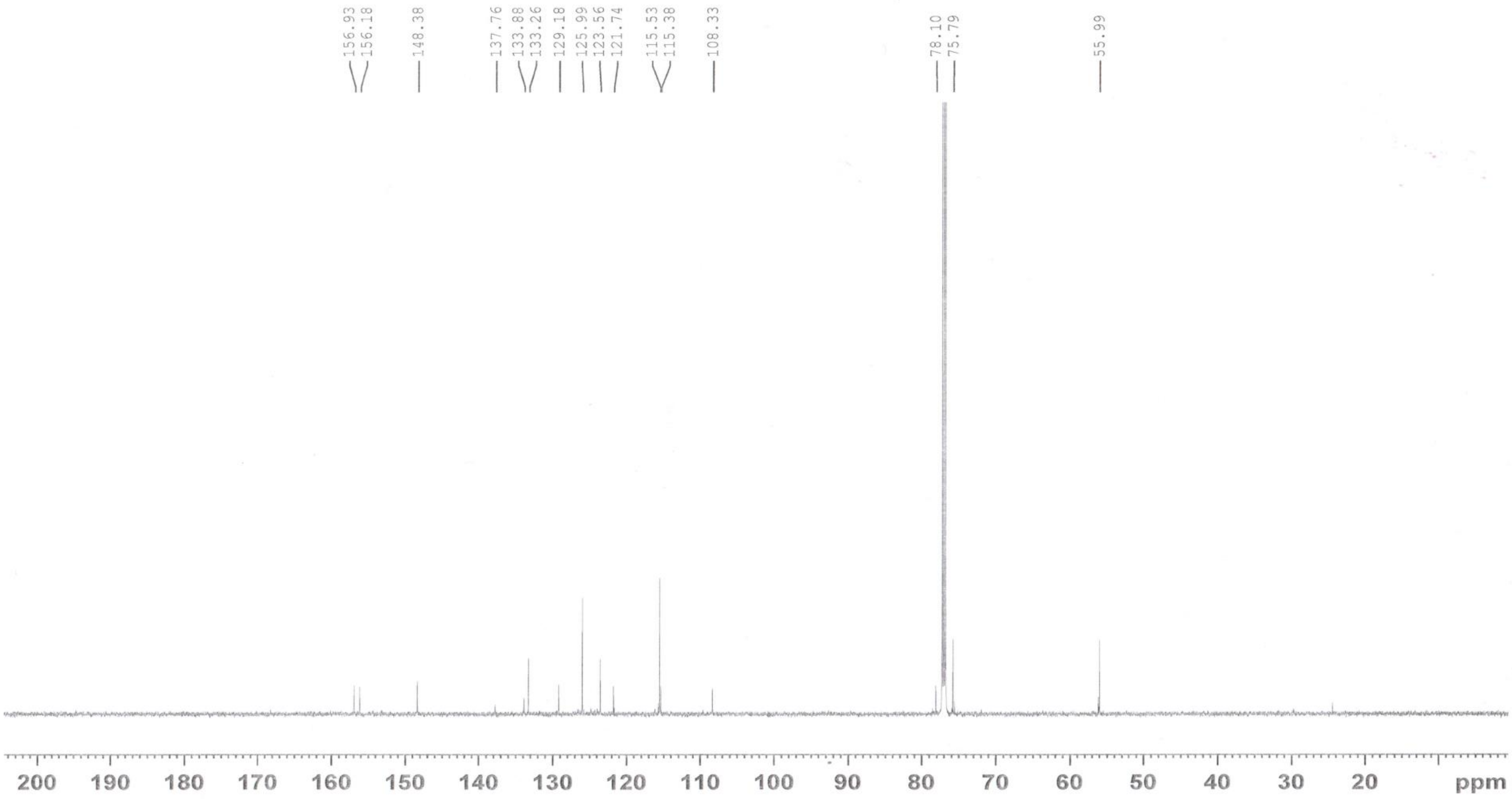

Figure S14. ^{13}C NMR spectrum of 8-hydroxy-*N*-[4-(prop-2-yn-1-yloxy)phenyl]quinoline-5-sulfonamide (**3d**) in $CDCl_3$.

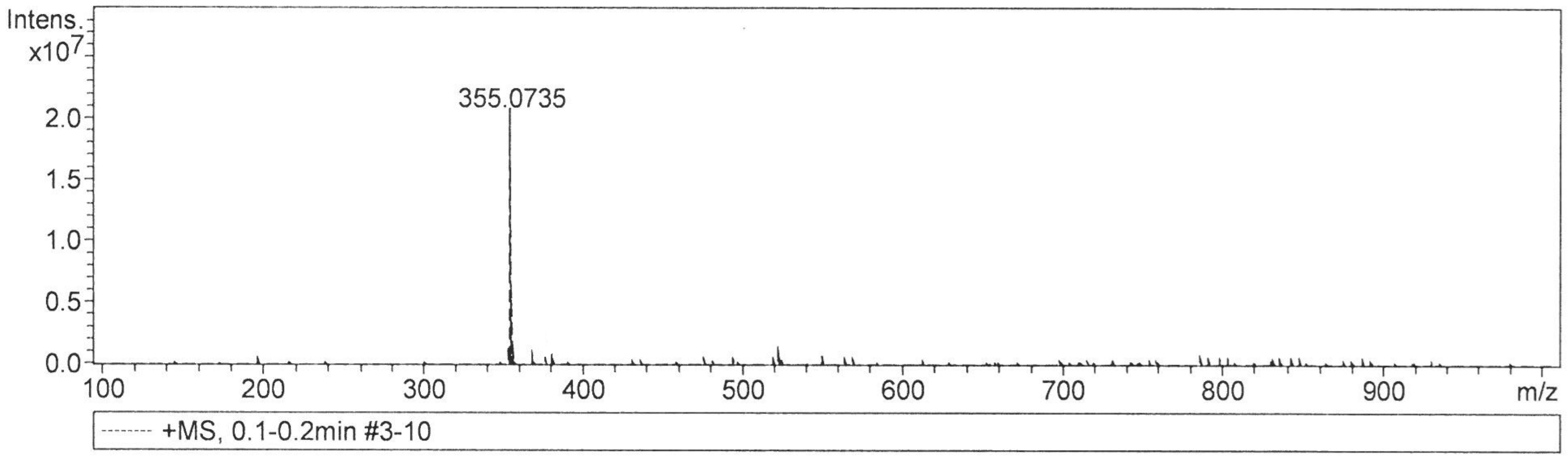

#	m/z	Res.	S/N	I	I %	FWHM
1	355.0735	18915	63875.7	20376568	100.0	0.0188

Figure S15. HR-MS spectrum of 8-hydroxy-*N*-[4-(prop-2-yn-1-yloxy)phenyl]quinoline-5-sulfonamide (**3d**).

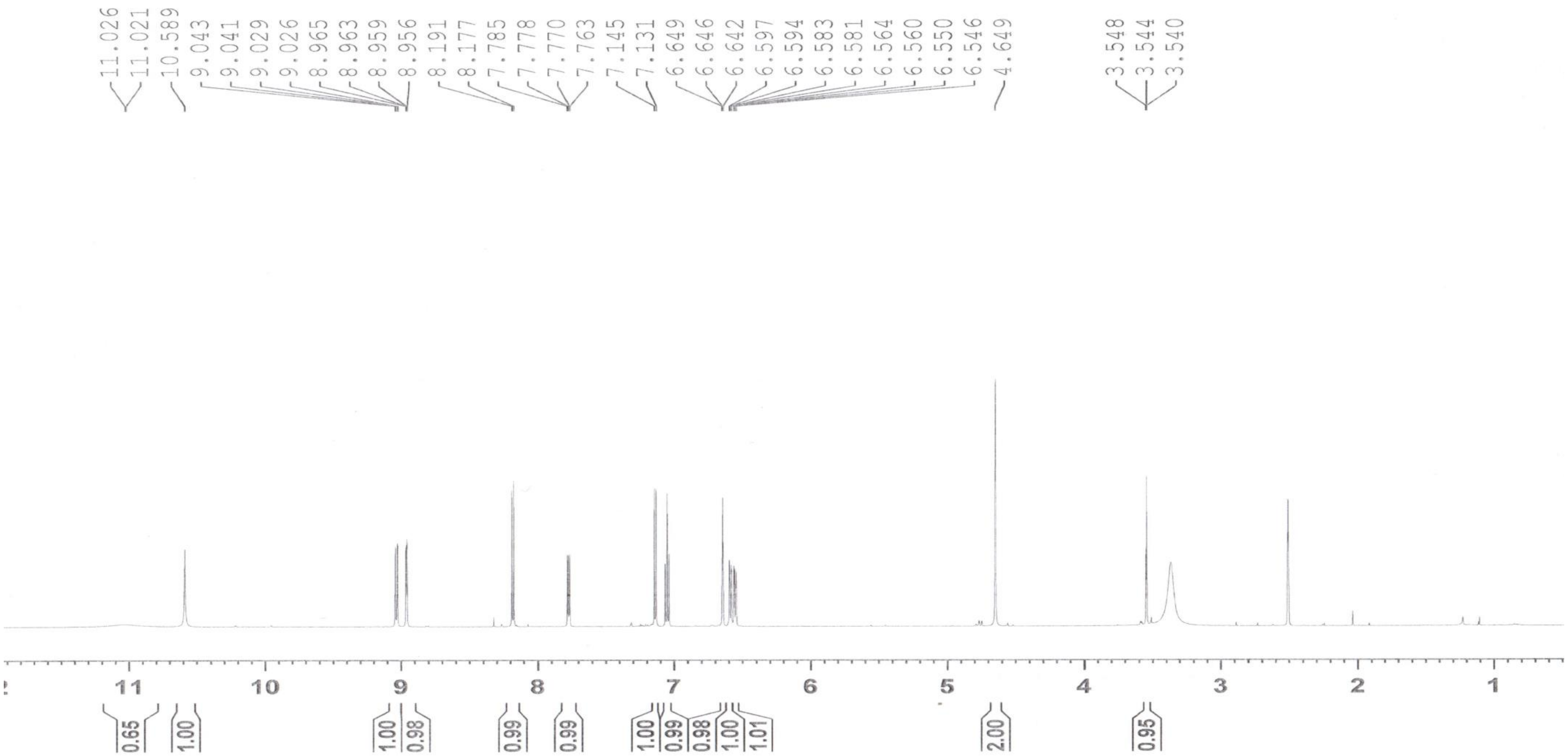

Figure S16. ^{1}H NMR spectrum of 8-hydroxy-*N*-[3-(prop-2-yn-1-yloxy)phenyl]quinoline-5-sulfonamide (**3e**) in DMSO.

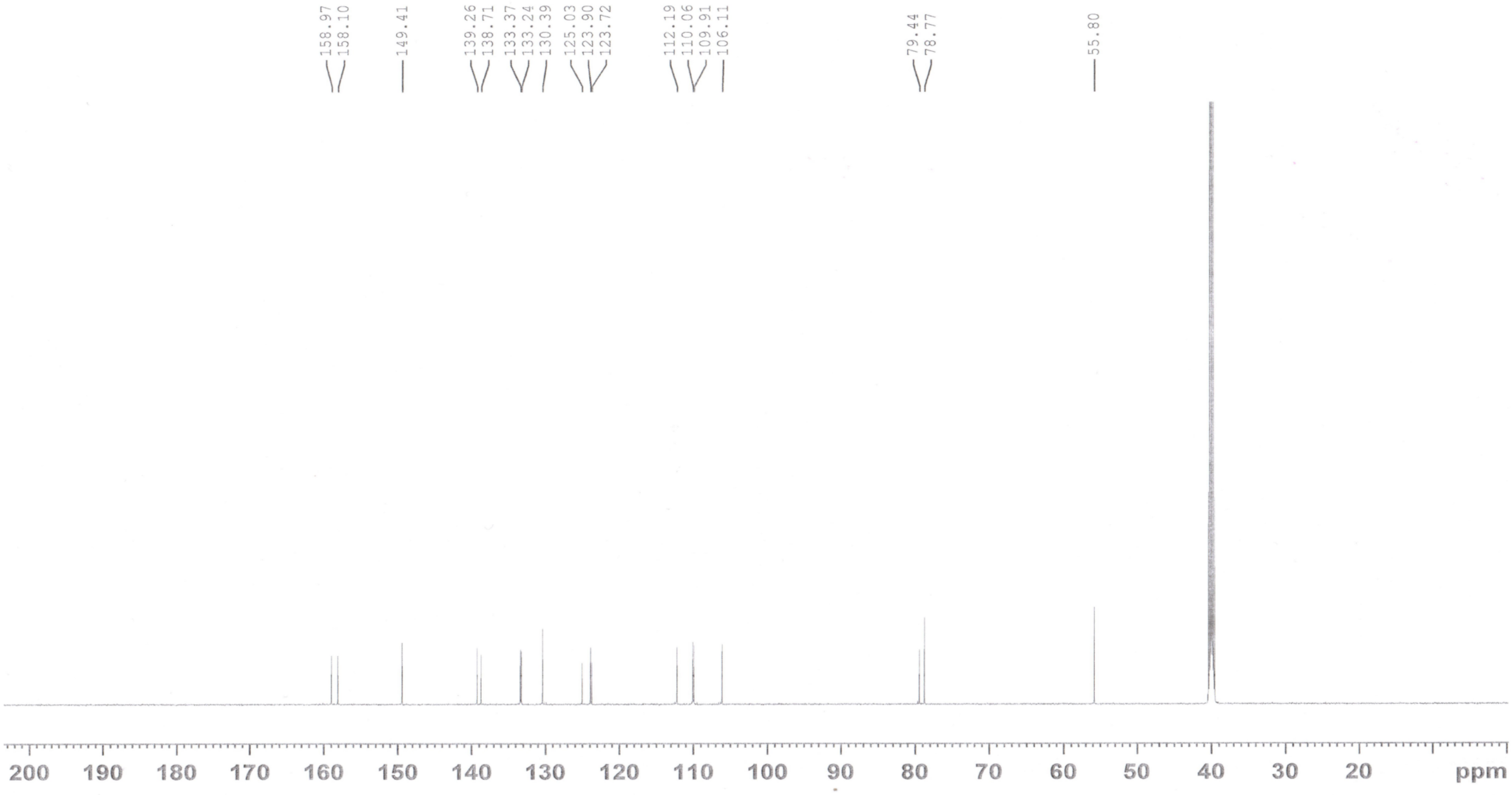

Figure S17. ^{13}C NMR spectrum of 8-hydroxy-*N*-[3-(prop-2-yn-1-yloxy)phenyl]quinoline-5-sulfonamide (**3e**) in DMSO.

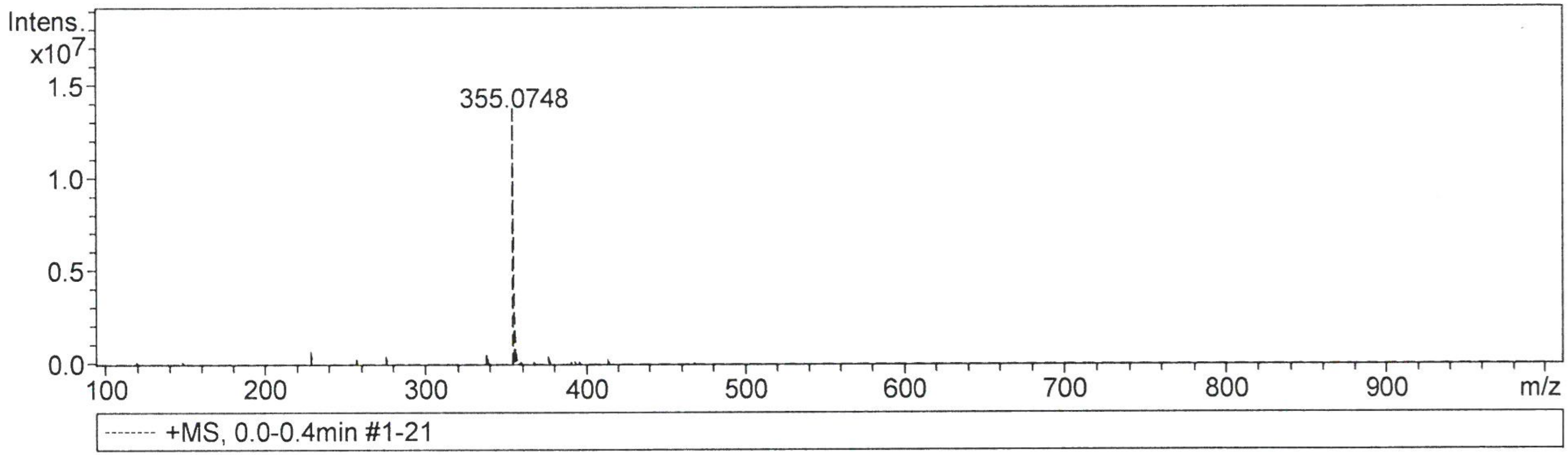

#	m/z	Res.	S/N	I	I %	FWHM
1	355.0748	41207	65142.5	13448462	100.0	0.0086

Figure S18. HR-MS spectrum of 8-hydroxy-*N*-[3-(prop-2-yn-1-yloxy)phenyl]quinoline-5-sulfonamide (**3e**).

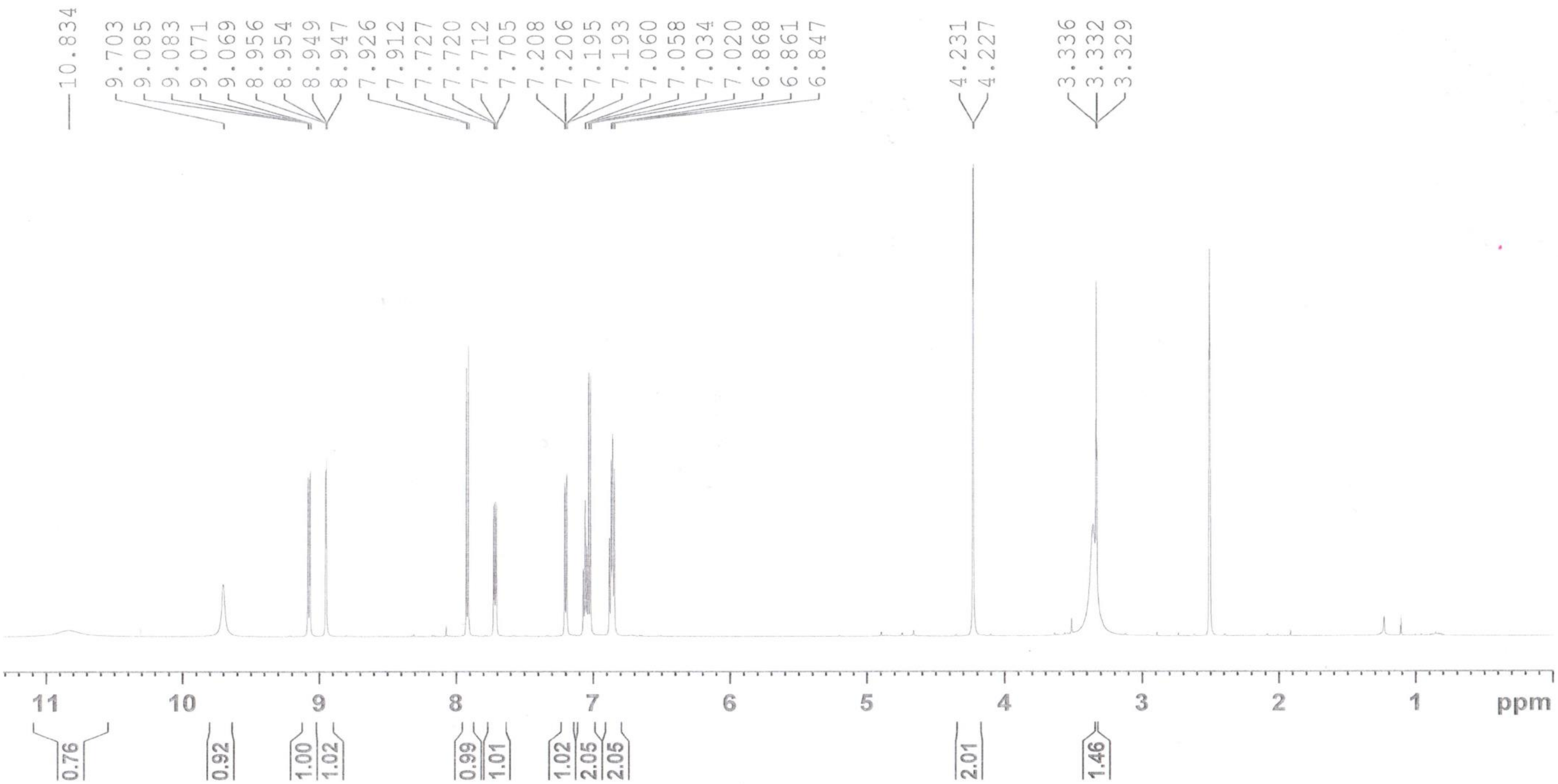

Figure S19. ^{1}H NMR spectrum of 8-hydroxy-*N*-[2-(prop-2-yn-1-yloxy)phenyl]quinoline-5-sulfonamide (**3f**) in DMSO.

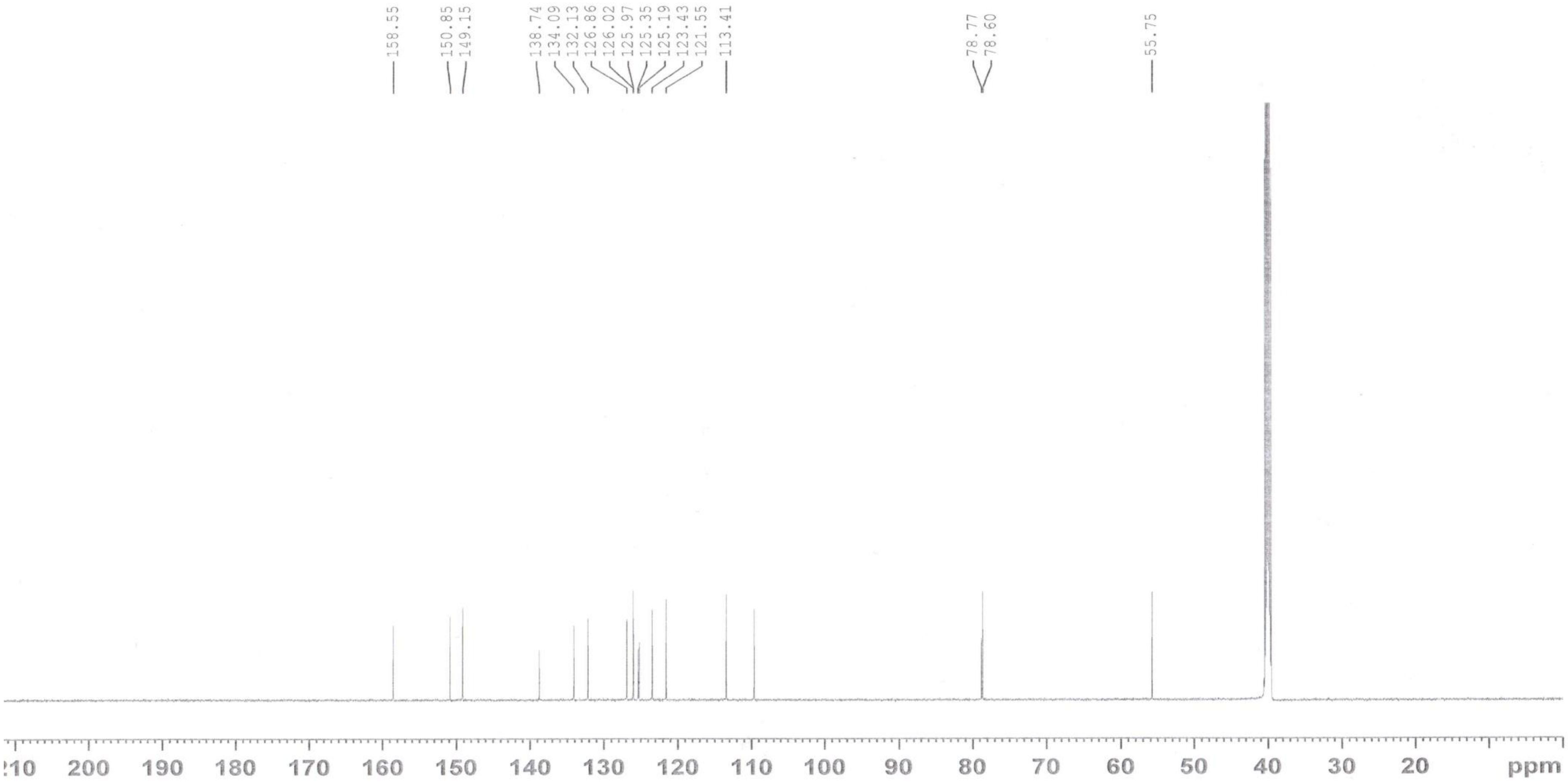

Figure 20. ^{13}C NMR spectrum of 8-hydroxy-*N*-[2-(prop-2-yn-1-yloxy)phenyl]quinoline-5-sulfonamide (**3f**) in DMSO.

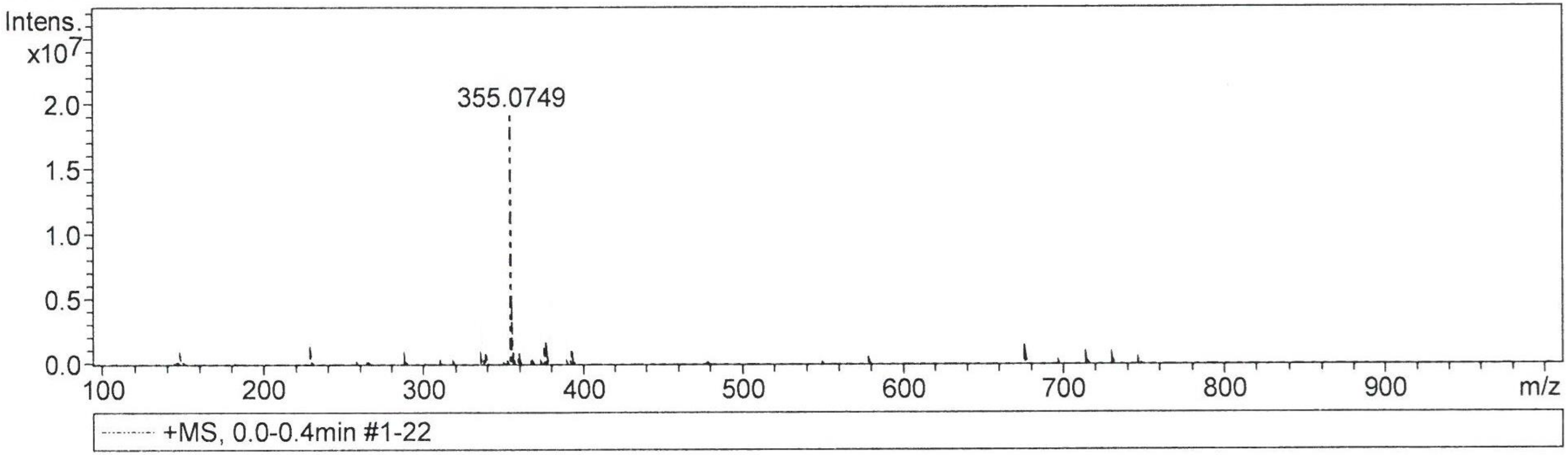

#	m/z	Res.	S/N	I	I %	FWHM
1	355.0749	32323	88628.4	19285532	100.0	0.0110

Figure 21. HR-MS spectrum of 8-hydroxy-*N*-[2-(prop-2-yn-1-yloxy)phenyl]quinoline-5-sulfonamide (**3f**).

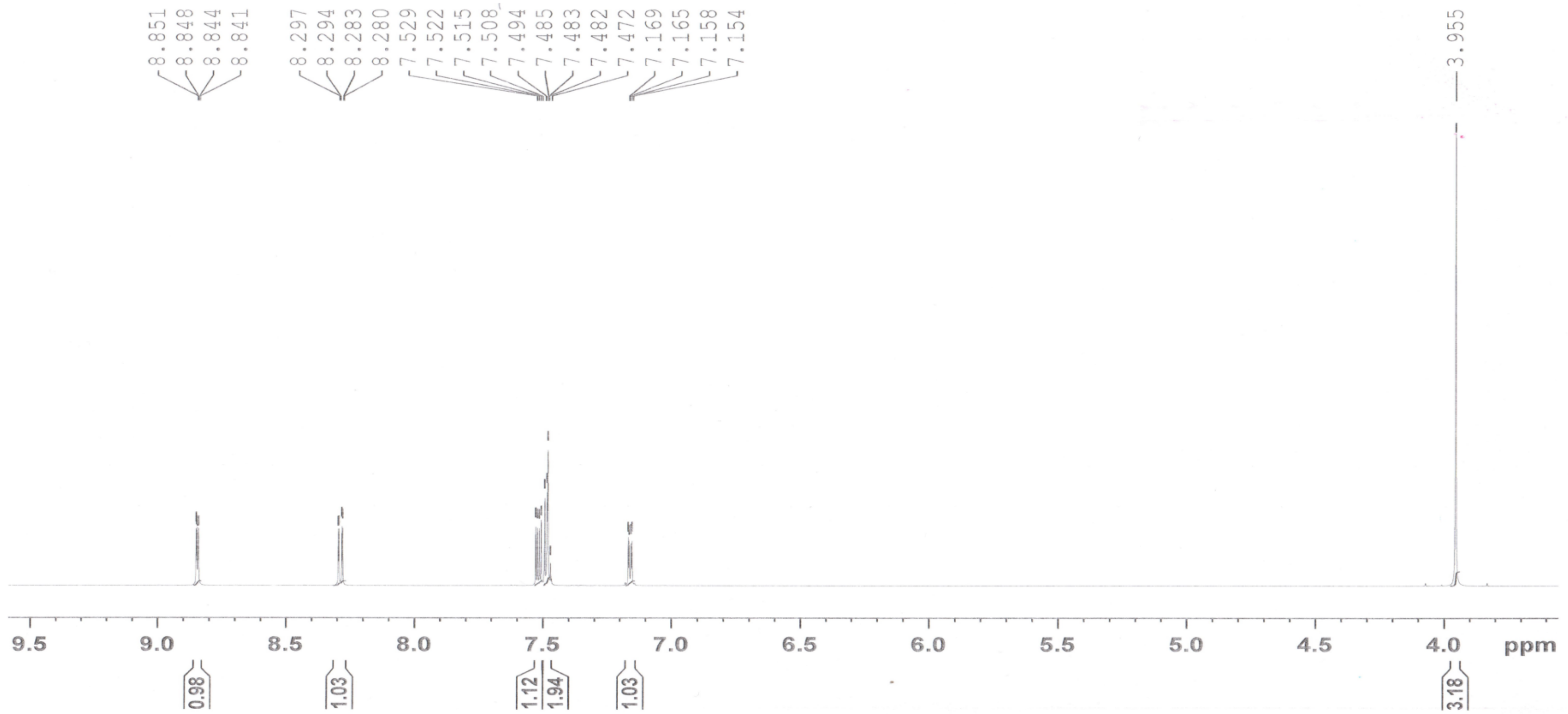

Figure S22. ^{1}H NMR spectrum of 8-methoxyquinoline (**4**) in DMSO.

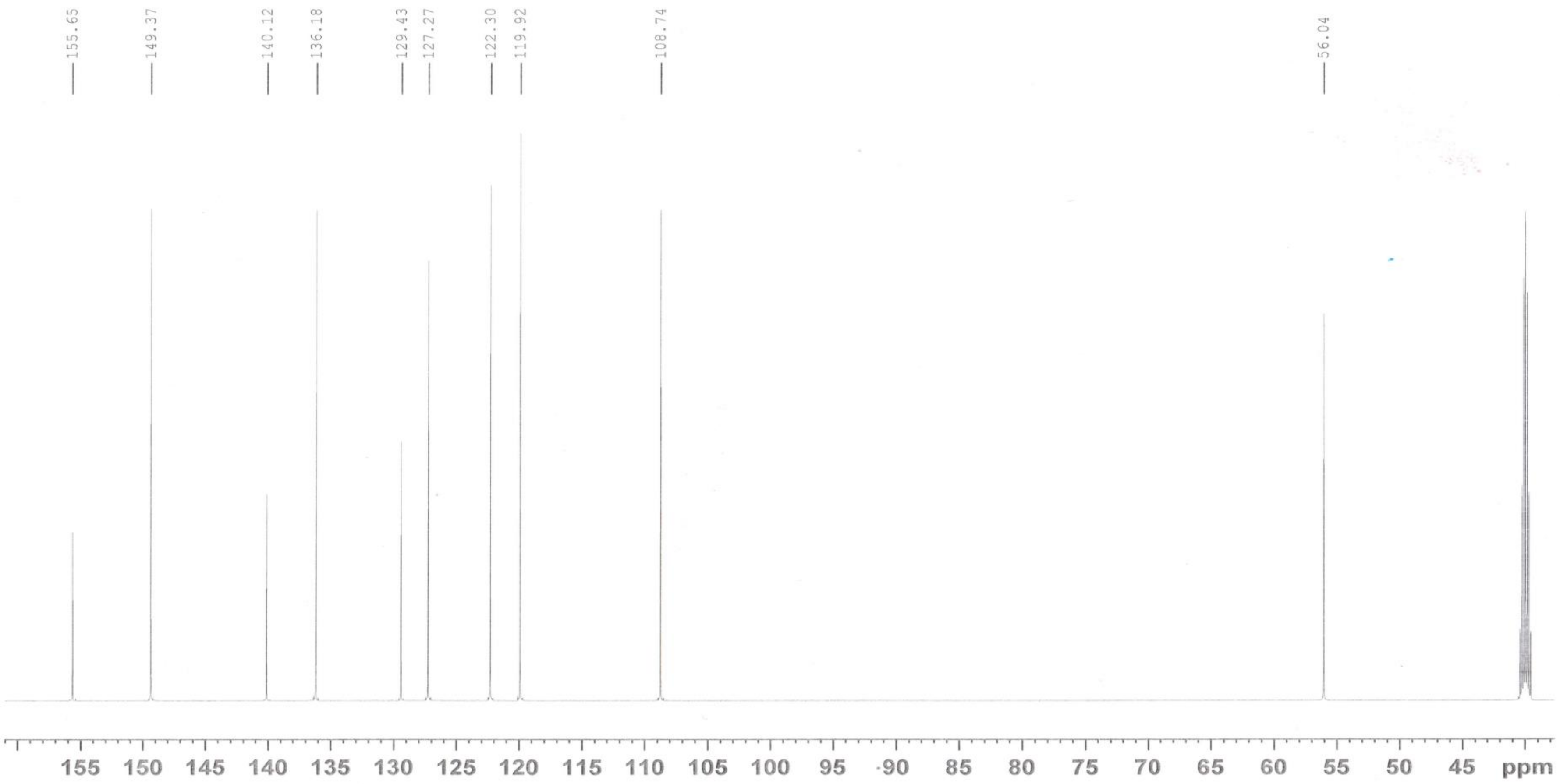

Figure S23. ^{13}C NMR spectrum of 8-methoxyquinoline (**4**) in DMSO.

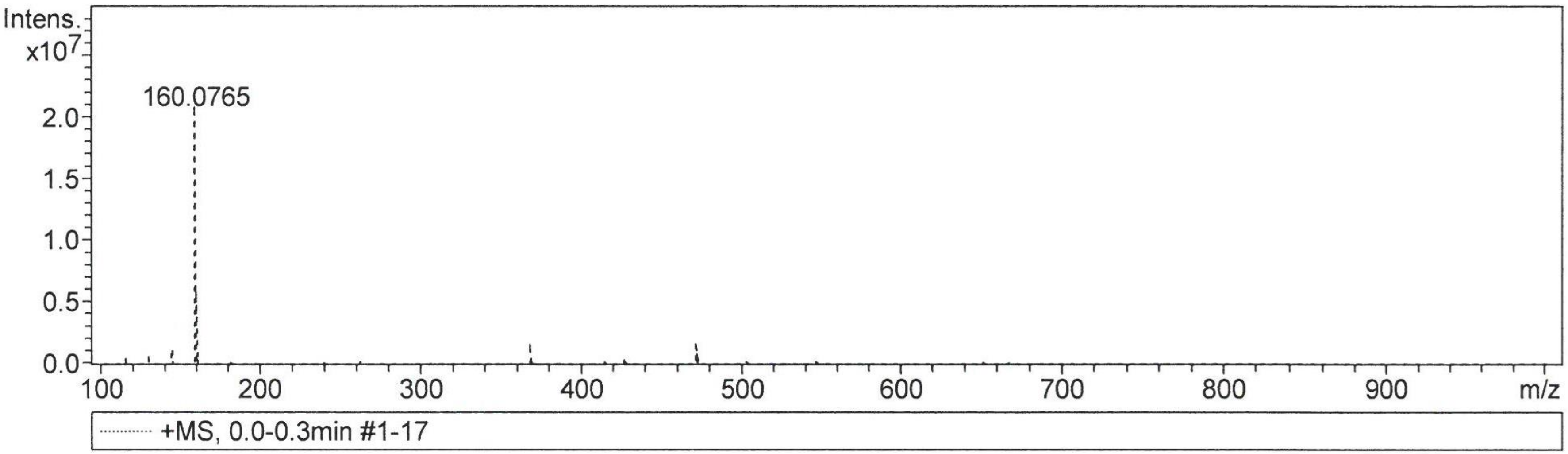

#	m/z	Res.	S/N	I	I %	FWHM
1	160.0765	11111	239307.3	20377596	100.0	0.0144

Figure S24. HR-MS spectrum of 8-methoxyquinoline (**4**).

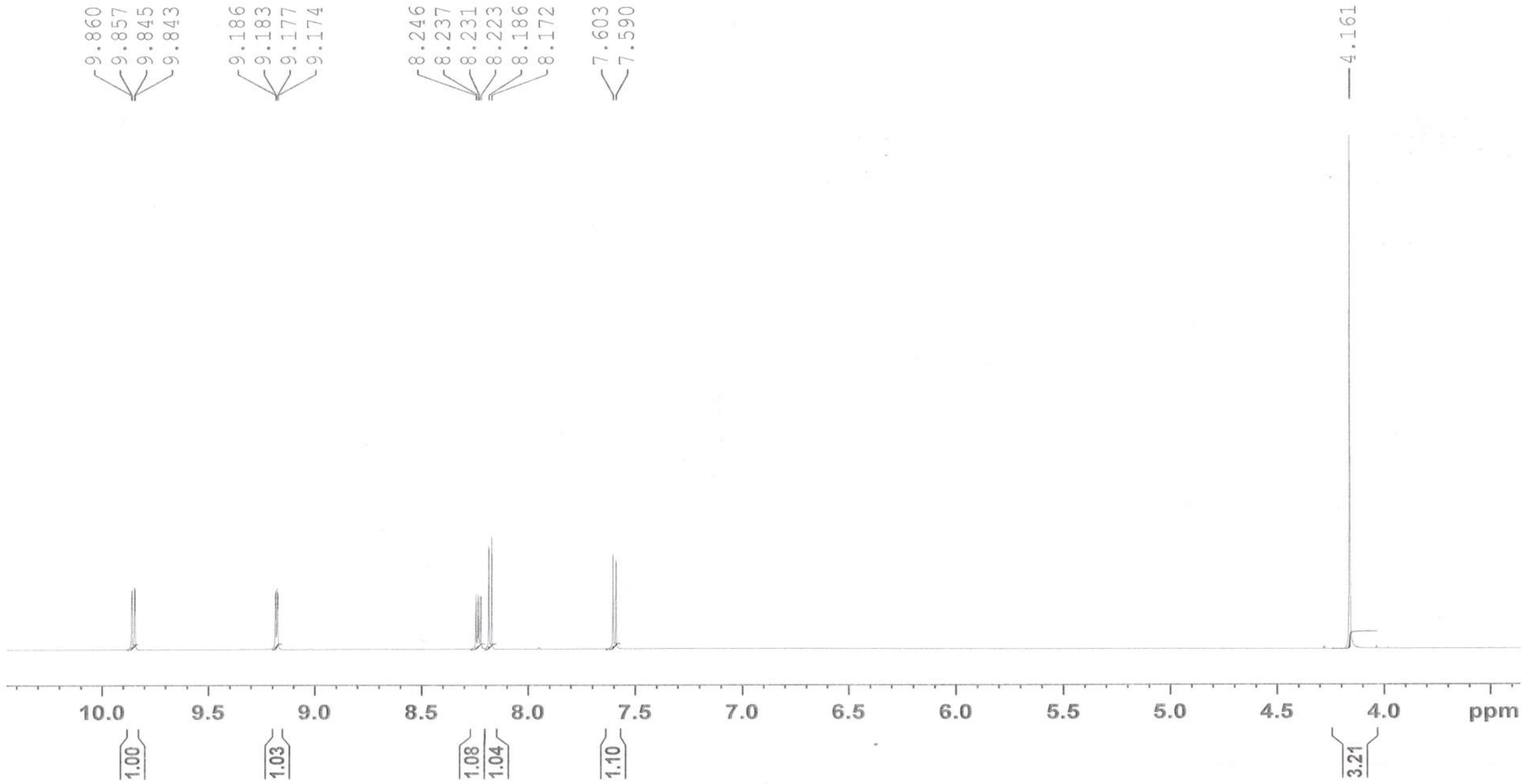

Figure S25. ^{1}H NMR spectrum of 8-methoxyquinoline-5-sulfochloride (**5**) in DMSO.

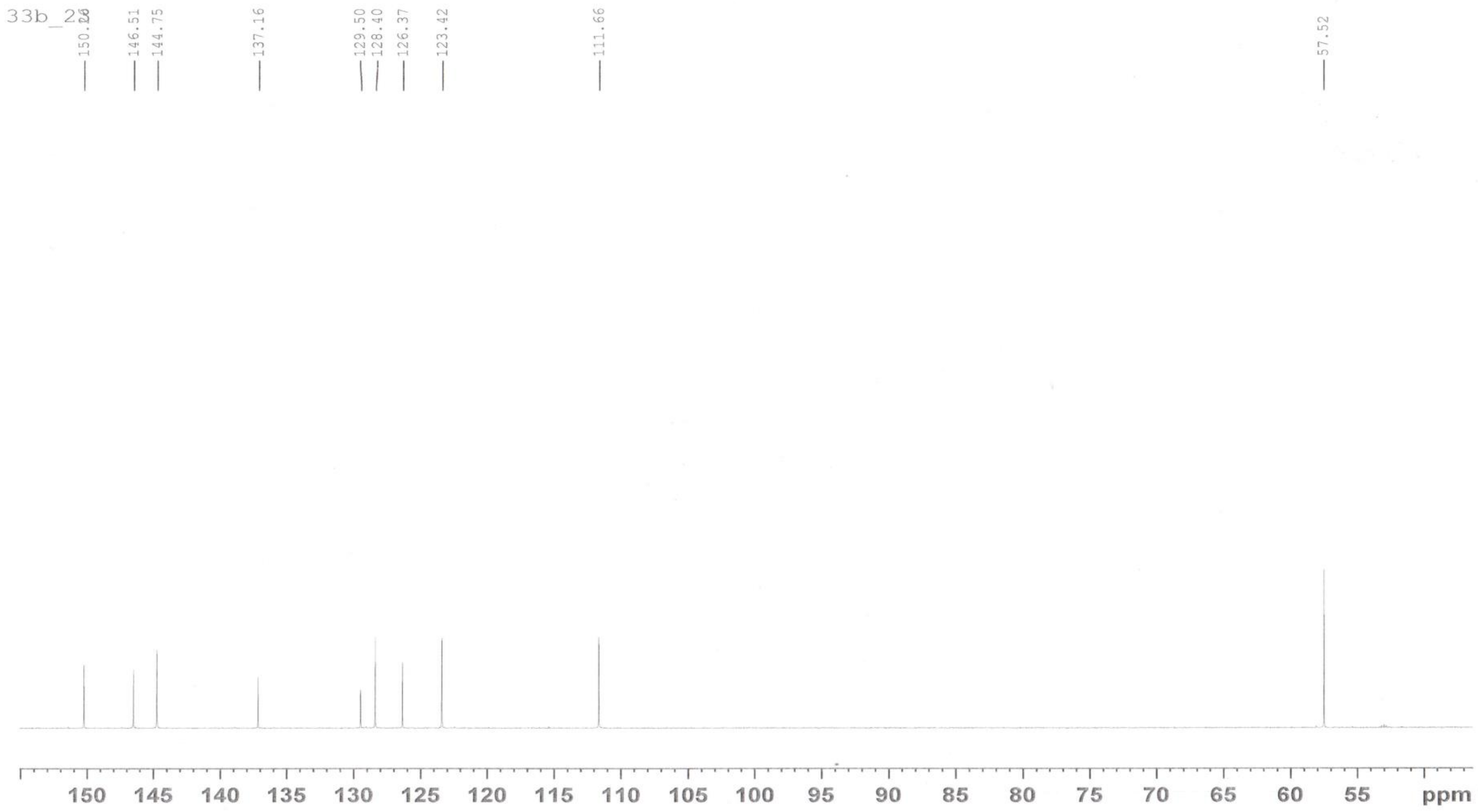

Figure S26. ^{13}C NMR spectrum of 8-methoxyquinoline-5-sulfochloride (**5**) in DMSO.

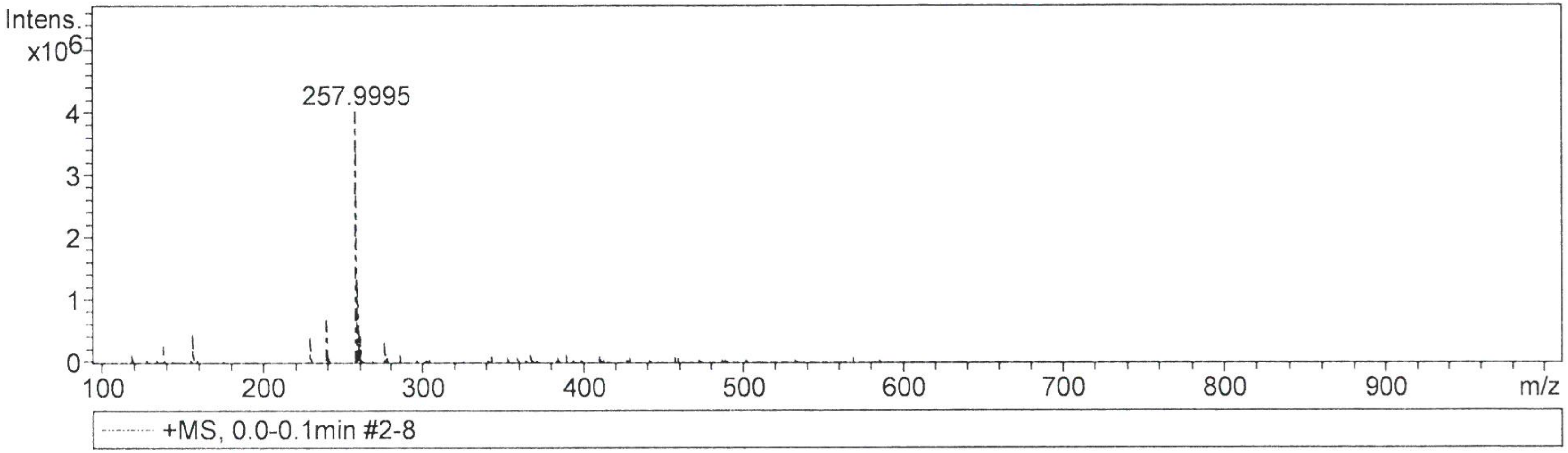

#	m/z	Res.	S/N	I	I %	FWHM
1	257.9995	33942	18527.1	4016609	100.0	0.0076
2	259.9967	27822	6068.4	1322666	32.9	0.0093

Figure S27. HR-MS spectrum of 8-methoxyquinoline-5-sulfochloride (**5**).

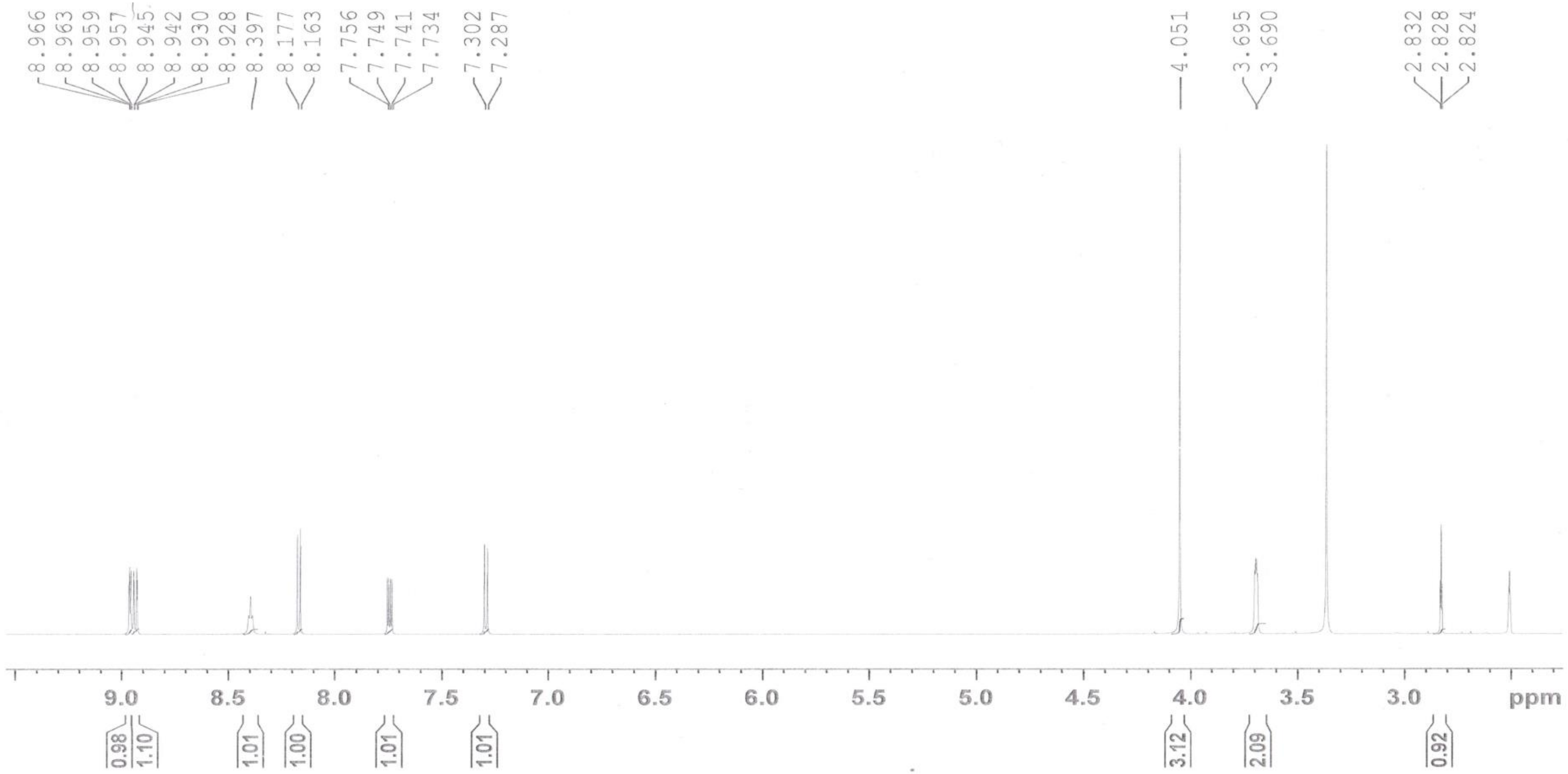

Figure S28. ^{1}H NMR spectrum of 8-methoxy-*N*-(prop-2-yn-1-yl)quinoline-5-sulfonamide (**6a**) in DMSO.

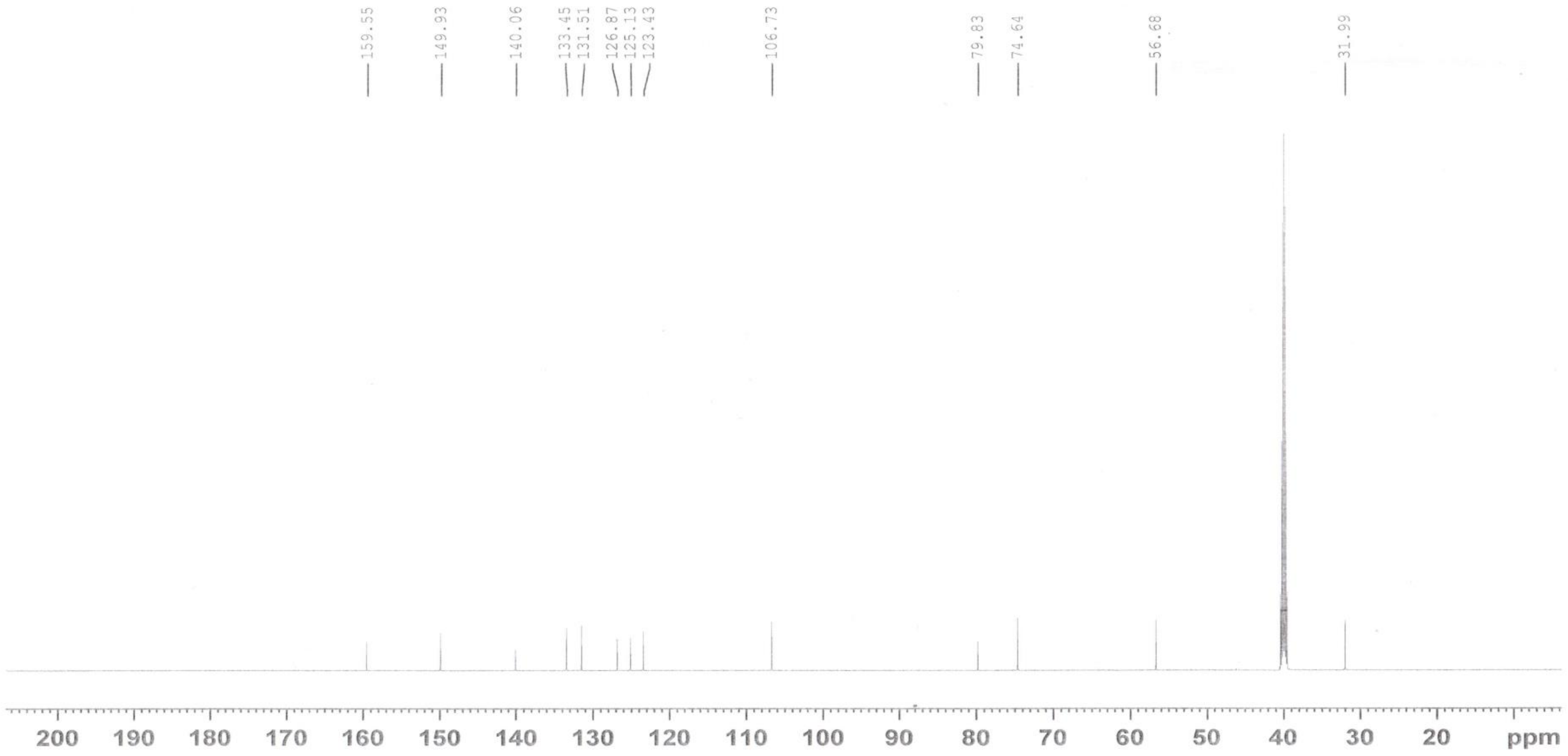

Figure S29. ^{13}C NMR spectrum of 8-methoxy-*N*-(prop-2-yn-1-yl)quinoline-5-sulfonamide (**6a**) in DMSO.

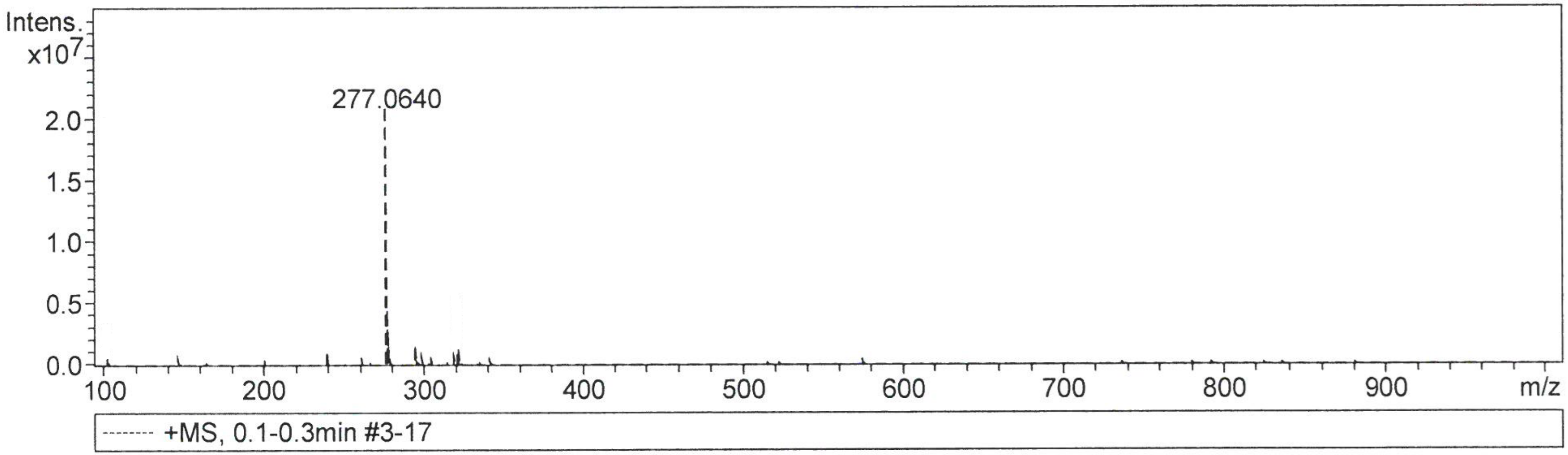

#	m/z	Res.	S/N	I	I %	FWHM
1	277.0640	17137	140917.3	20376750	100.0	0.0162

Figure S30. HR-MS spectrum of 8-methoxy-*N*-(prop-2-yn-1-yl)quinoline-5-sulfonamide (**6a**).

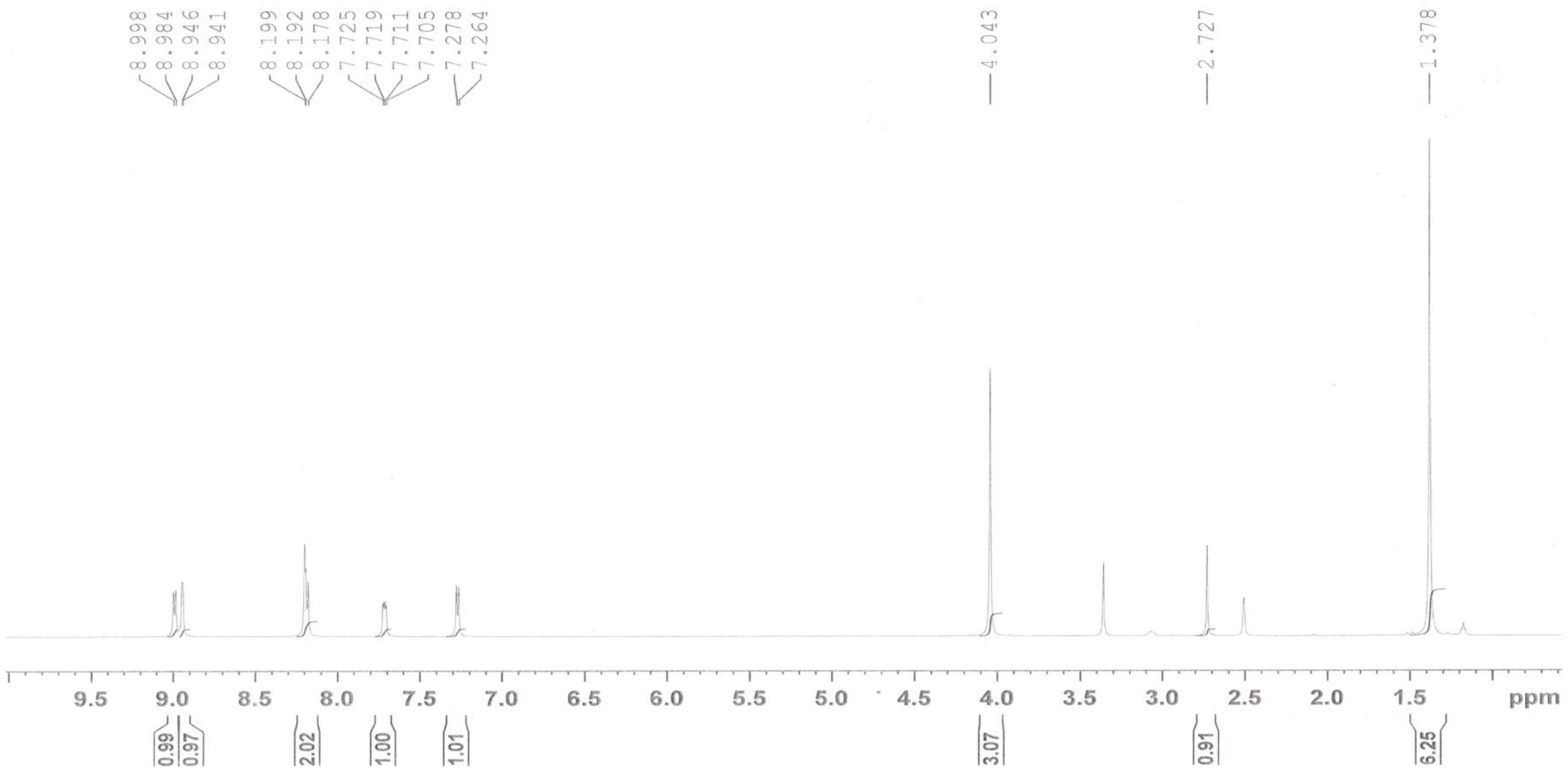

Figure S31. ^{1}H NMR spectrum of 8-methoxy-*N*-(1,1-dimethyloprop-2-yn-1-yl)quinoline-5-sulfonamide (**6b**) in DMSO.

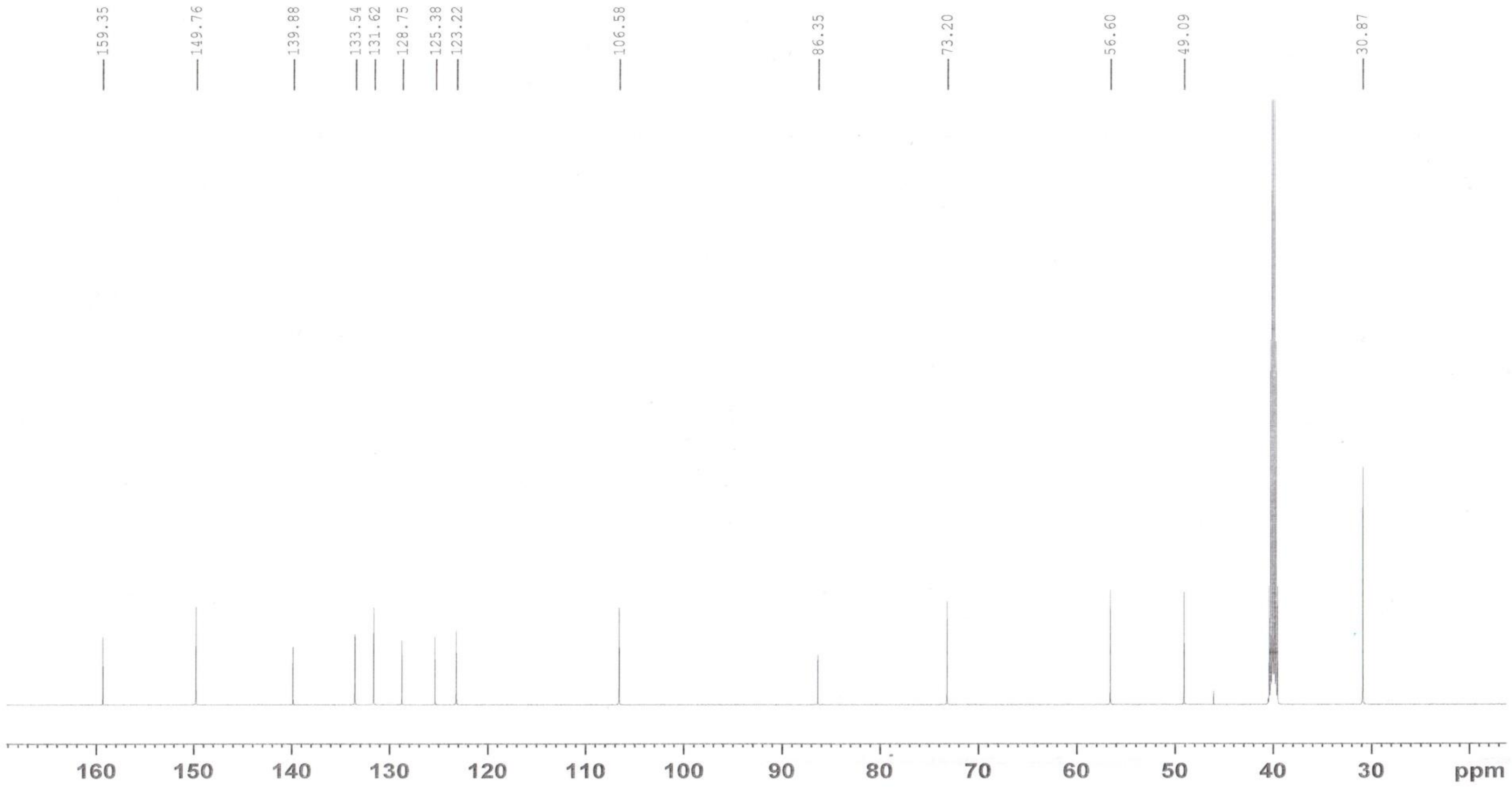

Figure S32. ^{13}C NMR spectrum of 8-methoxy-*N*-(1,1-dimethyloprop-2-yn-1-yl)quinoline-5-sulfonamide (**6b**) in DMSO.

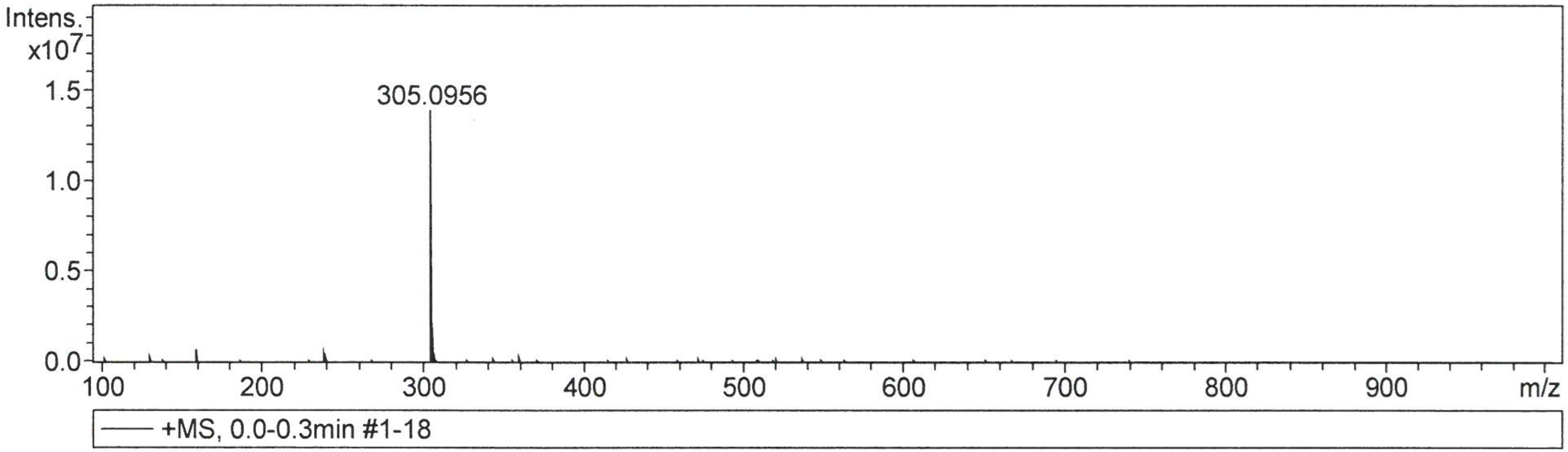

#	m/z	Res.	S/N	I	I %	FWHM
1	305.0956	38680	121467.5	13843679	100.0	0.0079

Figure S33. HR-MS spectrum of 8-methoxy-*N*-(1,1-dimethyloprop-2-yn-1-yl)quinoline-5-sulfonamide (**6b**).

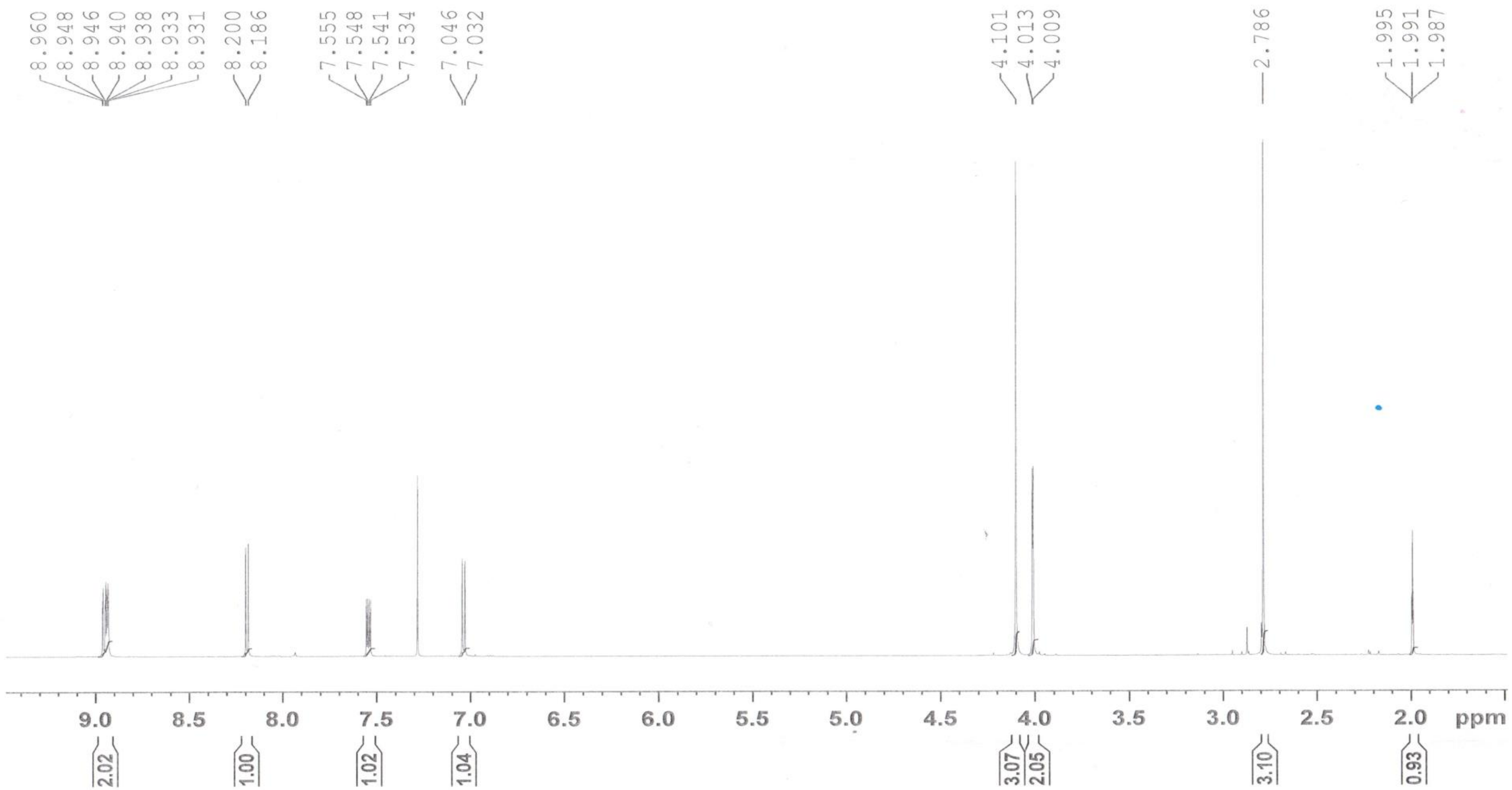

Figure S34. ^{1}H NMR spectrum of 8-methoxy-*N*-methyl-*N*-(prop-2-yn-1-yl)quinoline-5-sulfonamide (**6c**) in $CDCl_3$.

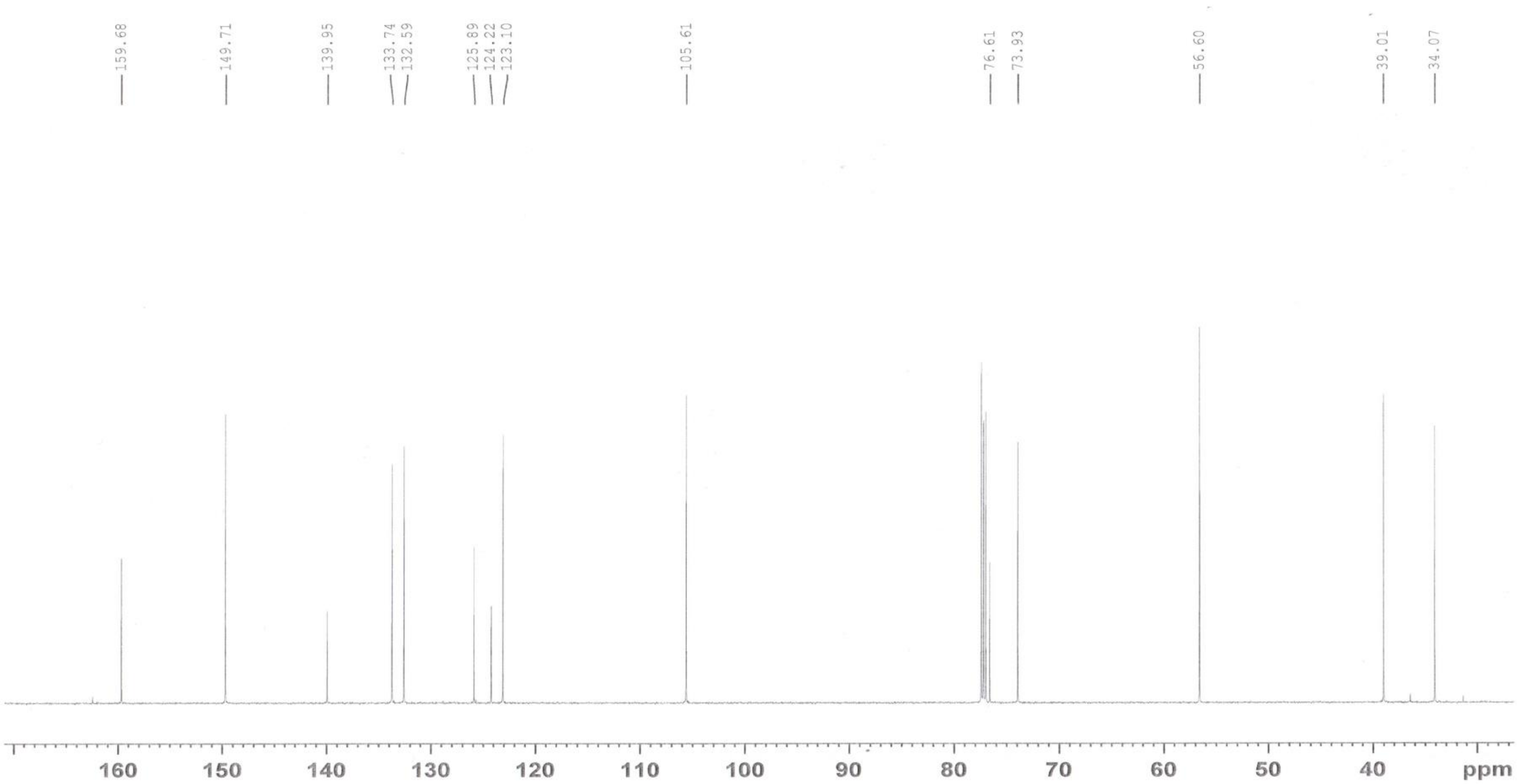

Figure S35. ^{13}C NMR spectrum of 8-methoxy-*N*-methyl-*N*-(prop-2-yn-1-yl)quinoline-5-sulfonamide (**6c**) in $CDCl_3$.

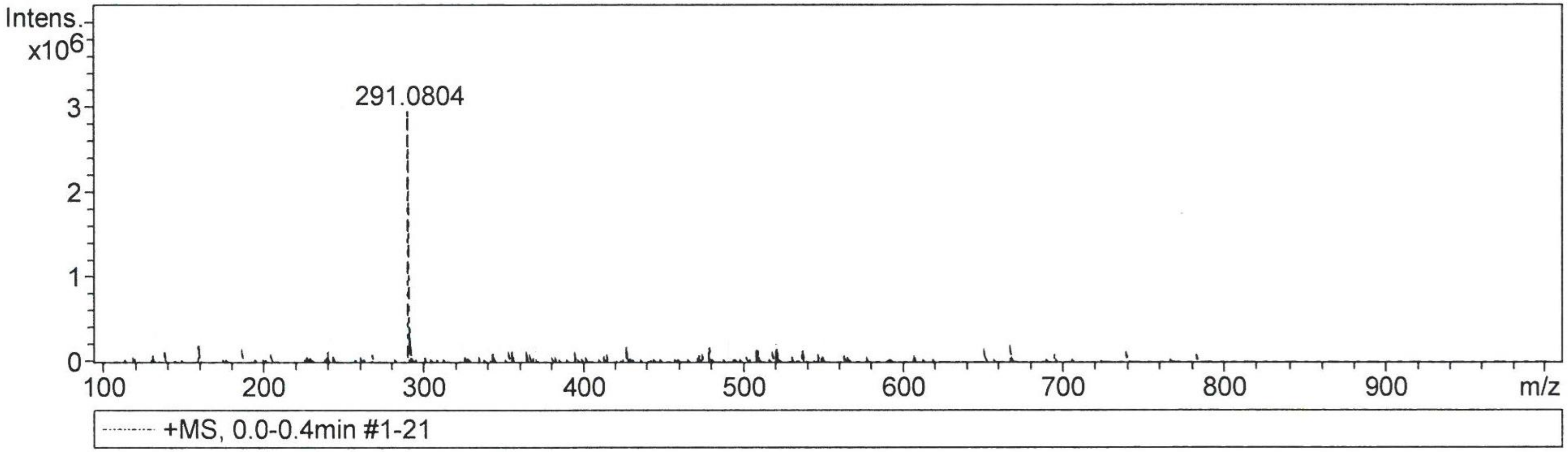

#	m/z	Res.	S/N	I	I %	FWHM
1	291.0804	34357	25900.4	2956751	100.0	0.0085

Figure S36. HR-MS spectrum of 8-methoxy-*N*-methyl-*N*-(prop-2-yn-1-yl)quinoline-5-sulfonamide (**6c**).

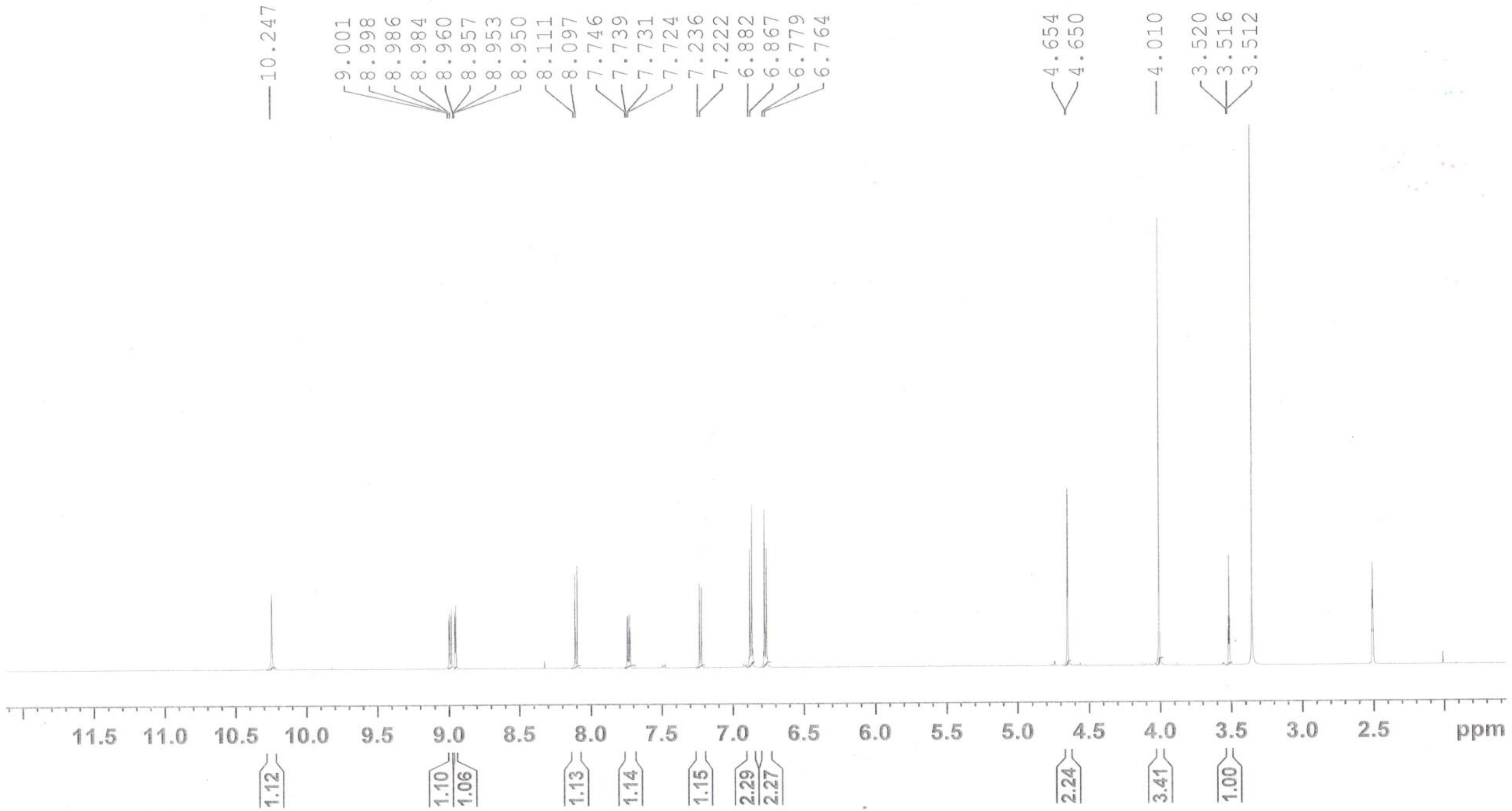

Figure S37. ^{1}H NMR spectrum of 8-methoxy-*N*-[4-(prop-2-yn-1-yloxy)phenyl]quinoline-5-sulfonamide (**6d**) in DMSO.

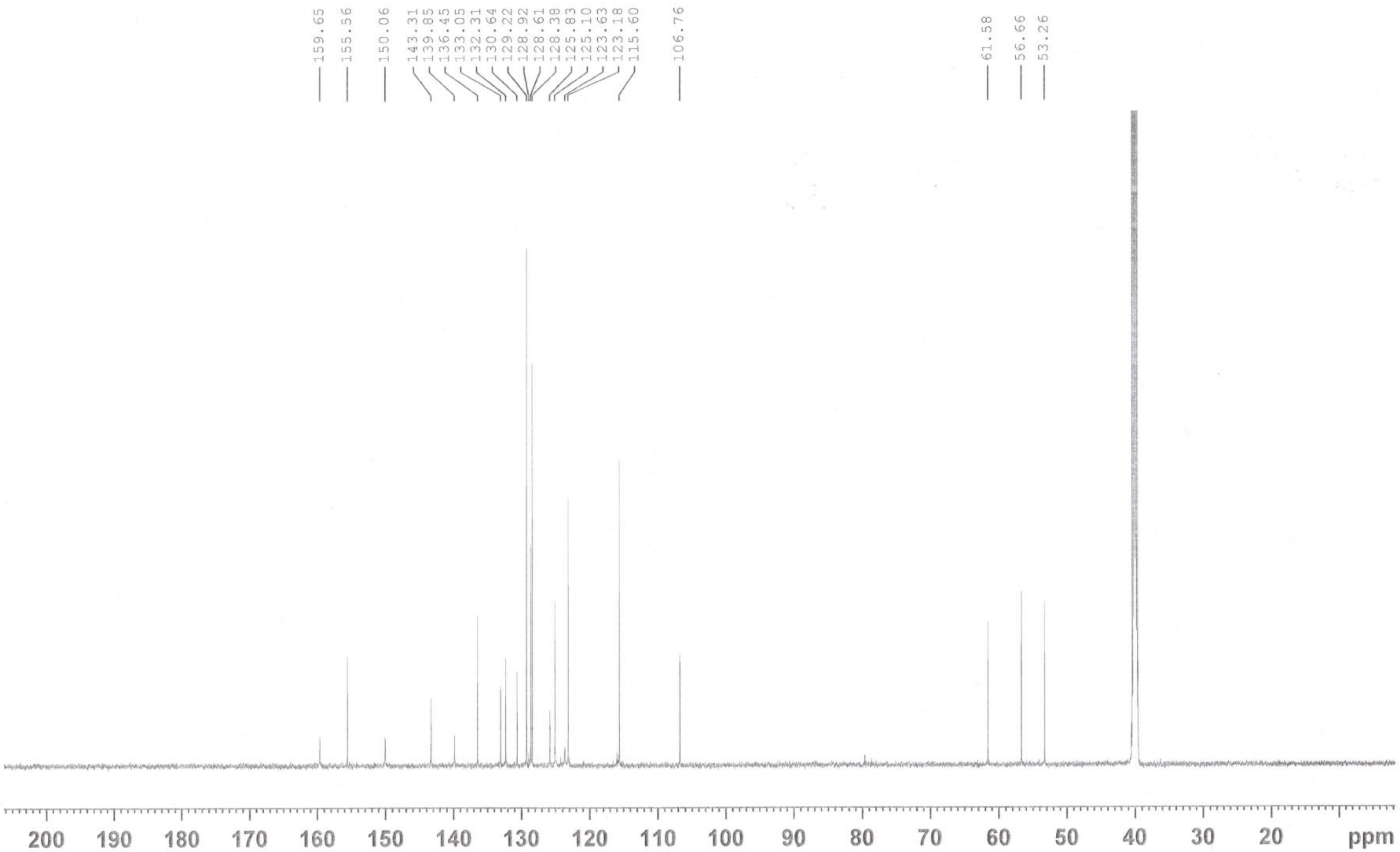

Figure S38. ^{13}C NMR spectrum of 8-methoxy-*N*-[4-(prop-2-yn-1-yloxy)phenyl]quinoline-5-sulfonamide (**6d**) in DMSO.

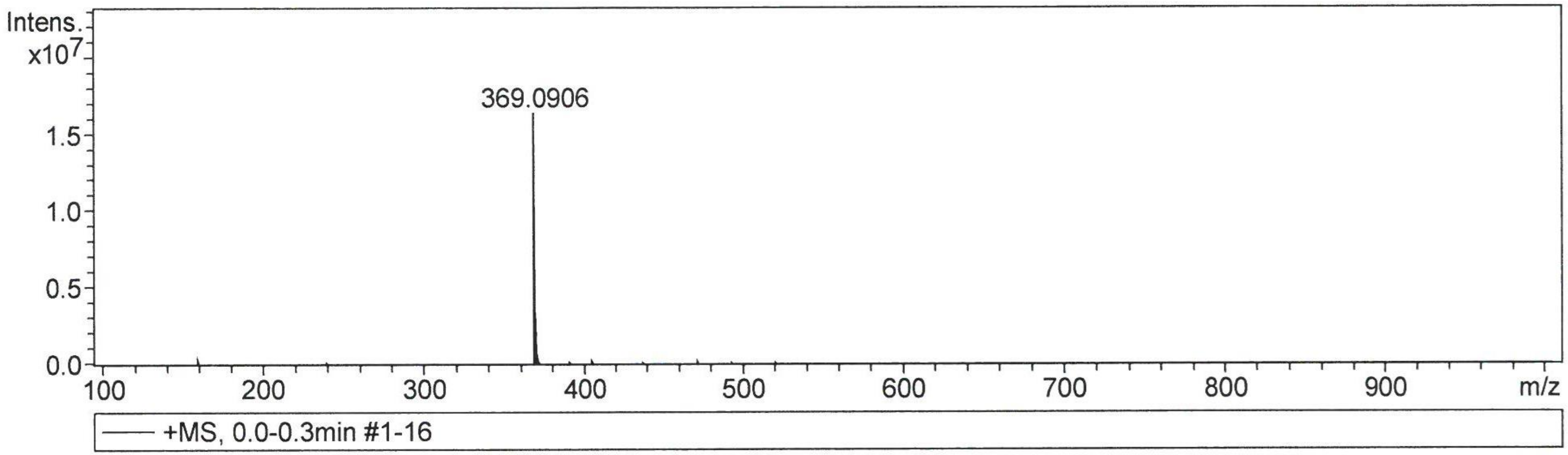

#	m/z	Res.	S/N	I	I %	FWHM
1	369.0906	40764	59840.7	16310810	100.0	0.0091

Figure S39. HR-MS spectrum of 8-methoxy-*N*-[4-(prop-2-yn-1-yloxy)phenyl]quinoline-5-sulfonamide (**6d**).

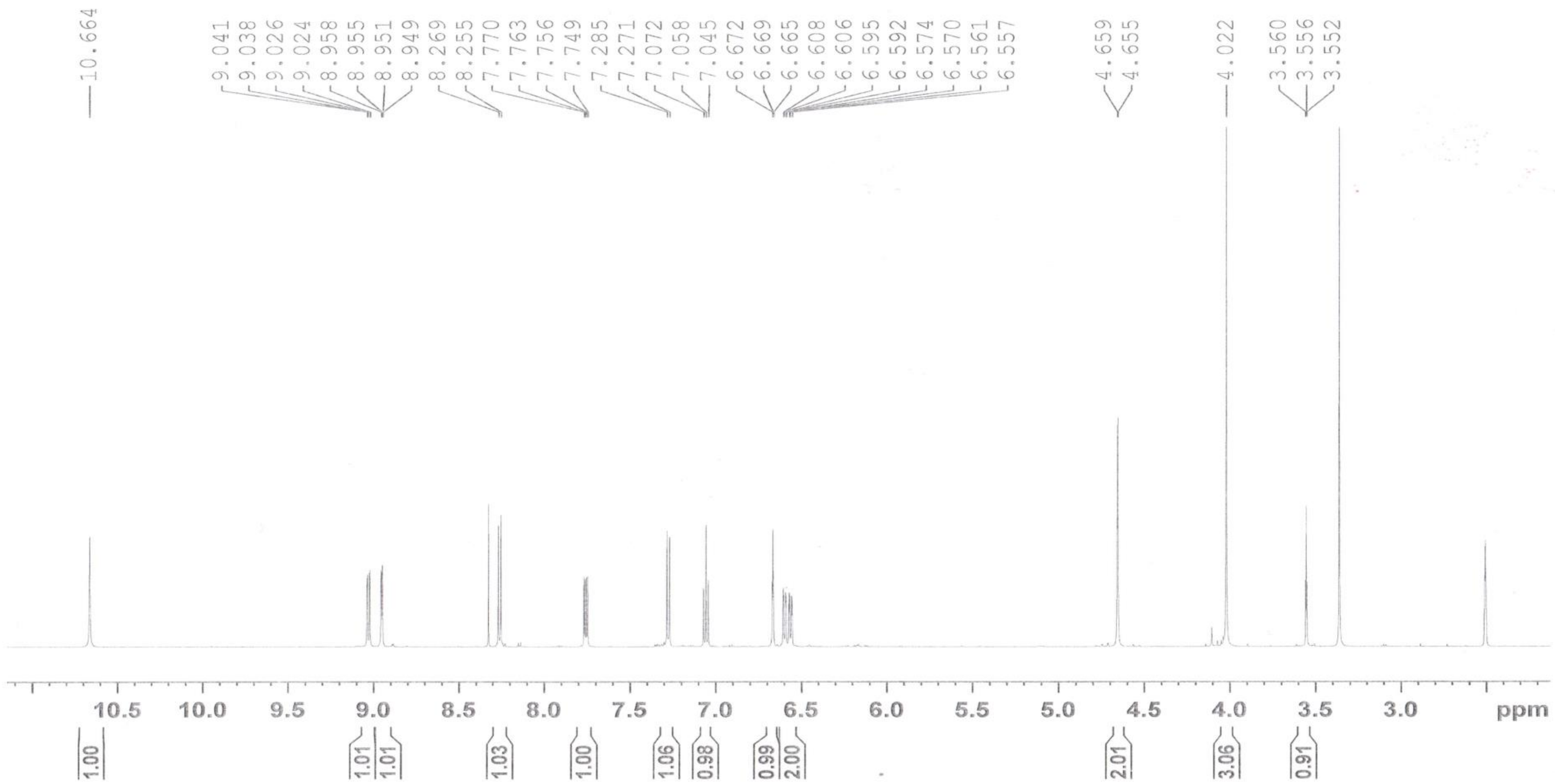

Figure S40. ^{1}H NMR spectrum of 8-methoxy-*N*-[3-(prop-2-yn-1-yloxy)phenyl]quinoline-5-sulfonamide (**6e**) in DMSO.

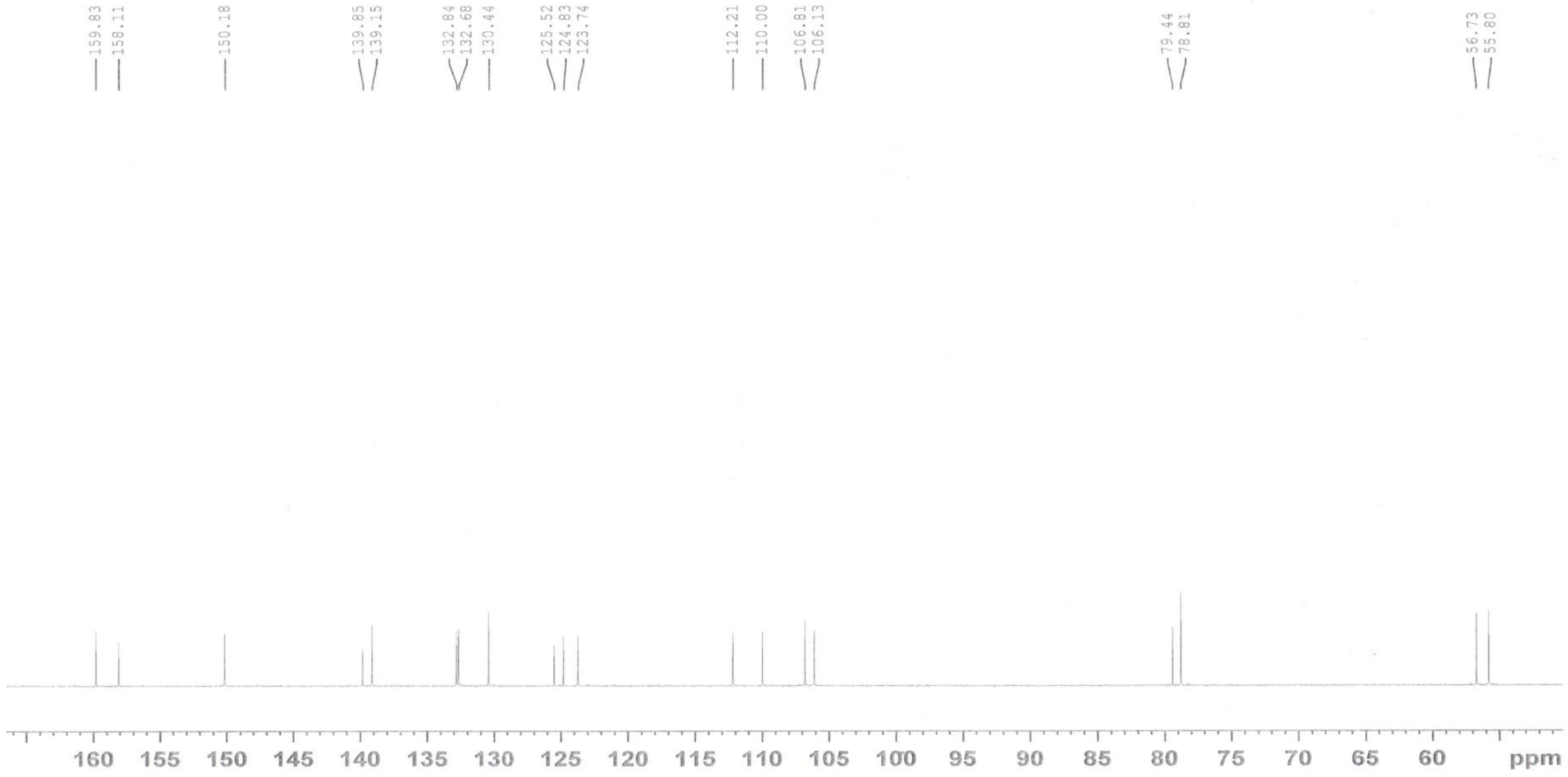

Figure S41. ^{13}C NMR spectrum of 8-methoxy-*N*-[3-(prop-2-yn-1-yloxy)phenyl]quinoline-5-sulfonamide (**6e**) in DMSO.

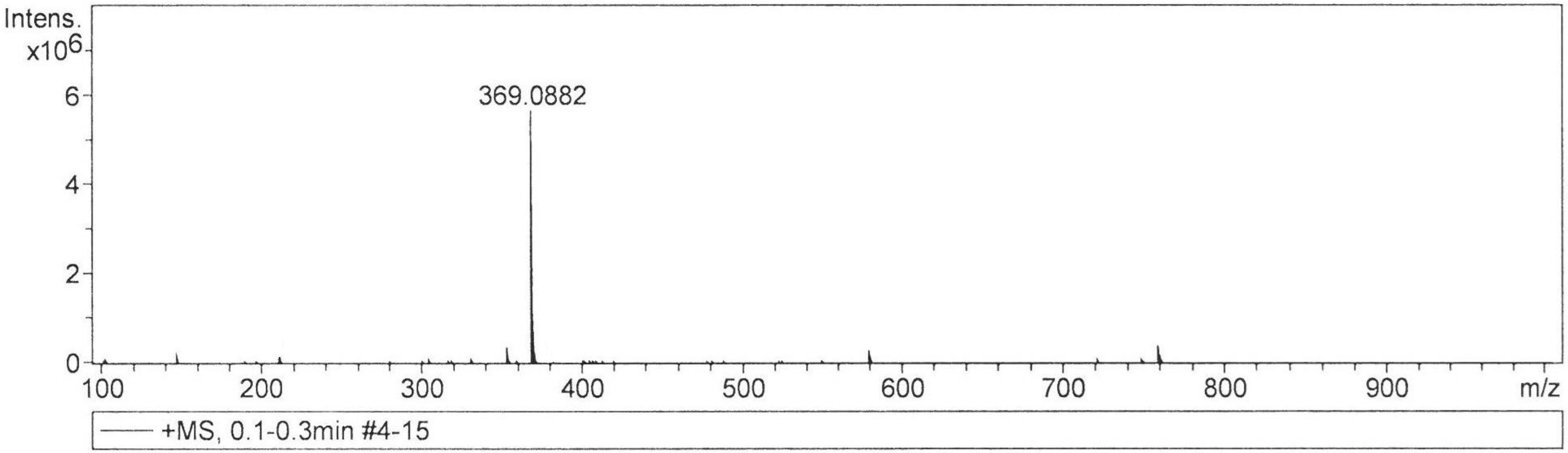

#	m/z	Res.	S/N	I	I %	FWHM
1	369.0882	39576	123581.4	5637293	100.0	0.0093

Figure S42. HR-MS spectrum of 8-methoxy-*N*-[3-(prop-2-yn-1-yloxy)phenyl]quinoline-5-sulfonamide (**6e**).

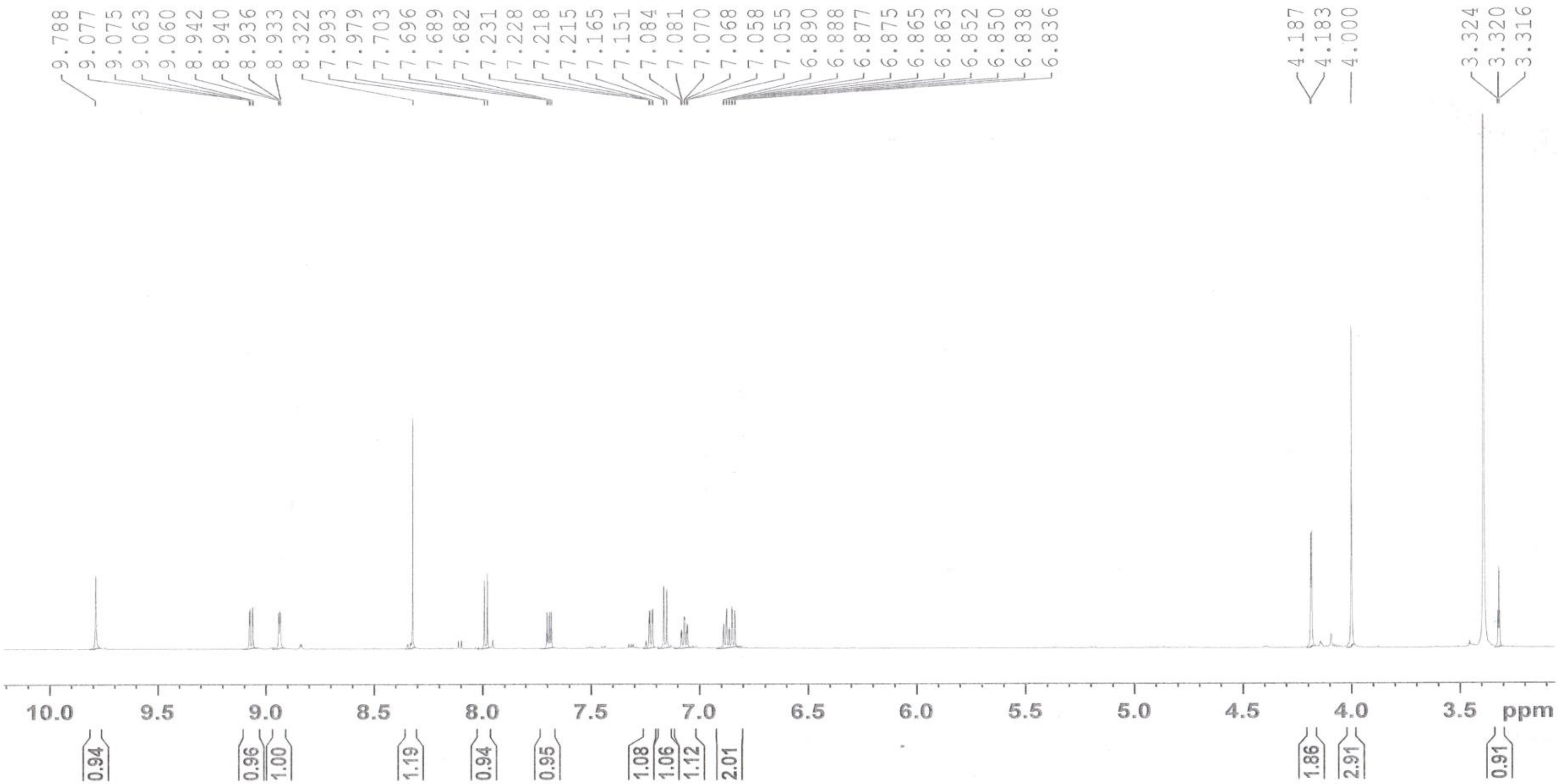

Figure S43. ^{1}H NMR spectrum of 8-methoxy-*N*-[2-(prop-2-yn-1-yloxy)phenyl]quinoline-5-sulfonamide (**6f**) in DMSO.

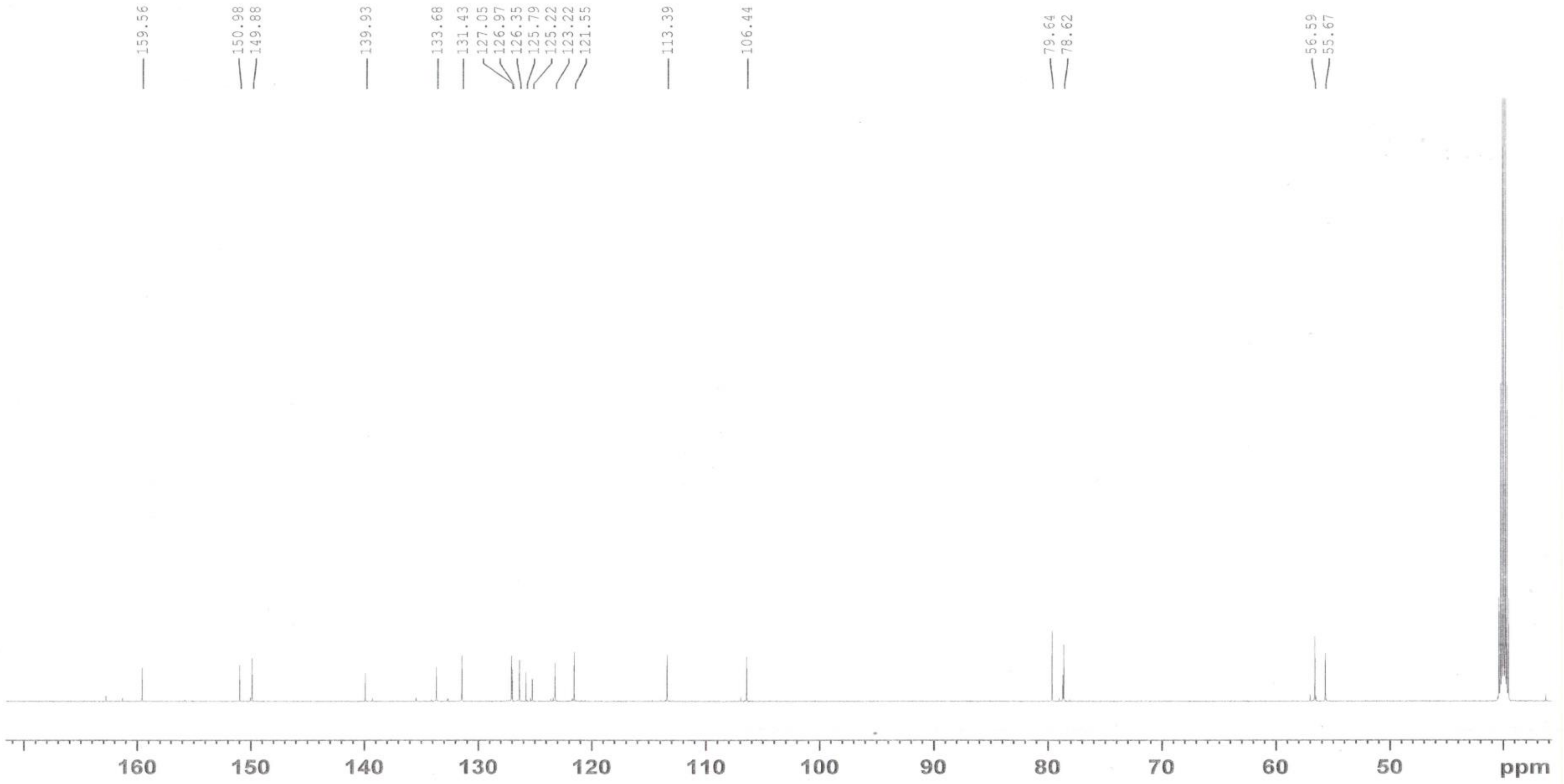

Figure S44. ^{13}C NMR spectrum of 8-methoxy-*N*-[2-(prop-2-yn-1-yloxy)phenyl]quinoline-5-sulfonamide (**6f**) in DMSO.

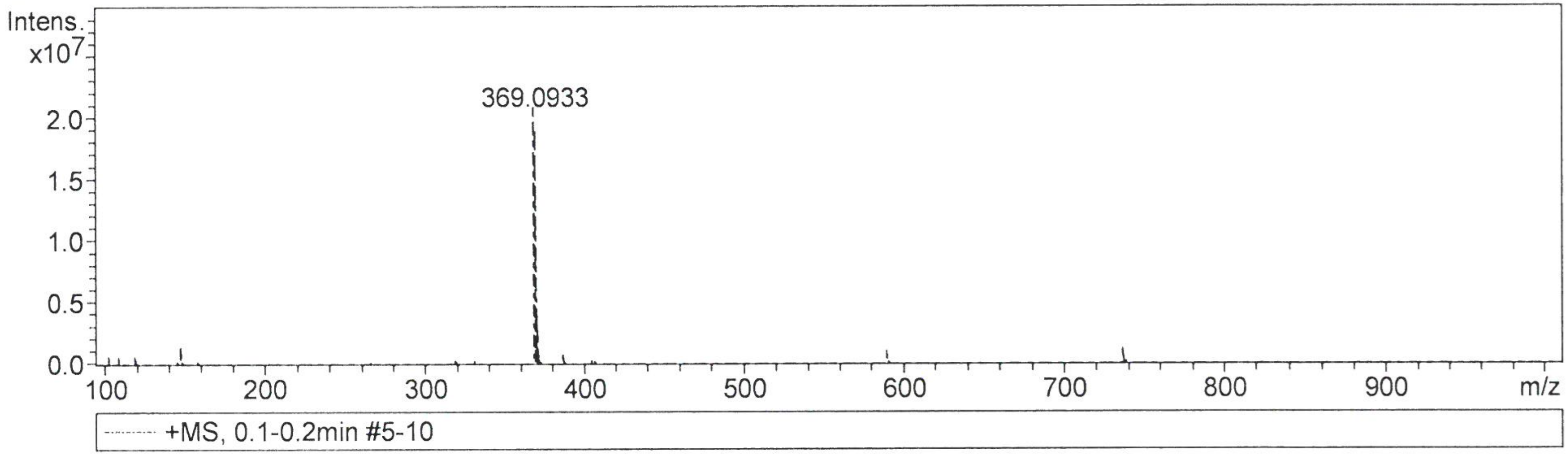

#	m/z	Res.	S/N	I	I %	FWHM
1	369.0933	9206	151959.8	20377596	100.0	0.0401

Figure S45. HR-MS spectrum of 8-methoxy-*N*-[2-(prop-2-yn-1-yloxy)phenyl]quinoline-5-sulfonamide (**6f**).

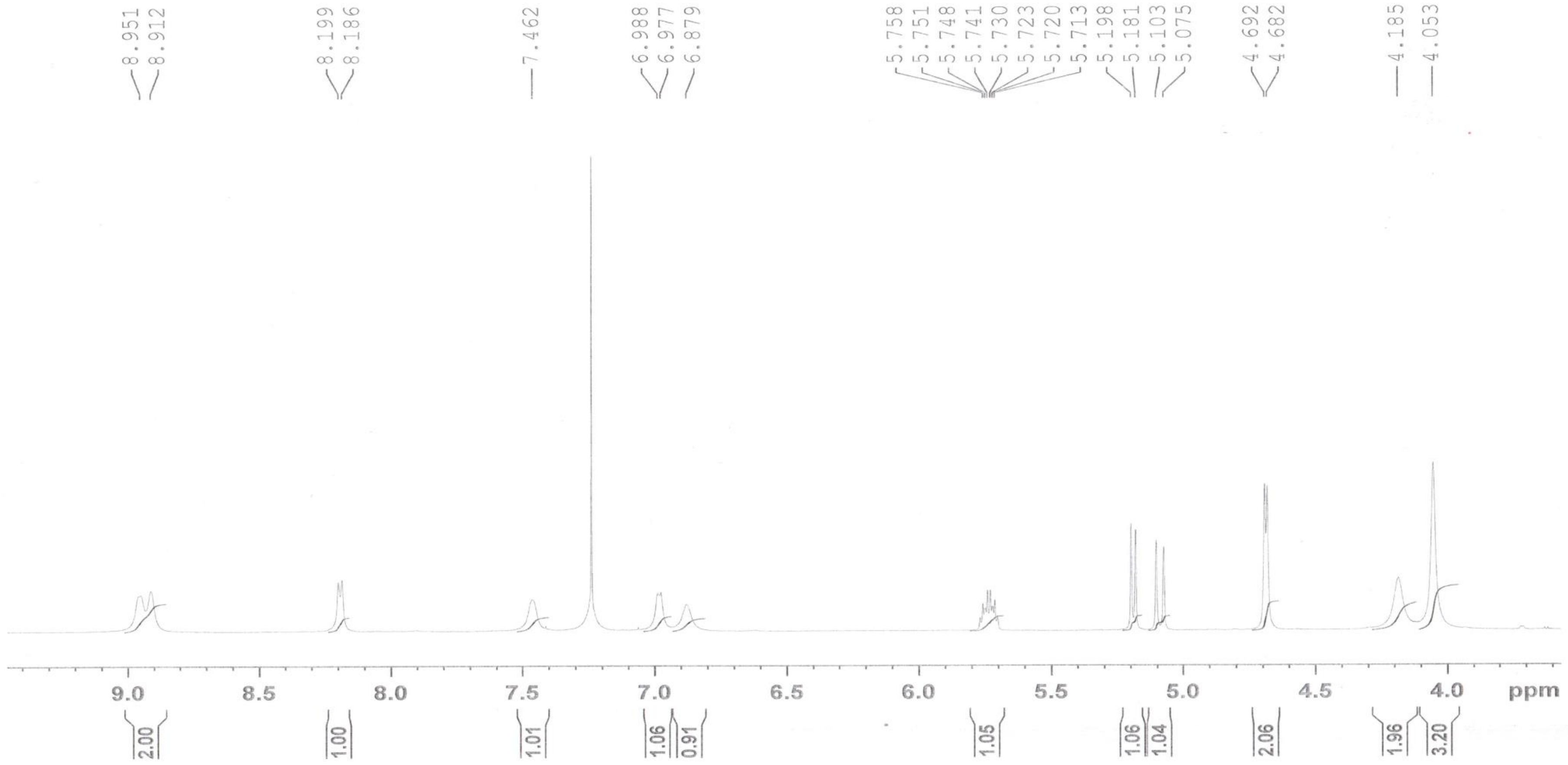

Figure S46. ^{1}H NMR spectrum of *N*-[(1-allyl-1*H*-1,2,3-triazol-4-yl)methyl]-8-methoxyquinoline-5-sulfonamide (**7a**) in $CDCl_3$.

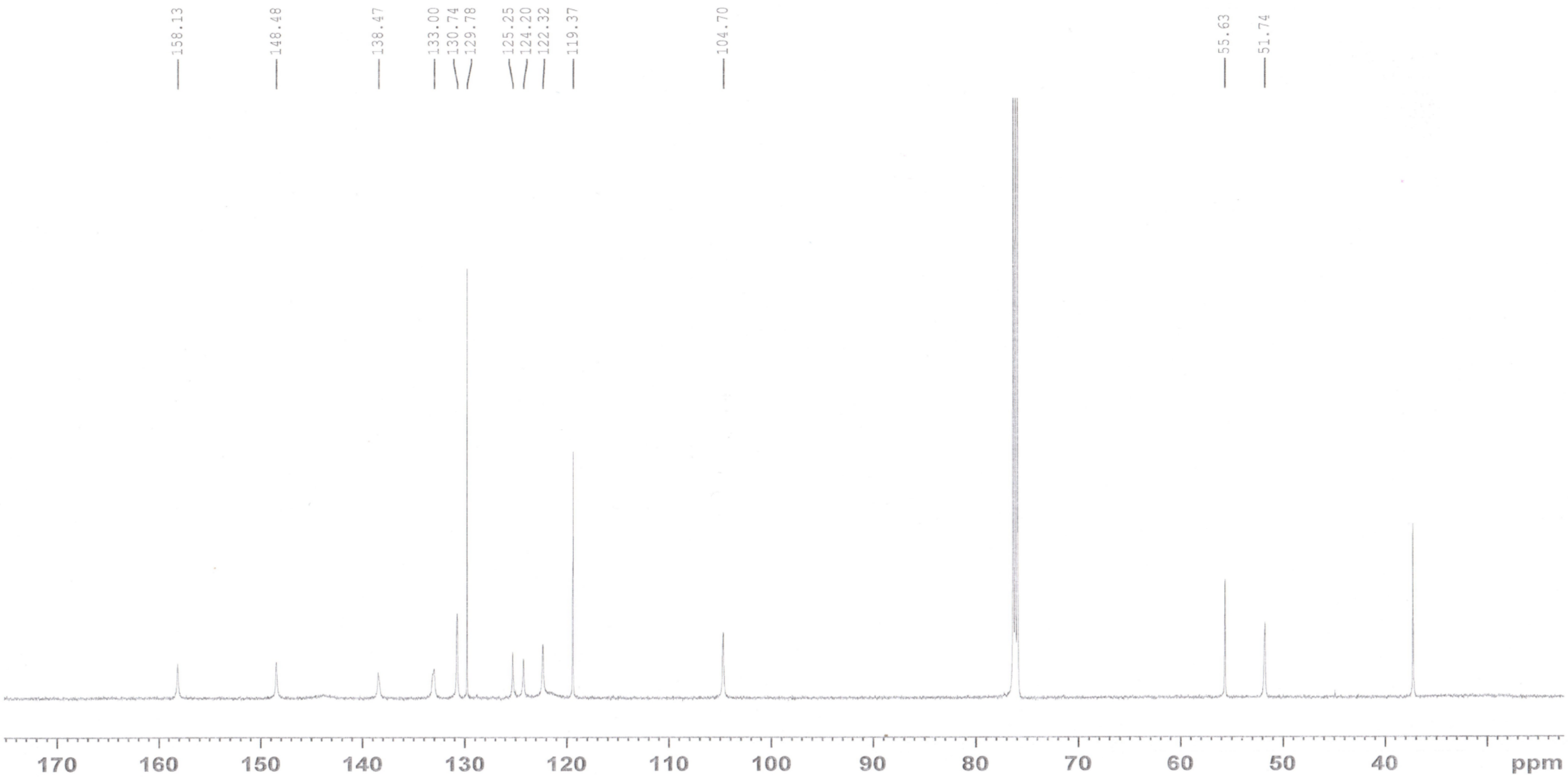

Figure S47. ^{13}C NMR spectrum of *N*-[(1-allyl-1*H*-1,2,3-triazol-4-yl)methyl]-8-methoxyquinoline-5-sulfonamide (**7a**) in $CDCl_3$.

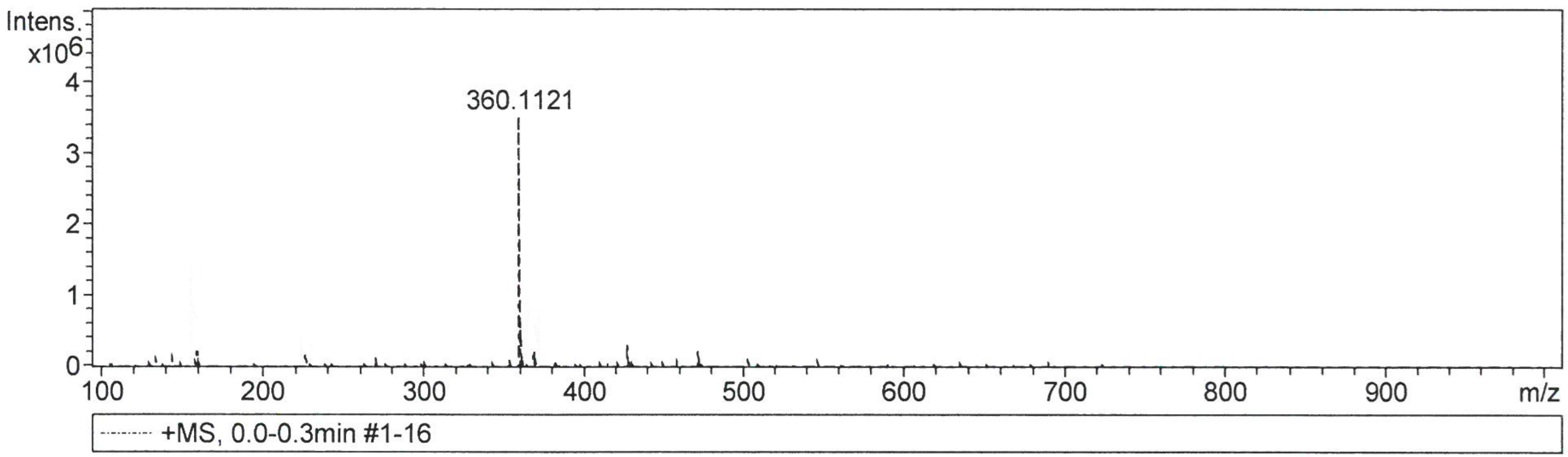

#	m/z	Res.	S/N	I	I %	FWHM
1	360.1121	37784	13952.6	3534321	100.0	0.0095

Figure S48. HR-MS spectrum of *N*-[(1-allyl-1*H*-1,2,3-triazol-4-yl)methyl]-8-methoxyquinoline-5-sulfonamide (**7a**).

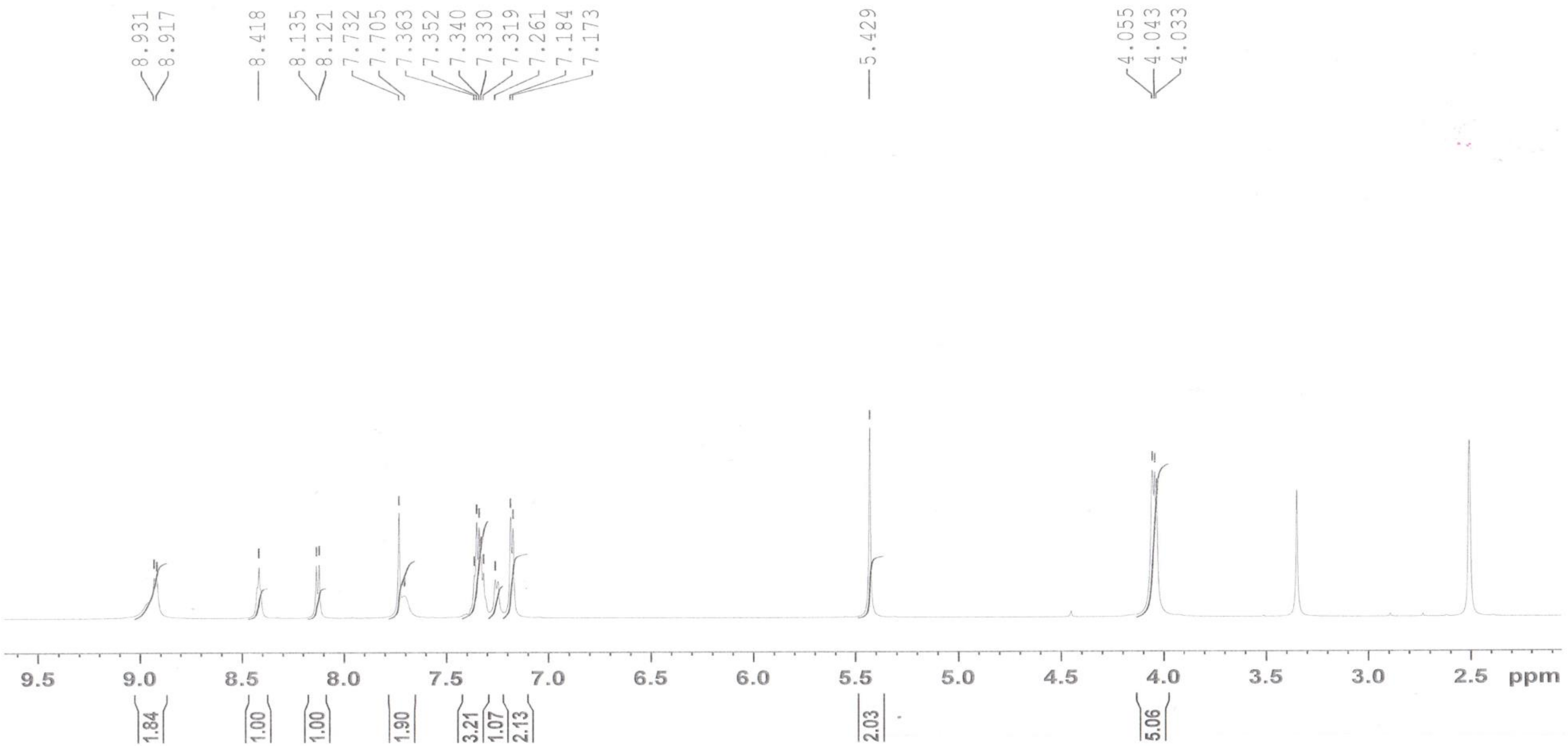

Figure S49. ^{1}H NMR spectrum of *N*-[(1-benzyl-1*H*-1,2,3-triazol-4-yl)methyl]-8-methoxyquinoline-5-sulfonamide (**7b**) in DMSO.

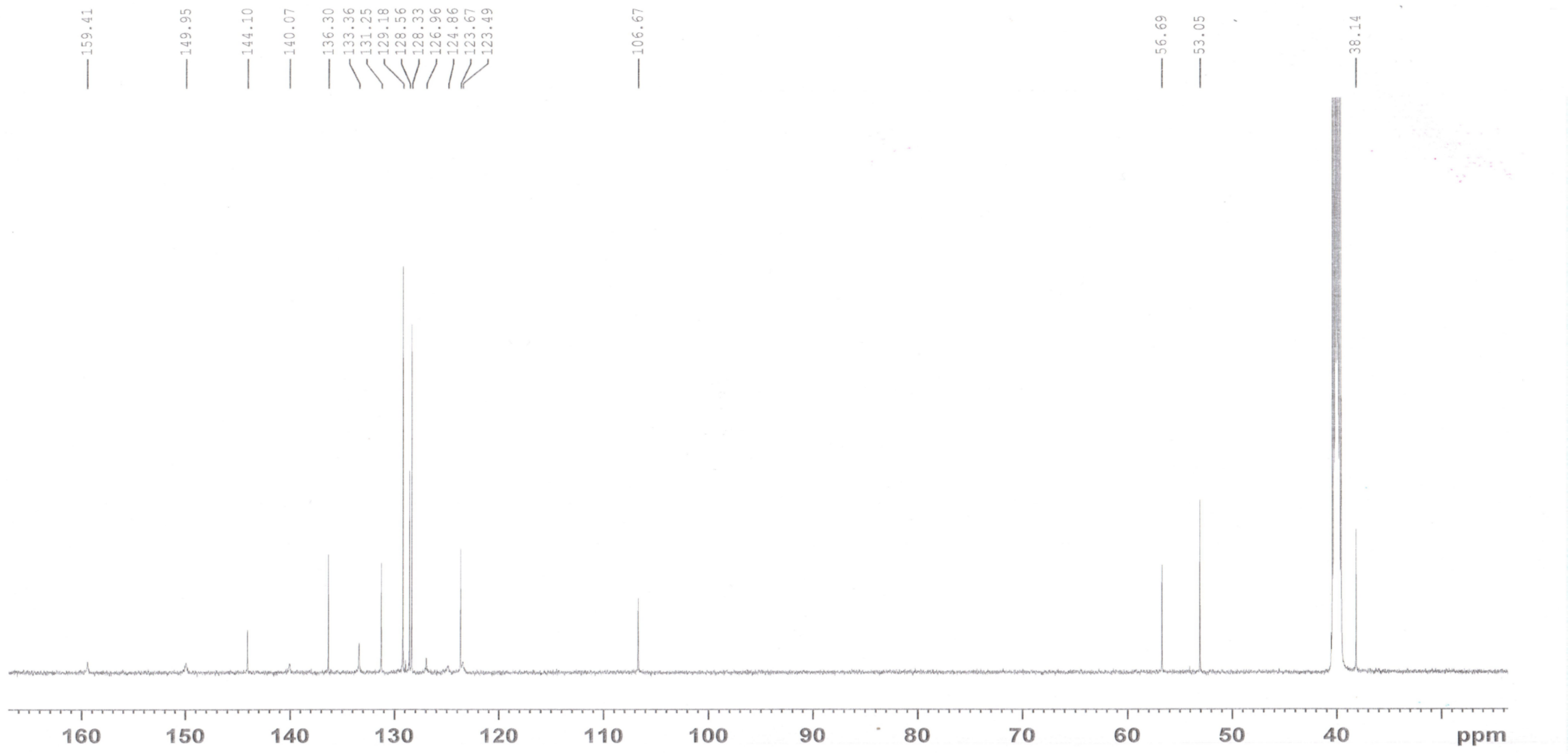

Figure S50. ^{13}C NMR spectrum of *N*-[(1-benzyl-1*H*-1,2,3-triazol-4-yl)methyl]-8-methoxyquinoline-5-sulfonamide (**7b**) in DMSO.

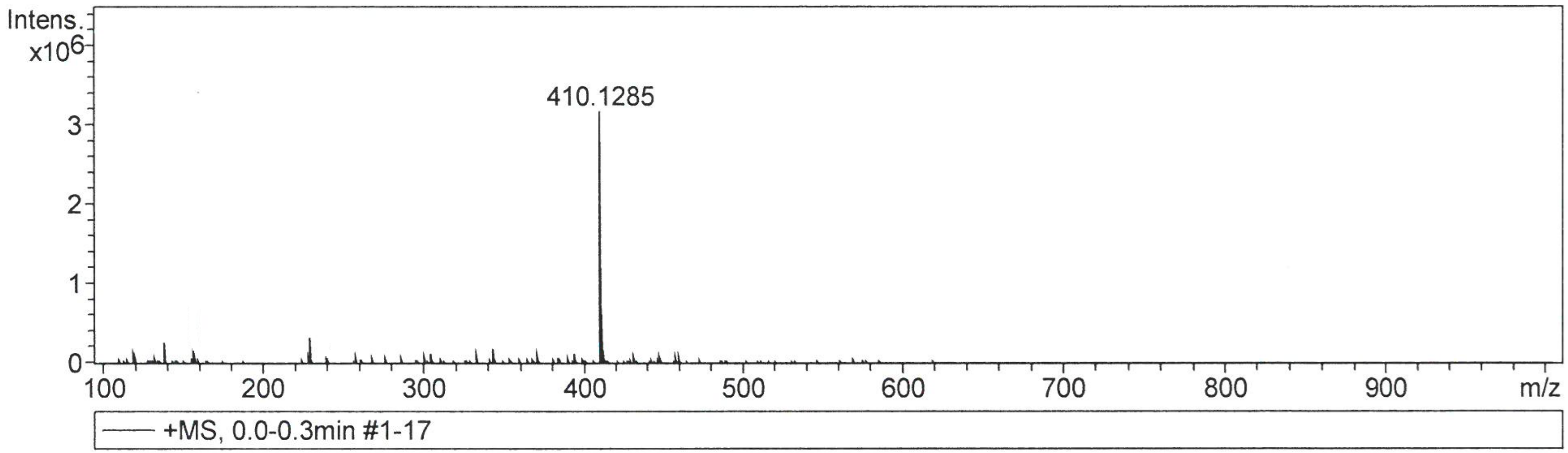

#	m/z	Res.	S/N	I	I %	FWHM
1	410.1285	38611	13658.3	3156053	100.0	0.0106

Figure S51. HR-MS spectrum of *N*-[(1-benzyl-1*H*-1,2,3-triazol-4-yl)methyl]-8-methoxyquinoline-5-sulfonamide (**7b**).

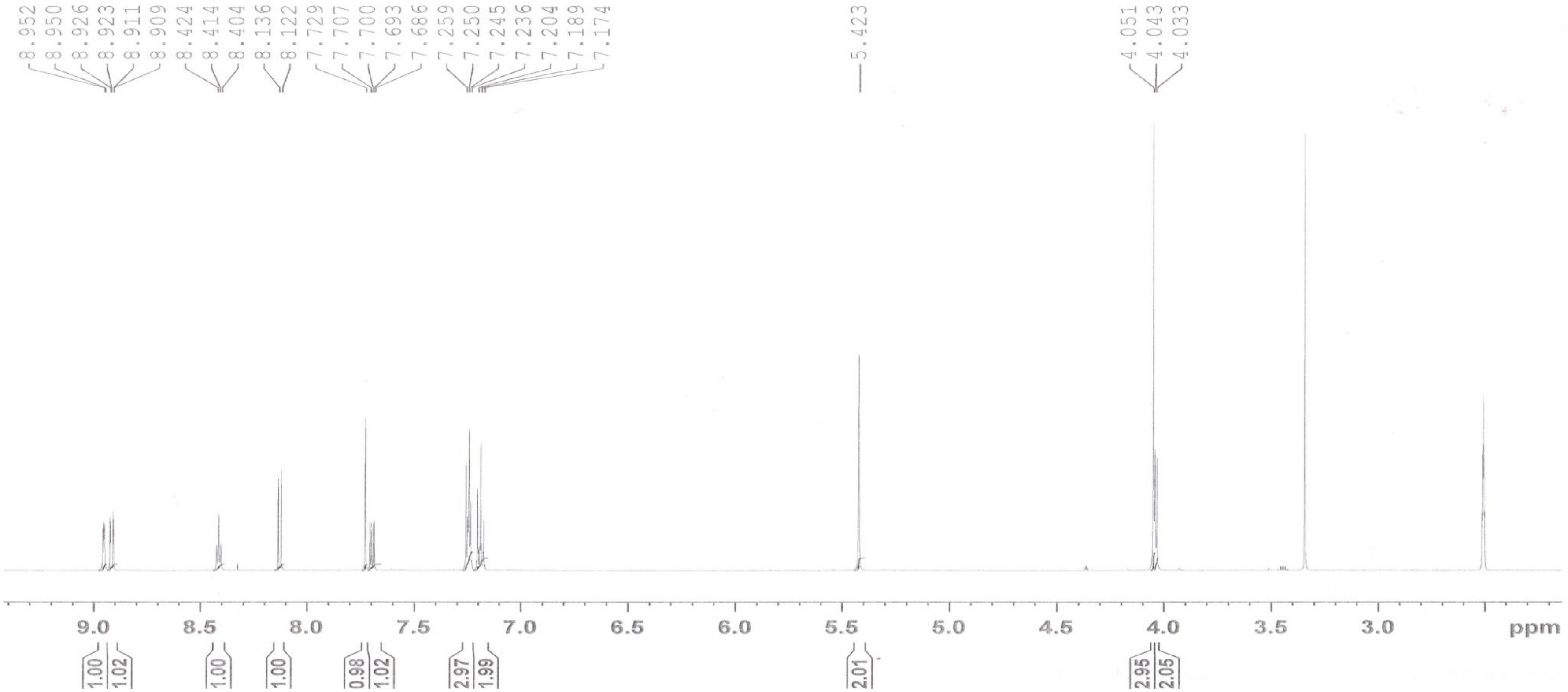

Figure S52. ^{1}H NMR spectrum of *N*-{[1-(4-fluorobenzyl)-1*H*-1,2,3-triazol-4-yl]methyl}-8-methoxyquinoline-5-sulfonamide (**7c**) in DMSO.

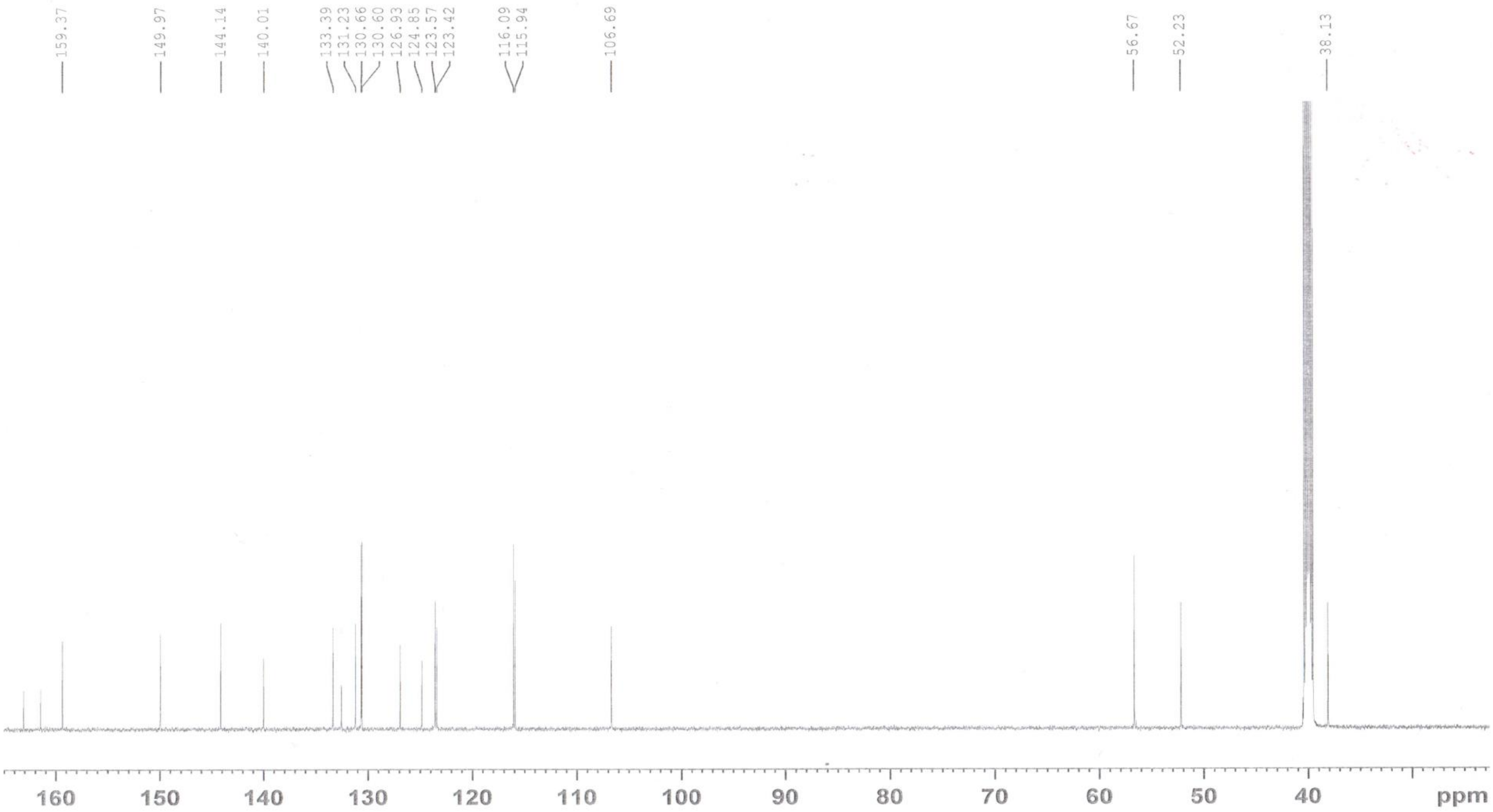

Figure S53. ^{13}C NMR spectrum of *N*-{[1-(4-fluorobenzyl)-1*H*-1,2,3-triazol-4-yl]methyl}-8-methoxyquinoline-5-sulfonamide (**7c**) in DMSO.

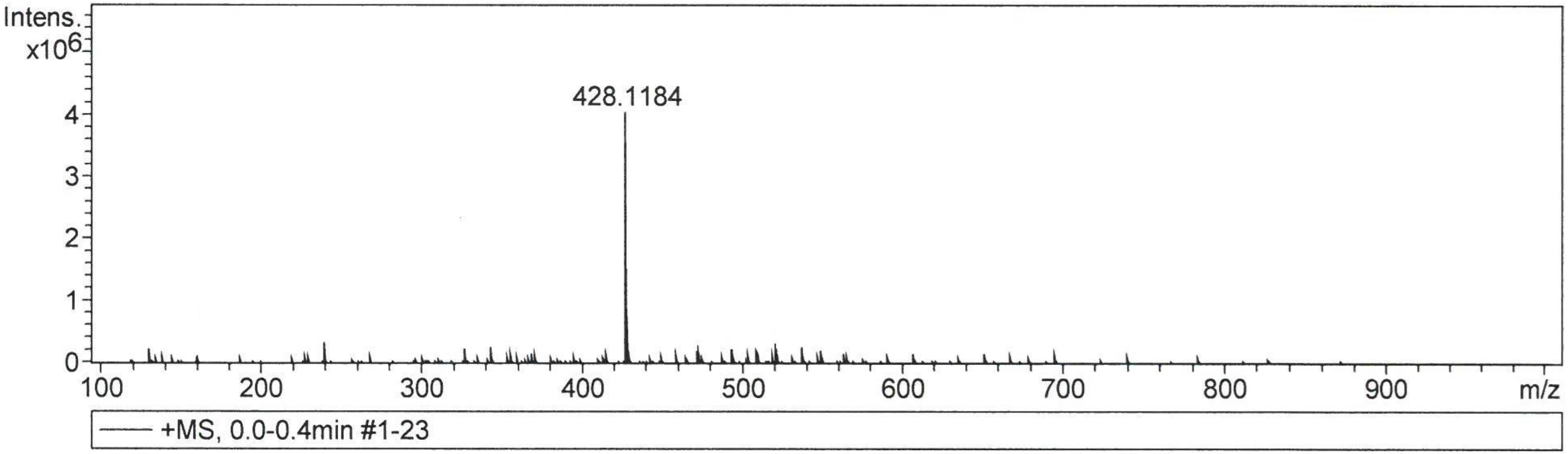

#	m/z	Res.	S/N	I	I %	FWHM
1	428.1184	40216	16210.1	4050934	100.0	0.0106

Figure S54. HR-MS spectrum of *N*-{[1-(4-fluorobenzyl)-1*H*-1,2,3-triazol-4-yl]methyl}-8-methoxyquinoline-5-sulfonamide (**7c**).

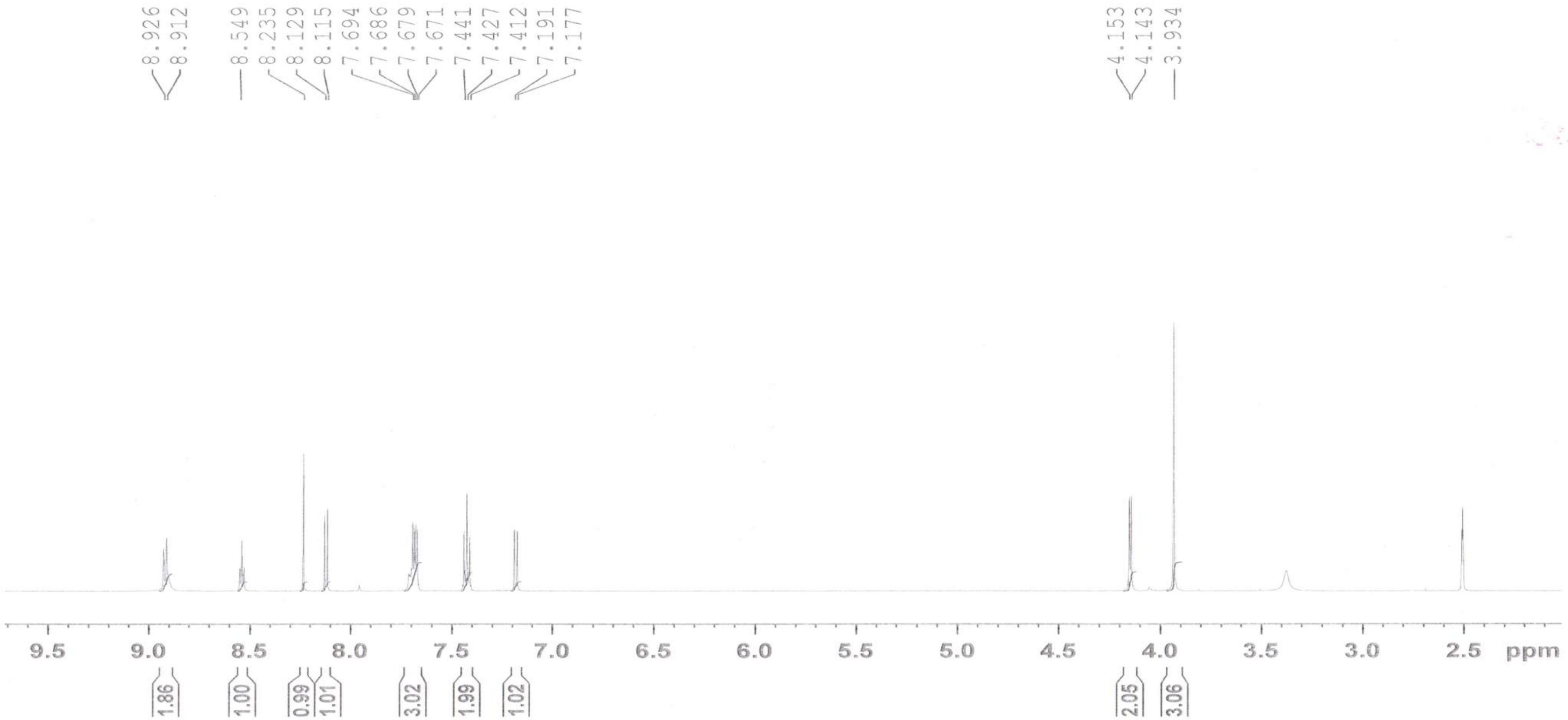

Figure S55. ^{1}H NMR spectrum of *N*-{[1-(4-chlorophenyl)-1*H*-1,2,3-triazol-4-yl]methyl}-8-methoxyquinoline-5-sulfonamide (**7d**) in DMSO.

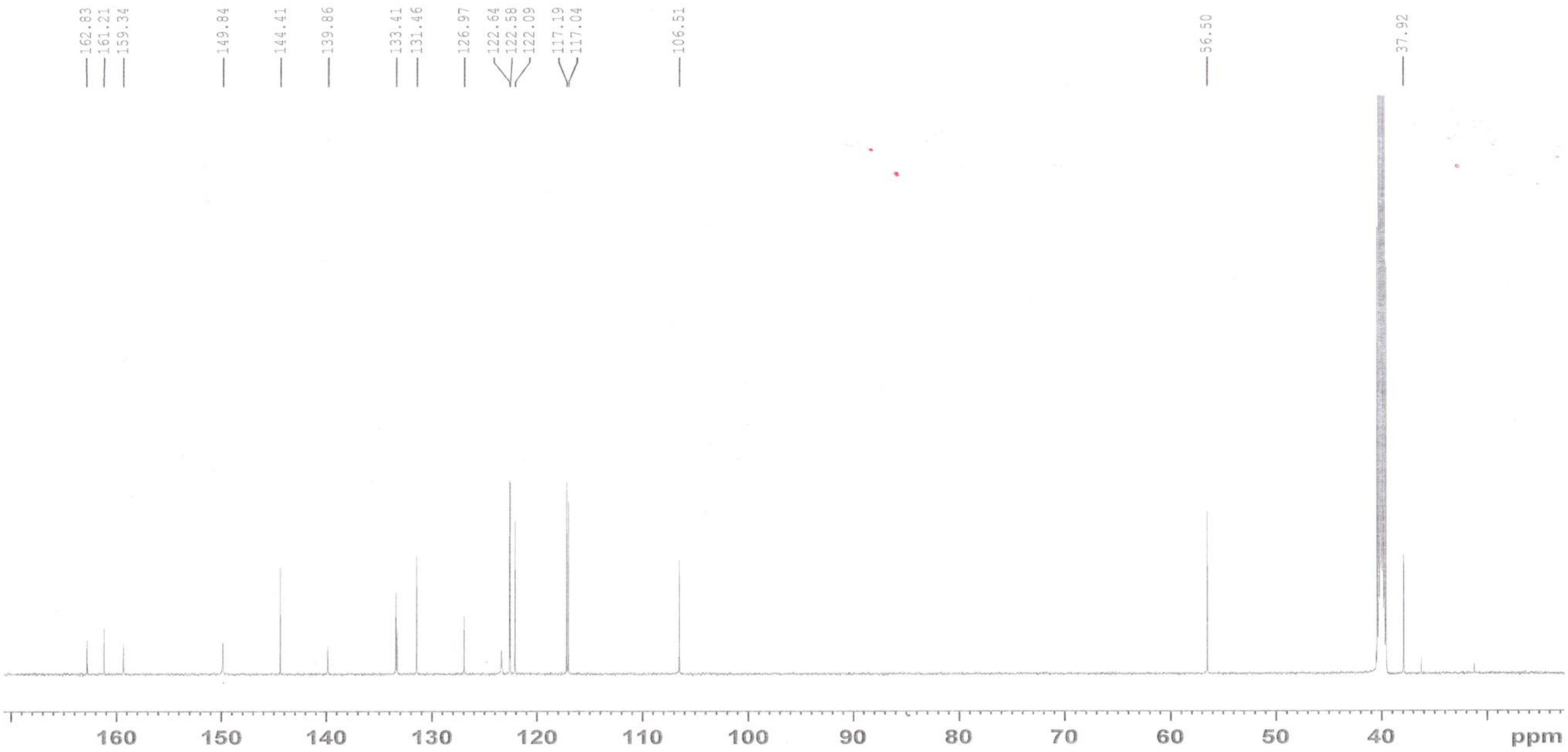

Figure S56. ^{13}C NMR spectrum of *N*-{[1-(4-chlorophenyl)-1*H*-1,2,3-triazol-4-yl]methyl}-8-methoxyquinoline-5-sulfonamide (**7d**) in DMSO.

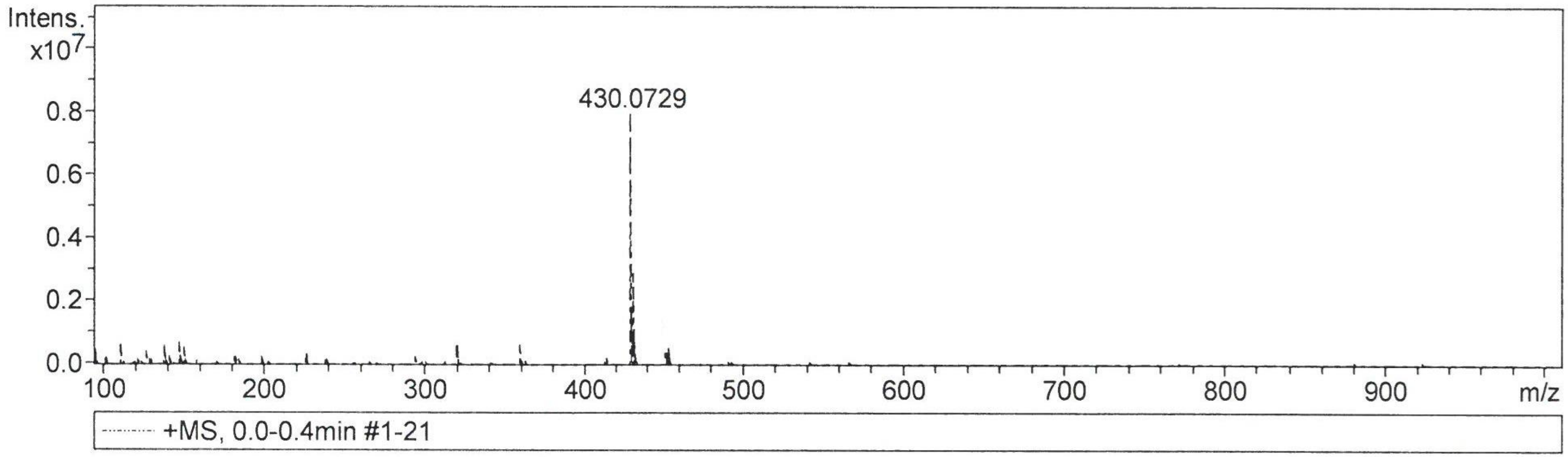

#	m/z	Res.	S/N	I	I %	FWHM
1	430.0729	43787	17640.4	7943876	100.0	0.0098
2	432.0704	36989	6004.6	2719869	34.2	0.0117

Figure S57. HR-MS spectrum of *N*-{[1-(4-chlorophenyl)-1*H*-1,2,3-triazol-4-yl]methyl}-8-methoxyquinoline-5-sulfonamide (**7d**).

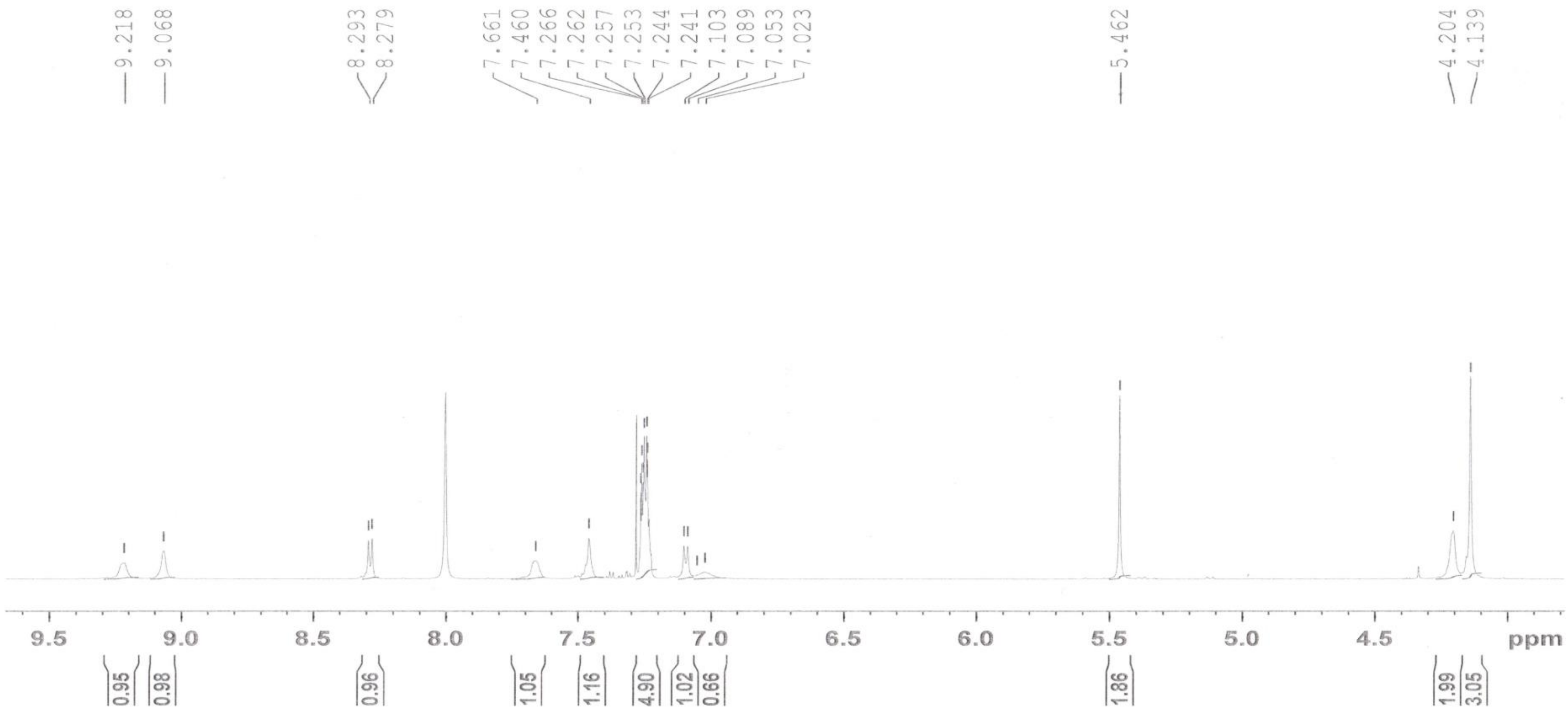

Figure S58. ^{1}H NMR spectrum of *N*-({1-[(phenylthio)methyl]-1*H*-1,2,3-triazol-4-yl}methyl)-8-methoxyquinoline-5-sulfonamide (**7e**) in $CDCl_3$.

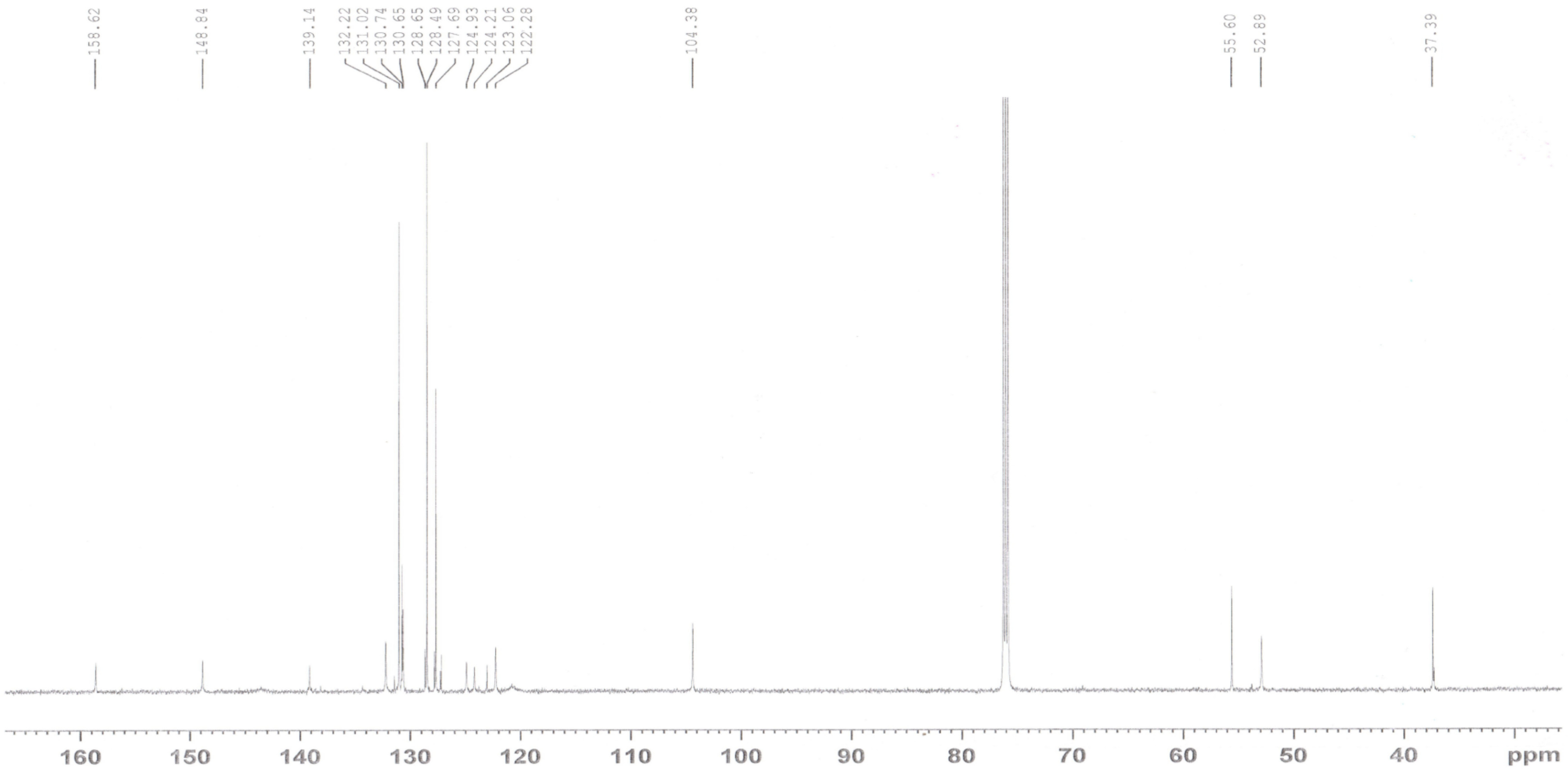

Figure S59. ^{13}C NMR spectrum of *N*-({1-[(phenylthio)methyl]-1*H*-1,2,3-triazol-4-yl}methyl)-8-methoxyquinoline-5-sulfonamide (**7e**) in $CDCl_3$.

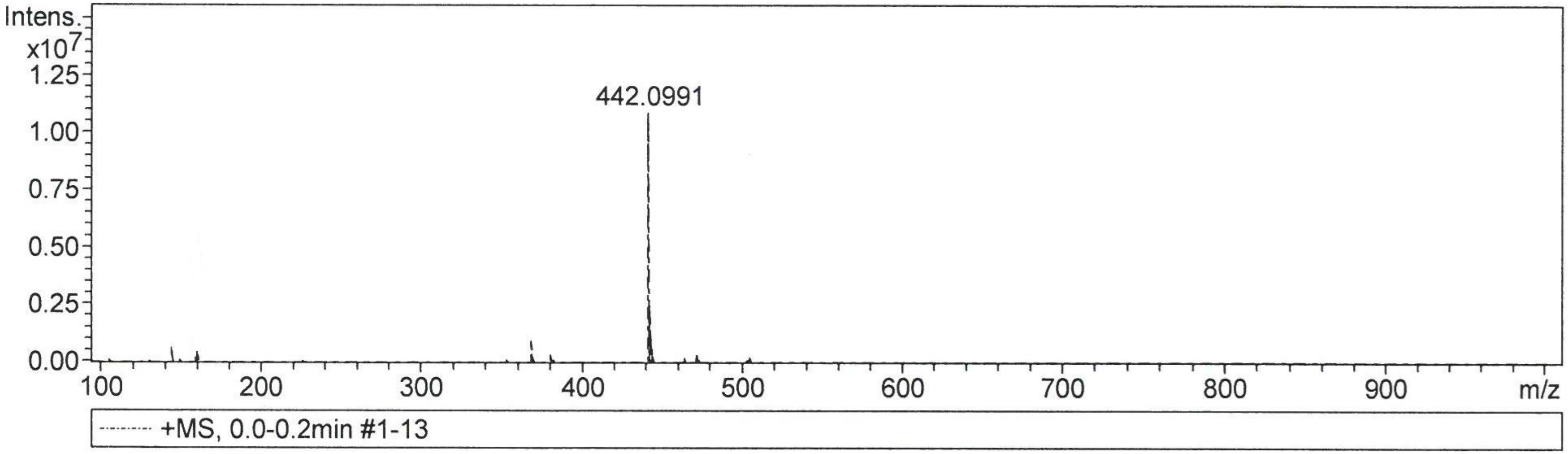

#	m/z	Res.	S/N	I	I %	FWHM
1	442.0991	44924	24854.2	10933890	100.0	0.0098

Figure S60. HR-MS spectrum of *N*-({1-[(phenylthio)methyl]-1*H*-1,2,3-triazol-4-yl}methyl)-8-methoxyquinoline-5-sulfonamide (**7e**).

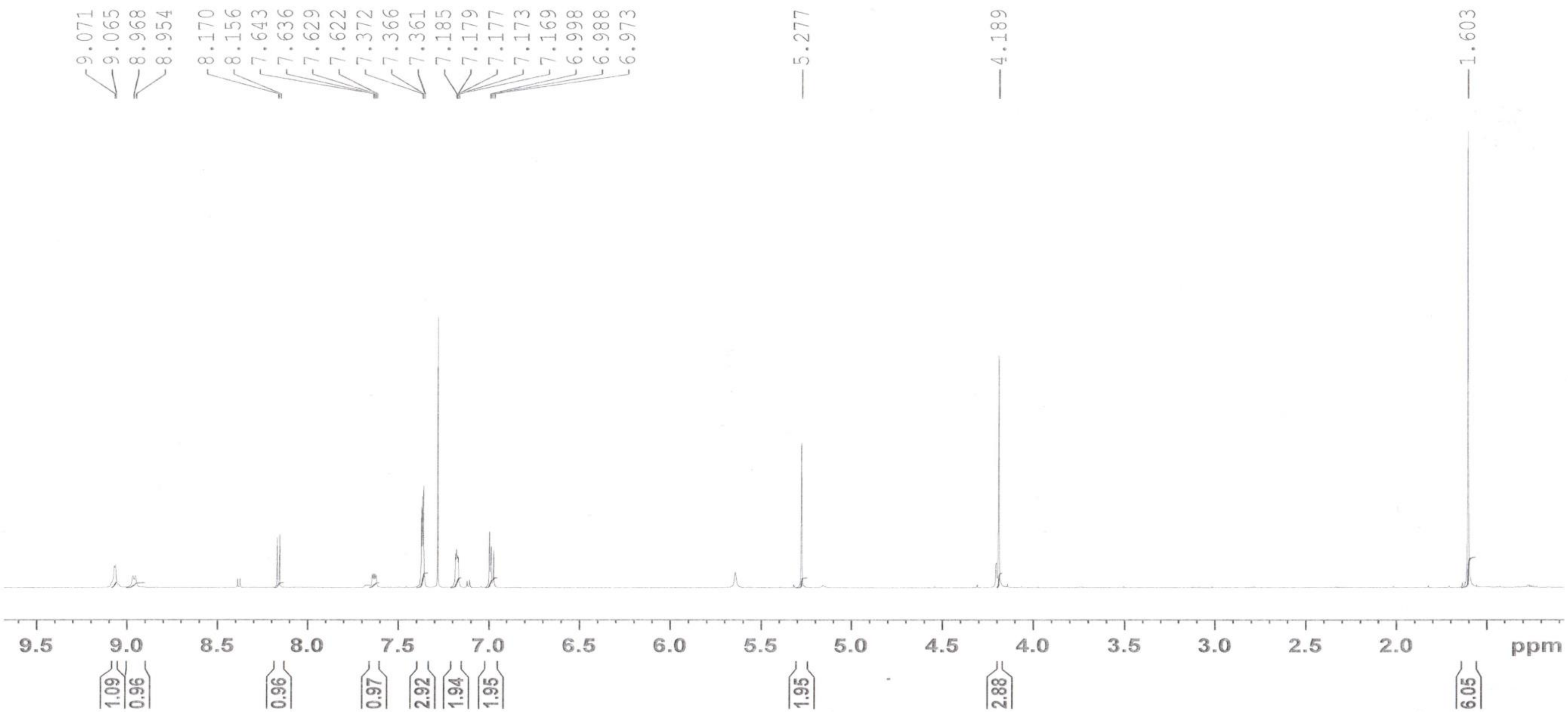

Figure S61. ^{1}H NMR spectrum of *N*-[2-(1-benzyl-1*H*-1,2,3-triazol-4-yl)propan-2-yl]-8-methoxyquinoline-5-sulfonamide (**7f**) in $CDCl_3$.

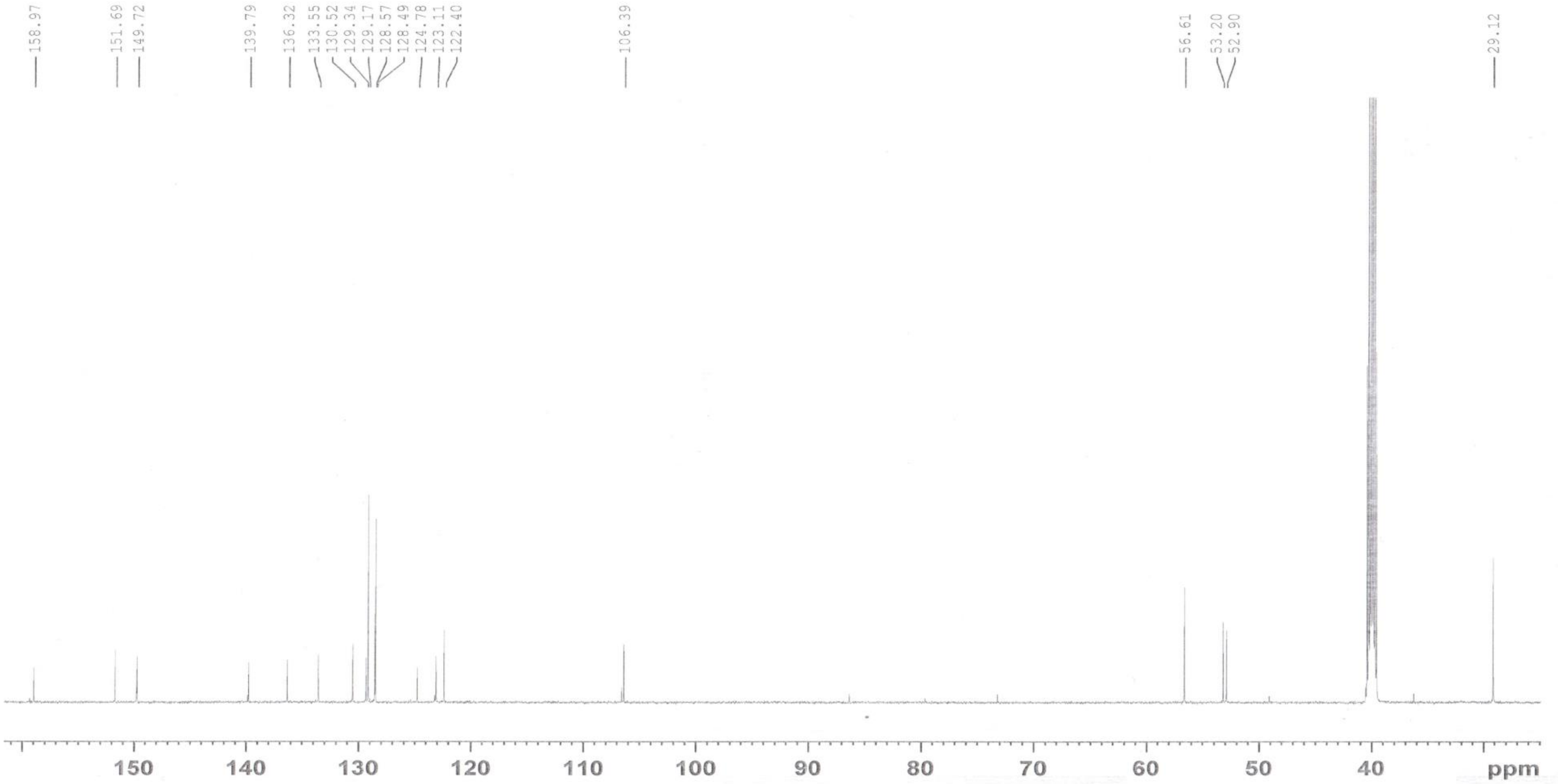

Figure S62. ^{13}C NMR spectrum of *N*-[2-(1-benzyl-1*H*-1,2,3-triazol-4-yl)propan-2-yl]-8-methoxyquinoline-5-sulfonamide (**7f**) in DMSO.

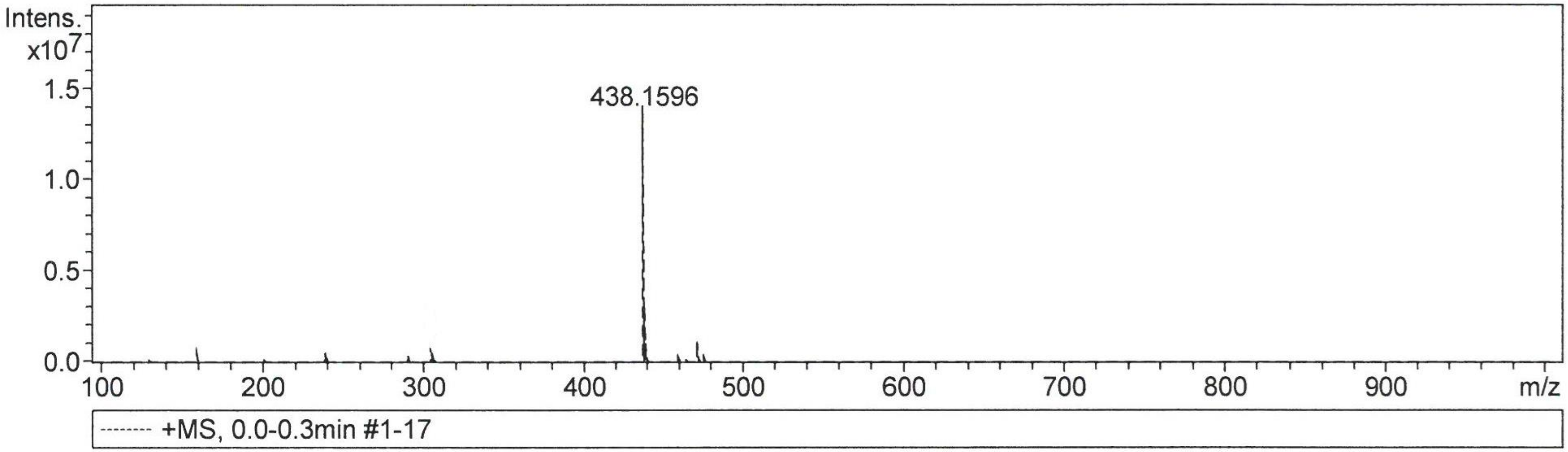

#	m/z	Res.	S/N	I	I %	FWHM
1	438.1596	45154	33660.2	13716792	100.0	0.0097

Figure S63. HR-MS spectrum of *N*-[2-(1-benzyl-1*H*-1,2,3-triazol-4-yl)propan-2-yl]-8-methoxyquinoline-5-sulfonamide (**7f**).

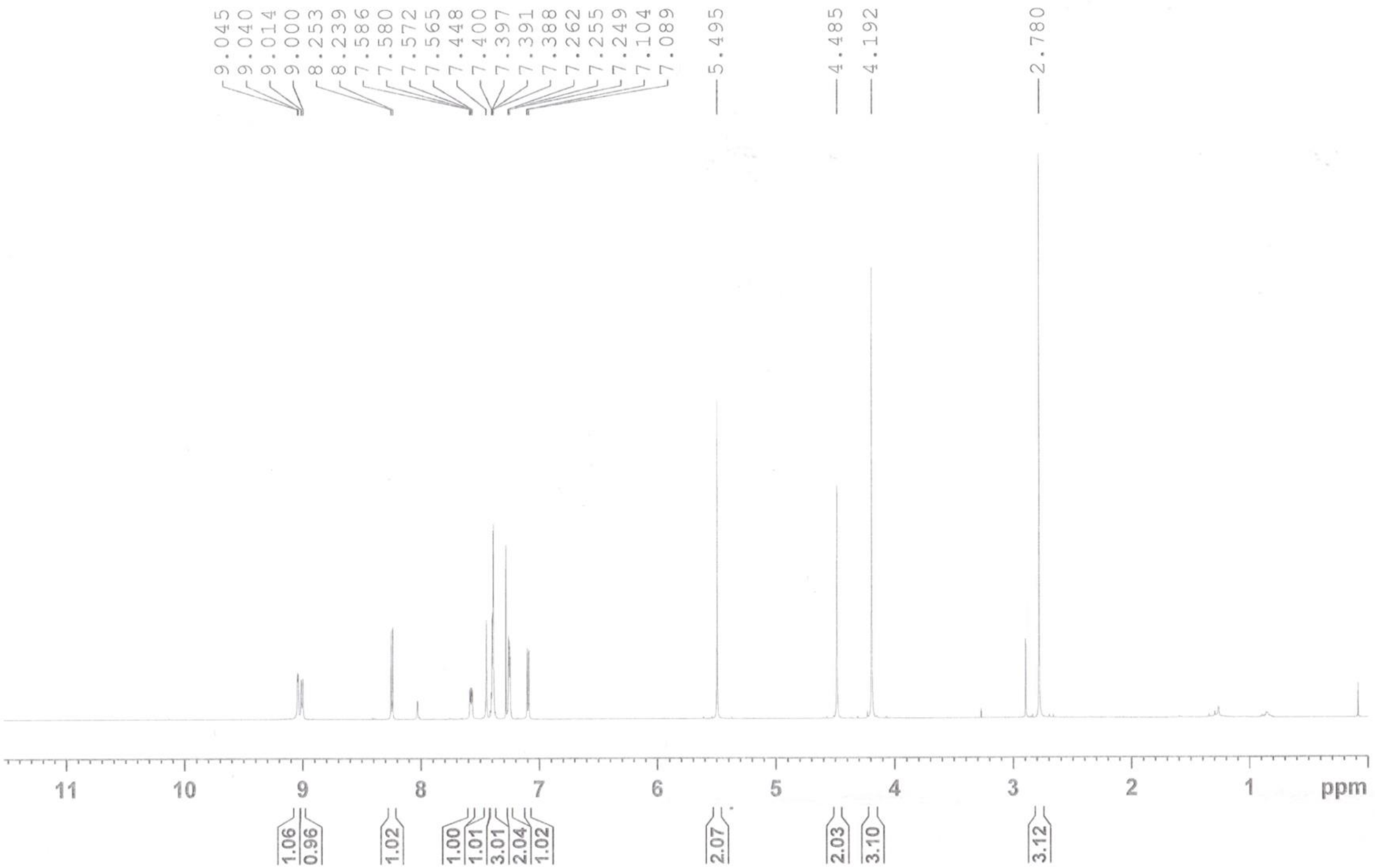

Figure S64. ^{1}H NMR spectrum of *N*-methyl-*N*-[(1-benzyl-1*H*-1,2,3-triazol-4-yl)methyl]-8-methoxyquinoline-5-sulfonamide (**7g**) in $CDCl_3$.

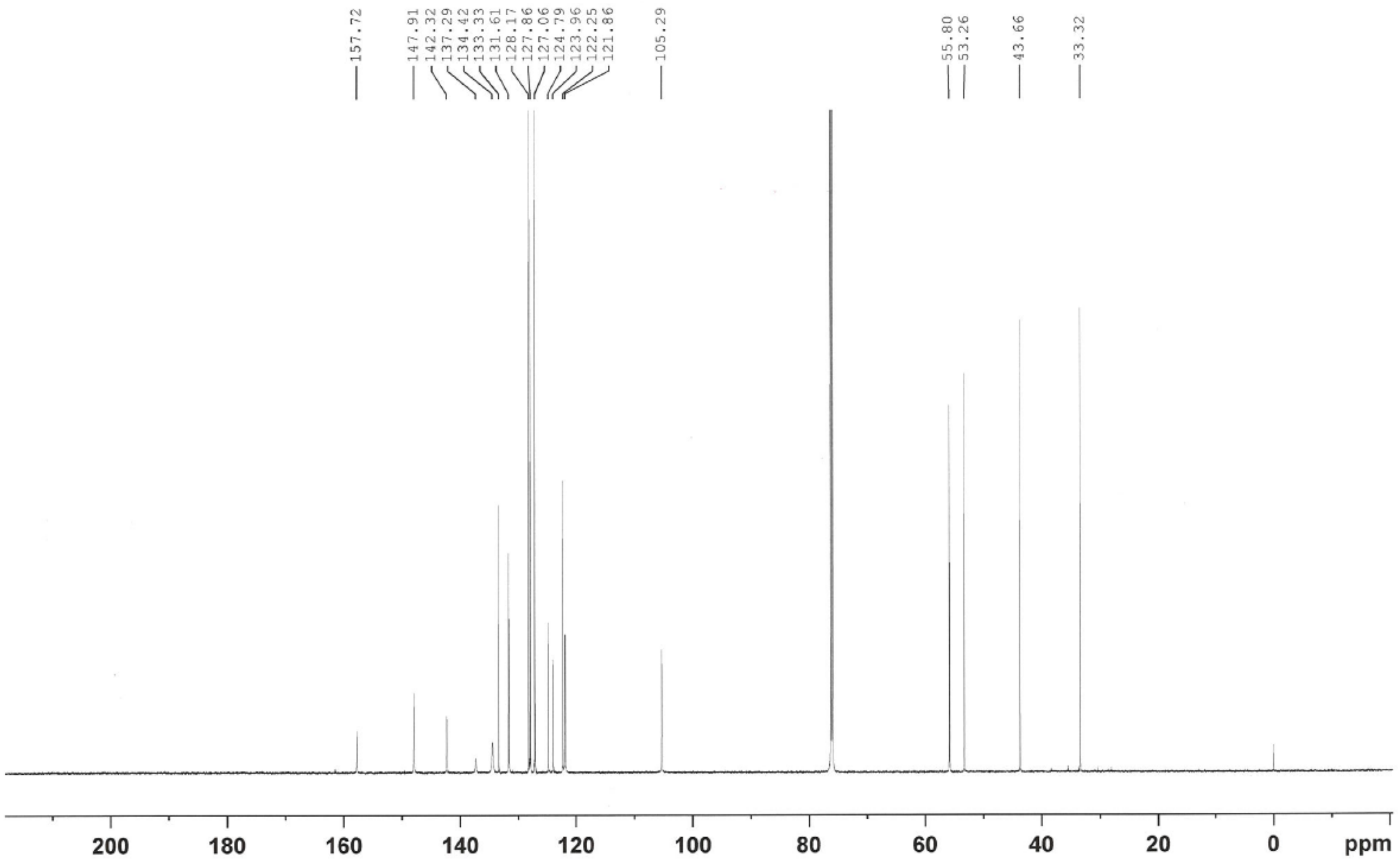

Figure S65. ^{13}C NMR spectrum of *N*-methyl-*N*-[(1-benzyl-1*H*-1,2,3-triazol-4-yl)methyl]-8-methoxyquinoline-5-sulfonamide (**7g**) in $CDCl_3$.

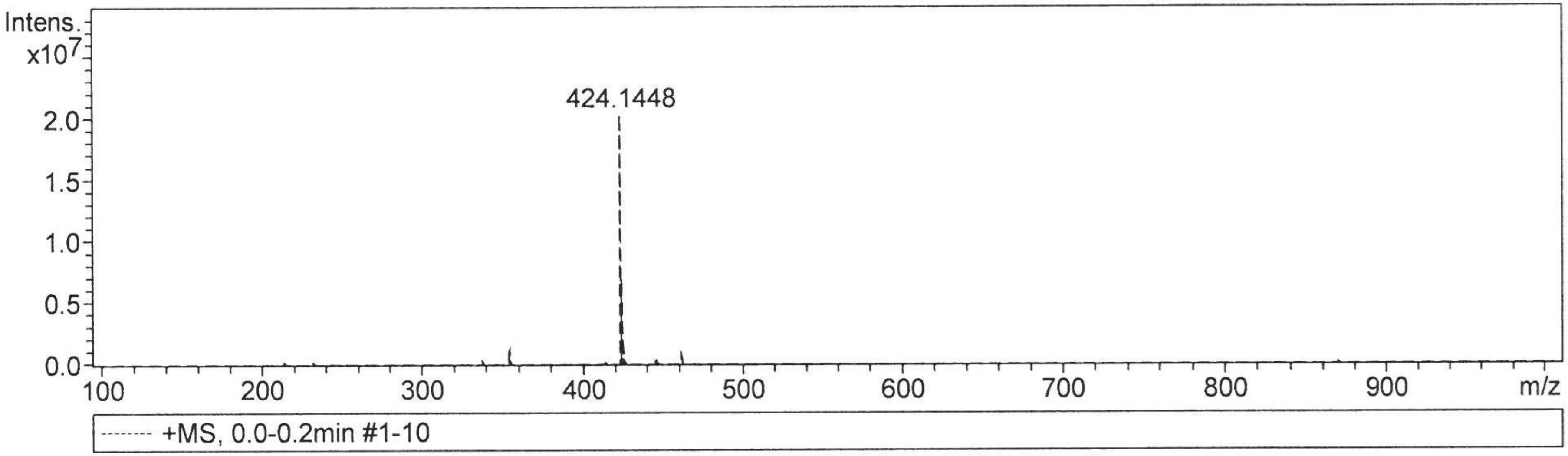

#	m/z	Res.	S/N	I	I %	FWHM
1	424.1448	21008	42953.9	20372840	100.0	0.0202

Figure S66. HR-MS spectrum of *N*-methyl-*N*-[(1-benzyl-1*H*-1,2,3-triazol-4-yl)methyl]-8-methoxyquinoline-5-sulfonamide (**7g**).

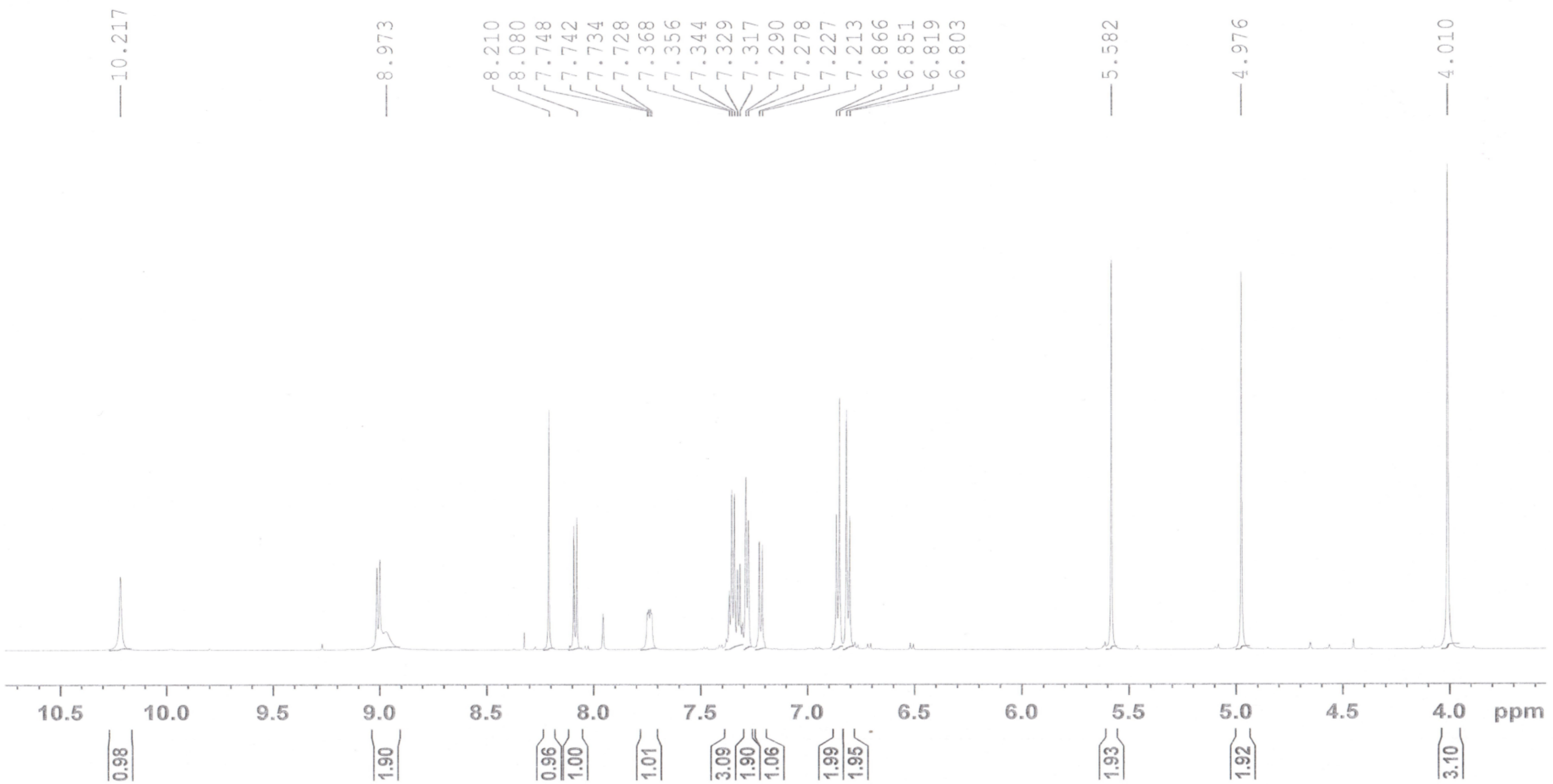

Figure S67. ^{1}H NMR spectrum of *N*-{4-[(1-benzyl-1*H*-1,2,3-triazol-4-yl)methoxy]phenyl}-8-methoxyquinoline-5-sulfonamide (**7h**) in DMSO.

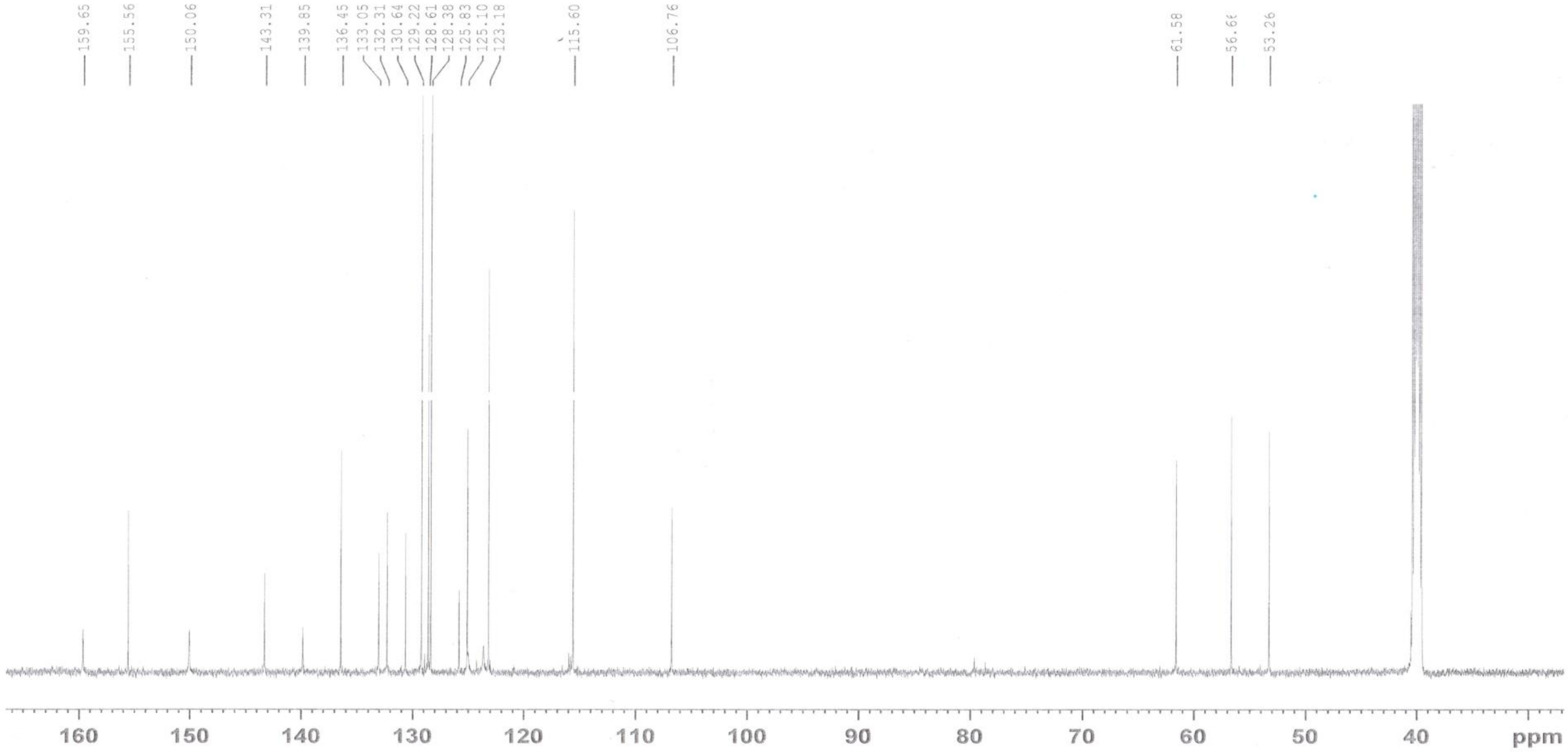

Figure S68. ^{13}C NMR spectrum of *N*-{4-[(1-benzyl-1*H*-1,2,3-triazol-4-yl)methoxy]phenyl}-8-methoxyquinoline-5-sulfonamide (**7h**) in DMSO.

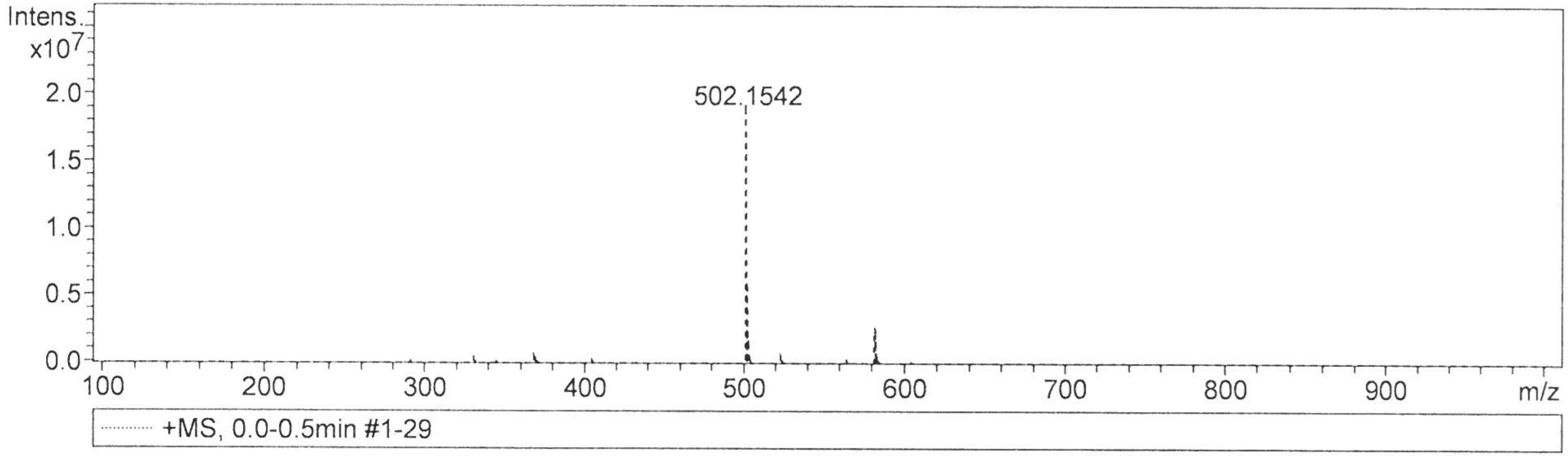

#	m/z	Res.	S/N	I	I %	FWHM
1	502.1542	30932	65126.9	18722270	100.0	0.0162

Figure S69. HR-MS spectrum of *N*-{4-[(1-benzyl-1*H*-1,2,3-triazol-4-yl)methoxy]phenyl}-8-methoxyquinoline-5-sulfonamide (**7h**).
